# SMARCC1 Enters the Nucleus via KPNA2 and Plays an Oncogenic Role in Bladder Cancer

**DOI:** 10.3389/fmolb.2022.902220

**Published:** 2022-05-20

**Authors:** Zhengmao Wei, Jinming Xu, Weiqing Li, Longhua Ou, Yingchen Zhou, Yan Wang, Bentao Shi

**Affiliations:** ^1^ Department of Urology, Peking University Shenzhen Hospital, Shenzhen, China; ^2^ Department of Urology, Shenzhen Second People’s Hospital, The First Affiliated Hospital of Shenzhen University, Shenzhen, China; ^3^ Karamay Central Hospital of Xinjiang, Karamay, China; ^4^ Department of Surgery, Fuwai Hospital Chinese Academy of Medical Sciences Shenzhen, University of South China, Shenzhen, China

**Keywords:** SMARCC1, KPNA2, bladder cancer, prognostic, nucleocytoplasmic transport

## Abstract

**Background:** SWI/SNF-related, matrix-associated, actin-dependent regulator of chromatin subfamily C member 1 (SMARCC1), a component of the SWI/SNF complex, is thought to be an oncogene in several kinds of cancer.

**Materials and methods:** The potential interaction between SMARCC1 and KPNA2 was inquired by Spearman’s correlation analysis, immunofluorescence staining and co-immunoprecipitation (Co-IP) assays. The immunohistochemistry staining, RT-PCR and western blot assay were taken for determining the expression levels of SMARCC1. And CCK-8, transwell assay, cell apoptosis assay, cell cycle analysis and subcutaneous tumor model were conducted to explore the role of SMARCC1 in carcinogenesis of bladder cancer.

**Results:** In our experiments, Spearman’s correlation analysis, immunofluorescence staining and co-immunoprecipitation (Co-IP) assays showed that SMARCC1 interacted with KPNA2, and after knockdown of KPNA2, Nup50 and Nup153, the nuclear content of SMARCC1 decreased while the amount of SMARCC1 protein remaining in the cytoplasm increased, indicating that SMARCC1 could be transported into the nucleus via KPNA2 and thus acted as an oncogene. We found that both the mRNA and protein expression levels of SMARCC1 were increased in bladder cancer, and increased SMARCC1 expression was significantly associated with a higher T stage and poorer prognosis in bladder cancer patients. Knockdown of SMARCC1 slowed the growth of the two tested cell lines and clearly arrested the cell cycle at the G0/G1 phase checkpoint. Moreover, the migratory ability was significantly decreased and the number of apoptotic cells was increased.

**Conclusion:** On the whole, our results demonstrate KPNA2, Nup50 and Nup153 regulate the process of SMARCC1 nuclear translocation in BC. SMARCC1 may be a competent candidate as a diagnostic and therapeutic target for BC. Further studies are required to research the mechanism and assess the role of SMARCC1 *in vivo*.

## 1 Introduction

Bladder cancer (BC) is a common malignant tumor of the urinary system and is the ninth most common malignant disease and the 13th most common cause of cancer death worldwide (Sanli et al., 2017) In 2018, an estimated 549,000 new cases and 200,000 deaths were ascribed to BC. BC is more common in men than in women; the incidence of BC in men is approximately 4 times that in women (Bray et al., 2018). In addition, the incidence of BC is increasing annually (Wirth et al., 2009). Transitional cell carcinoma, the most common histopathologic subtype of BC, accounts for approximately 80% of BC cases (Fleshner et al., 1996). According to the depth of the tumor invasion, BC can be divided into non-muscle-invasive BC (NMIBC), which accounts for approximately 70% of BC cases, and muscle-invasive BC (MIBC) (Stein et al., 2001). The diagnosis of BC depends on cystoscopy and a pathological analysis of samples. Currently, surgery, intravesical injection of antitumor drugs and adjuvant chemotherapy are the primary treatment regimens for BC. However, BC has a high tendency to recur; indeed, the recurrence risk is higher than 50% (Clark et al., 2013), resulting in a poor prognosis. The median survival time of patients with advanced and metastatic BC is 14 months (Bajorin et al., 1999). Thus, it is necessary to better understand the molecular mechanism of BC.

Karyopherin Alpha 2 (KPNA2), also called RAG Cohort 1, Importin Alpha 1, was first discovered by Komeili and O'Shea (Komeili and O’Shea 2000). KPNA2 is located on chromosome 17q24.2 and belongs to the Karyopherin family, a family of nuclear transporter proteins widely distributed in mammalian cells. KPNA2 mediates the nuclear translocation of a variety of important proteins, affects protein-related functions through the regulation of subcellular localization, and plays a role in signal transduction from the extracellular environment to the nucleus via the classical protein import mechanism of the importin α/β complex (Dahl et al., 2006). In our previous study, we identified that high KPNA2 expression in BC promoted BC progression and metastasis by mediating tumor cell proliferation, migration and apoptosis. Furthermore, KPNA2 was shown to be involved in the nuclear translocation of OCT4 and to affect the biological behavior of human BC cells by regulating the expression of OCT4 (Shi et al., 2015; Zhou et al., 2016). In our preliminary LM-MS analysis (data not shown), we found many potential cargo proteins of KPNA2 in BC cells. Among them, the stem cell differentiation-related protein SWI/SNF-related, matrix-associated, actin-dependent regulator of chromatin subfamily C member 1 (SMARCC1) drew our interest.

SMARCC1, a component of the SWI/SNF complex, which was originally identified in yeast, remodels the nucleosome structure in an ATP-dependent manner (Phelan et al., 1999). The SWI-SNF complex plays a key role in the regulation of eukaryotic gene expression, participating in transcriptional activation and inhibition of selected genes through chromatin remodeling (Carlson and Laurent 1994; Bochar et al., 2000). SMARCC1 can promote the differentiation of mouse embryonic stem cells (mESCs) by coupling gene inhibition with global and local changes in chromatin structure to downregulate the expression of Nanog (Schaniel et al., 2009). During T cell activation, the SWI/SNF complex is recruited to the promoter of the transcription factor AP-1, which results in upregulation of AP-1 expression and eventually promotes the activation and proliferation of T cells (Jeong et al., 2010). To date, SMARCC1 has been reported to be overexpressed in various cancers. CARM1-mediated SMARCC1 methylation is an independent prognostic biomarker for the recurrence and metastasis of breast cancer (Wang et al., 2014). Upregulation of SMARCC1 can reverse the inhibitory effect of miR-202-5p on the growth and metastasis of colorectal cancer cells (Ke et al., 2018). The overall survival of patients with high SMARCC1 protein levels was significantly poorer than that of patients with low SMARCC1 protein level-related dysregulation of the Wnt signaling pathway (Andersen et al., 2009). Furthermore, upregulation of SMARCC1 has been identified in several solid human cancers, including prostate cancer and myxoid liposarcoma (Heebøll et al., 2008; Yu et al., 2019). However, the gene expression level, biological function and molecular mechanism of SMARCC1 in BC have not been determined.

Here, we report that SMARCC1 is a cargo protein of KPNA2 in BC cells. The expression of SMARCC1 is frequently increased in clinical BC tissues and cell lines and is related to the clinicopathological characteristics of patients with BC. In addition, we analyzed the effects of SMARCC1 on the proliferation, apoptosis, cell cycle and migration of BC cells. These results provide a theoretical basis for the accurate diagnosis and targeted treatment of BC.

## 2 Material and Methods

### 2.1 Cell Culture and Bladder Cancer Tissue Specimens

The normal human bladder cell line SV-HUC-1 and human BC-derived cell lines (5637, T24, TCCSUP, SW780, J82, and UM-UC-3) were obtained from the American Type Culture Collection (Manassas, VA, United States). The SV-HUC-1 cell line was cultured in Ham’s F-12K (Kaighn’s) medium (Gibco). The 5637 cell line was cultured in RPMI-1640 medium (Gibco). The T24 cell line was cultured in McCoy’s 5A medium (Gibco) supplemented with 10% fetal bovine serum (FBS; Gibco). The TCCSUP, J82 and UM-UC-3 cell lines were cultured in minimum essential medium (Gibco). All media mentioned above were supplemented with 10% FBS (Gibco), 100 U/ml penicillin and 100 U/ml streptomycin. Cells were incubated in a humidified atmosphere containing 5% CO_2_ in air at 37°C. All tissue specimens including 30 BC tissues and 30 adjacent normal tissues for RT-qPCR were obtained from BC patients without undergoing radiotherapy and chemotherapy diagnosed at the Department of Urology, Peking University Shenzhen Hospital, China. All experiments followed the “Helsinki Declaration” and were approved by the Ethics Committee of Peking University Shenzhen Hospital. All patients were informed of their specimens content, potential risks, purpose and signed written informed consent.

### 2.2 Immunohistochemistry Staining and Scoring

Tissue microarrays containing 54 human BC specimens were purchased from Shanghai Outdo Biotech Company. The microarrays were analyzed with an SP-9000 (Rabbit) immunohistochemistry (IHC) Kit (ZSGB-BIO, China) and stained with the DAB stain supplied in the kit. Paraffin sections were immersed in fresh xylene 3 times for 10 min each and were rehydrated through a concentration gradient of ethanol solutions (100, 95, 70 and 50%; 3 times for 3 min each). After removing the excess liquid, the slides were rinsed with distilled water for 1 min and placed in phosphate-buffered saline (PBS) buffer. Antigens in the tissues were retrieved by boiling in EDTA antigen retrieval buffer (pH 9.0) for 20 min. Appropriate amounts of endogenous peroxidase blocking agent and normal goat serum were added to block endogenous peroxidase activity and nonspecific antigens. The tissue microarrays were incubated with an anti-SMARCC1 antibody (ab22355, Abcam, United Kingdom) at a 1:20,000 dilution overnight at 4°C. Rabbit mAb IgG (ab172730, Abcam, United Kingdom) and PBS were used as the negative control. The slides were washed with PBS and incubated with biotin-labeled goat anti-rabbit IgG polymer for 20 min at room temperature. Horseradish peroxidase (HRP)-labeled streptavidin working solution was added to the reaction system, incubated at room temperature for 15 min, and washed with PBS buffer 3 times for 3 min each time.

The proper amount of freshly prepared DAB solution was added to the slides and incubated at room temperature for the appropriate time. Tissue sections were counterstained with hematoxylin, dehydrated, and sealed in jaffeite. SMARCC1 staining was scored by assessing the staining percentage (0: 0%; 0.1: 1–9%; 0.5: 10–49%; 1.0: >50%) and staining intensity (on a scale of 0–3; 0: no staining; 1: weak staining, light yellow; 2: moderate staining, yellowish-brown; 3: strong staining, brown). The final semiquantitative IHC score was obtained by multiplying the nuclear staining percentage score and the nuclear staining intensity score. The median of all IHC scores was preferentially selected as the cutoff point for separating SMARCC1-positive tumors from SMARCC1-negative tumors.

### 2.3 Immunofluorescence Staining

1 × 10^4^ cells were seeded on chamber slides in 24-well plate for 24 h. After being washed with PBS, cells were fixed in 4% paraformaldehyde, permeated with 0.5% TritonX-100 and blocked in PBS containing 1% BSA for 30 min at room temperature. Slides were washed and then incubated with primary antibodies against SMARCC1 (ab172638, 1:100, Abcam, United Kingdom) and KPNA2 (ab70160, 1:100, Abcam, United Kingdom) at 4°C overnight. Fluorescence-conjugated secondary antibodies Alexa Fluor™ 488 (A21206, 1:1,000, Invitrogen, United States) and Alexa Fluor™ 594 (A32754, 1:1,000, Invitrogen, United States) were used as secondary antibodies. Cell nuclei were counterstained with 5 ug/ml DAPI, and fluorescence was captured with a microscope.

### 2.4 Extraction of Cytoplasmic Protein and Nuclear Protein

Adherent cells were cleaned with PBS, scraped off with cell scrapers and centrifuged. The supernatant was sucked up, and 200 ul of cytoplasmic protein extraction reagent A (Beyotime Technology) with PMSF was added to every 20 ul of cell precipitates. The cell precipitates were completely suspended and dispersed in a vortex for 5 s. After ice bath for 10 min, 10 ul of cytoplasmic protein extraction reagent B was added. Then, vortex 5 s, ice bath for 1min and vortex 5 s again, 12,000 g centrifugation at 4°C for 5 min. The supernatant was namely the extracted cytoplasmic protein. For the rest of the precipitates, 50 ul of nuclear protein extraction reagent was added, and the precipitates were completely suspended and separated by vortex at maximum speed for 15 s. Next, vortex 15 s was conducted after ice bath every 1–2 min, which would last for 30 min in total. Finally, centrifuged at 12,000 g at 4°C for 10 min, the supernatant was namely the extracted nuclear protein. The cytoplasmic and nuclear proteins were frozen at −80°C for further detection.

### 2.5 Protein Extraction and Western Blot Analysis

Cells were lysed and total protein was extracted with RIPA lysis buffer (Beyotime Technology). The total protein concentration was determined with a BCA kit (Thermo Fisher). The protein samples were boiled at 95°C for 5 min, separated by 10% SDS-PAGE, and transferred to 0.45 μm PVDF membranes (Millipore). After incubation with primary antibodies overnight at 4°C, these membranes were washed with TBST buffer and incubated with secondary antibodies at room temperature. The primary antibodies were SMARCC1 (ab172638, 1:1,000, Abcam, United Kingdom), KPNA2 (ab70160, 1:10,000, Abcam, United Kingdom), NUP50 (ab151567, 1:2000, Abcam, United Kingdom), NUP153 (ab171074, 1:1,000, Abcam, United Kingdom) and β-tubulin (ab6046, 1:5000, Abcam, United Kingdom). The secondary antibodies were anti-rabbit IgG (7074,1:2000, Cell Signaling Technology, United States) and anti-mouse IgG (7076, 1:2000, Cell Signaling Technology, United States) HRP linked antibody. Immunoreactive bands were visualized by enhanced chemiluminescence using HRP Substrate Peroxide (Millipore).

### 2.6 RNA Extraction and RT-PCR

Total RNA from tissue-cultured cells was isolated using TRIzol reagent (Invitrogen, Carlsbad, CA, United States). 1 µg of total RNA from specimens was reversely transcribed using a PrimeScript™ RT reagent Kit with gDNA Eraser (Takara, Japan). Real-time qPCR was performed using SYBR Premix Ex Taq™ II Kit (Takara, Japan) in LightCycler 480 (Roche, United States). Expression levels were normalized to those of GAPDH as the endogenous control. The relative mRNA expression levels were determined using the -ΔΔCt or 2^−ΔΔCt^ method. The primer sequences were as follows: SMARCC1 primers, forward: 5′-TGT​TGG​AAG​TCG​TAC​TCA​GGA​TG-3′ and reverse: 5′-TGG​ATT​TCC​TGA​CTG​ACT​GAA​GG-3′; GAPDH primers, forward: 5′-CCA​CTC​CTC​CAC​CTT​TGA​CG-3′ and reverse: 5′-CTG​GTG​GTC​CAG​GGG​TCT​TA-3′.

### 2.7 Transfection of siRNA

SW780 and UMUC-3 BC cells were transfected with 100 nM siRNA oligonucleotides using Lipofectamine 3000 following the manufacturer’s protocol. The siRNAs construct targeting SMARCC1, KPNA2, NUP50 and NUP153 were ordered from Gene Pharma (Shanghai, China). The siRNAs sequences were listed as followed: siR-SMARCC1 (5′-CUC​CCU​GCA​AAG​UGU​UUC​ATT-3′), siR-KPNA2 (5′-GAC​UCA​GGU​UGU​GAU​UGA​UTT-3′), siR-NUP50 (5′-CCA​CCU​UGG​UUG​AUA​AAG​UTT-3′), siR-NUP153 (5′-GGA​CTT​GTT​AGA​TCT​AGT​T-3′) and siR-NC (5′-CAC​CGU​GAA​GCU​GAA​GGU​GTT-3′).

### 2.8 Cell Proliferation Assay

Cells transfected with specific siRNAs were collected and inoculated in 96-well plates (approximately 2000 cells per well). The change in the cell proliferation rate after transfection was evaluated by a CCK-8 assay (Dojindo Laboratories). The optical density (OD) value was measured at 450 nm using a Multiskan Go plate reader (Thermo Fisher Scientific) every 24 h until day 4. All experiments were performed in triplicate.

### 2.9 Cell Migration Assay

Cells were harvested after 24 h of transfection with siR-SMARCC1. Then, 1 × 10^5^ cells were suspended in serum-free medium and seeded in the upper compartments of the chambers (Costa 3422), while the bottom compartments of the chambers were filled with medium containing 10% FBS to detect the migratory ability of BC cells. After incubation at 37°C for 24 h, cells were fixed in 4% paraformaldehyde, stained with 0.1% crystal violet, and finally, rinsed with PBS. A cotton swab was used to wipe the stained cells from the upper surface of the membrane. Eight random visual fields per chamber were photographed, and the cells in each field were counted. All experiments were performed in triplicate.

### 2.10 Cell Apoptosis Assay

The target cells were centrifuged, washed with PBS, and then centrifuged again to separate the supernatant. The apoptosis rates of transfected SW780 and UMUC-3 cells were determined by using an Annexin V FITC Apoptosis Detection Kit (Dojindo). The cell suspension was brought to a final concentration of 1 × 106 cells/ml with 1 × Annexin V Binding Solution. Annexin V, APC conjugate (5 µl) was added to 100 µl of the cell suspension, and 5 μl of propidium iodide (PI) solution was then added. FlowJo V 10 software was used to analyze the apoptosis rate data obtained by flow cytometry. All experiments were performed in triplicate.

### 2.11 Cell Cycle Analysis

Transfected cells were harvested and washed thoroughly with ice-cold PBS two times. Cells were fixed by incubation in 1 ml of precooled 75% ethanol at 4°C overnight. The target cells were then incubated in PI/RNase Staining Buffer (BD Biosciences) for 30 min in the dark at room temperature. The DNA content was determined by flow cytometry, and FlowJo V 10 software was used to analyze the data. All experiments were repeated at least three times.

### 2.12 Coimmunoprecipitation

Proteins were extracted from SW780 and UMUC-3 cells with NP-40 cell lysis buffer containing protease inhibitors (Beyotime Biotechnology) and diluted to 1 μg/μl with PBS. The cell lysates were incubated with protein A/G agarose beads (Life Technology) and an anti-SMARCC1 antibody (Abcam PLC, Cambridge, United Kingdom) overnight at 4°C and then centrifuged at 4,000 rpm for 30 s. Next, the pellets were carefully washed with precooled NP40 lysis buffer. Finally, the protein complexes were boiled and analyzed by SDS-PAGE and western blotting with the polyclonal anti-SMARCC1 antibody or anti-KPNA2 antibody.

### 2.13 Establishment of in Vivo Animal Model

The animal experiment protocol was approved by the Animal Ethics Committee of the Medical Center of Hong Kong University of Science and Technology, Peking University, Shenzhen. Ten 4- to 6-week-old BALB/c nude mice were divided equally into two groups: the siR-NC group and the siR-SMARCC1 group. Transfected UMUC-3 cells were resuspended in 0.25 ml of PBS buffer and were then mixed with the same volume of matrix gel. Each mouse was subcutaneously inoculated with a mixture containing 2 × 10^6^ cells. At the 4^th^ week, the tumors were removed completely and weighed. Tumor volume(V) was calculated by tumor length(L) and width(W) as: V = (L × W^2^)/2.

### 2.14 Statistical Analysis

GraphPad Prism 8 (GraphPad Software, Inc., La Jolla, CA, United States) was used for the statistical analysis. Chi-square test and Fisher’s exact test were used to evaluate statistical associations between SMARCC1 expression and clinicopathological parameters. Survival curves for patients stratified by SMARCC1 expression were generated using the Kaplan-Meier method and analyzed for statistical significance using the log-rank test. All functional experiments were repeated in triplicate, the results of which were presented as the mean ± standard deviation and analyzed by independent *t*-test or one-way ANOVA. A *p* value of less than 0.05 was considered as statistical significance.

## 3 Results

### 3.1 KPNA2-Mediated Nuclear Import of SMARCC1 in BC Cells

**FIGURE 1:**
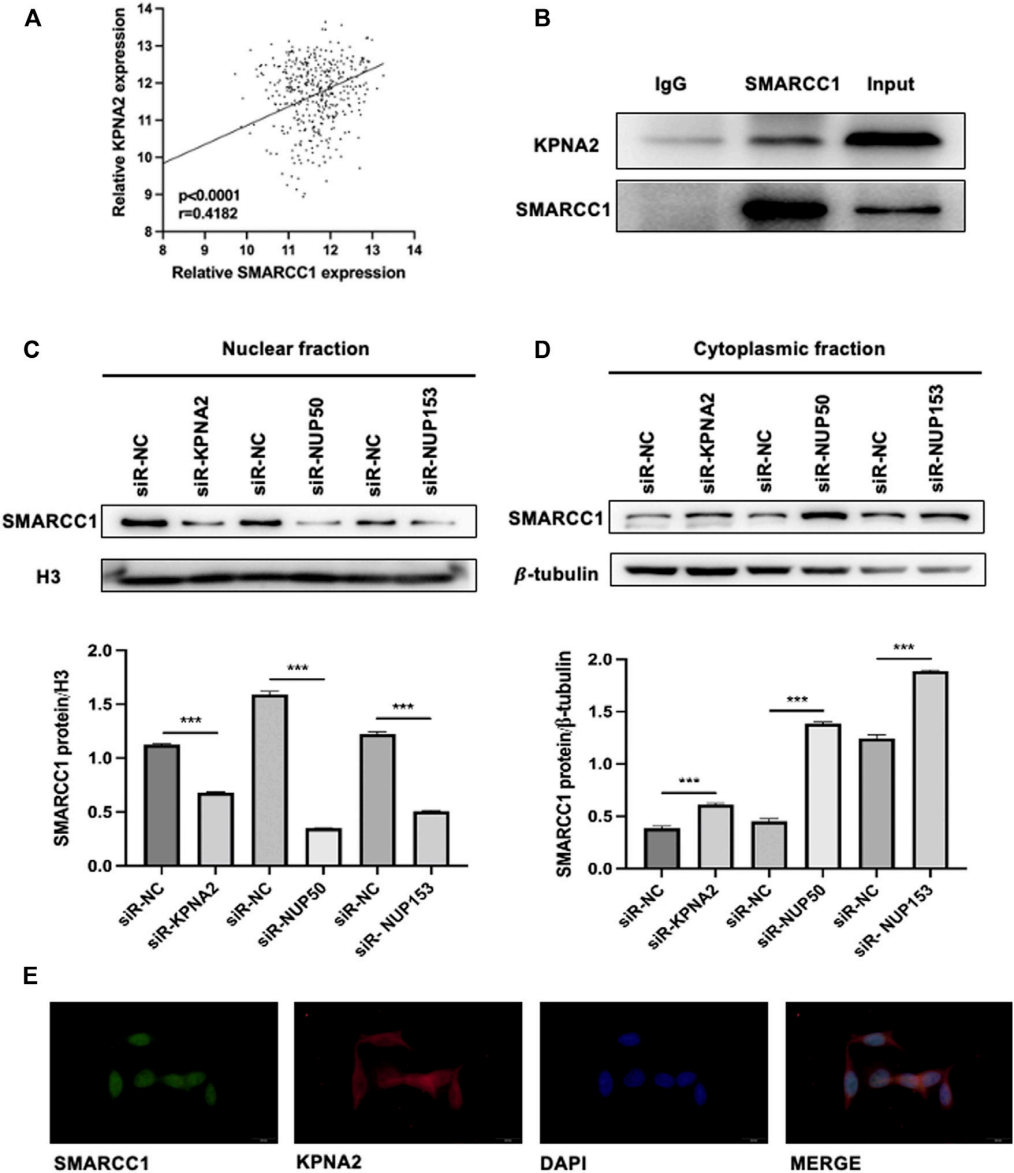
SMARCC1 is a cargo protein of KPNA2 in BC cells. **(A)** Pearson’s correlation analysis indicated a distinct positive correlation between SMARCC1 and KPNA2 expression. **(B)** A Co-IP assay was used to examine the association between KPNA2 and SMARCC1 *in vitro*. **(C–D)** After knockdown of KPNA2, Nup50 and Nup153, SMARCC1 expression was dramatically decreased in the nuclear fraction, while increased in the cytoplasmic fraction of UMUC-3 cell. **(E)** Representative immunofluorescence (IF) staining images, showing SMARCC1 (green) and KPNA2 (red) expression in the UMUC-3 cell in bladder cancer. Scale bar, 20 μm.

To investigate whether the process of SMARCC1 nuclear translocation could be facilitated by KPNA2, we analyzed SMARCC1 and KPNA2 expression in the TCGA database (https://www.cancer.gov/), which contained mRNA expression data. Pearson’s correlation analysis indicated a distinct positive correlation between SMARCC1 and KPNA2 expression (Figure 1A). The results of the Co-IP assay showed that KPNA2 and SMARCC1 interacted with each other under physiological conditions in UMUC-3 cells (Figure 1B). Furthermore, we evaluated the expression of SMARCC1 and its subcellular distribution in both cytoplasmic and nuclear fractions by western blot after knockdown of KPNA2. We found that the SMARCC1 level was dramatically decreased in the nuclear fraction when KPNA2 was silenced. Meanwhile, the cytoplasmic SMARCC1 protein level was slightly increased (Figures 1C,D). Interaction between Nup50 and Nup153 provided an important platform for nucleocytoplasmic trafficking. Thus, we extracted nuclear and cytoplasmic fractions of SMARCC1 in BC cells after knockdown of Nup50 and Nup153 by siRNA. We found that nuclear SMARCC1 was decreased, while cytoplasmic SMARCC1 was increased, which indicated that KPNA2 could mediate the nuclear import of SMARCC1 in BC cells. In addition, immunofluorescence staining results showed that most of the endogenous SMARCC1 protein was located in the nucleus and only a little of SMARCC1 protein was expressed in the cytoplasm of bladder cancer cells. And KPNA2 was co-expressed with SMARCC1 in cell nucleus of bladder cancer (Figure 1E).

### 3.2 SMARCC1 Was Highly Expressed in Bladder Cancer Tissues and Cell Lines

**FIGURE 2:**
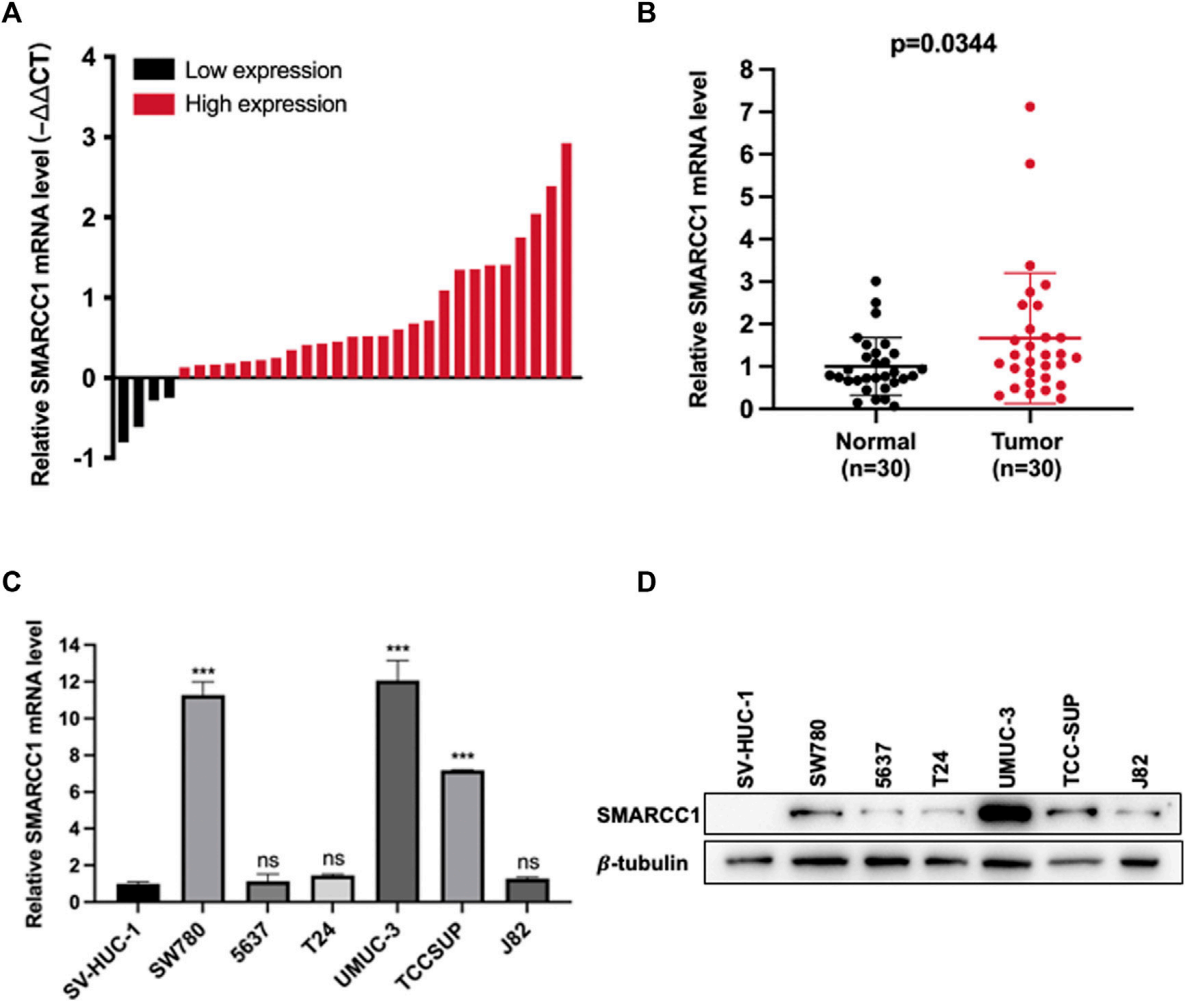
SMARCC1 was highly expressed in BC tissues and cell lines. **(A)** SMARCC1 was highly expressed in 86.7% (26/30) of the BC specimens compared with para-cancerous tissues. **(B)** The relative expression of SMARCC1 in BC tissues was significantly higher than that in adjacent normal tissues. The mean of each triplicate result was used to calculate the relative SMARCC1 concentration by using the comparative 2-∆Ct method. **(C)** The mRNA level of SMARCC1 in BC cell lines was evaluated by RT-qPCR. The mRNA level of SMARCC1 in SW780, UMUC-3, and TCCSUP cells was higher than that in SV-HUC-1 cells. GAPDH was used as the control. **(D)** The protein level of SMARCC1 in BC cell lines was measured by western blot. Compared with SV-HUC-1 cell line, the BC cell lines (SW780, UMUC-3 and TCCSUP) exhibited relatively high protein levels of SMARCC1. β-tubulin was used as the endogenous control. * *p* < 0.05, ** *p* < 0.01, *** *p* < 0.001.

We used RT-qPCR and western blot to measure the mRNA and protein levels of SMARCC1 in a cohort of 30 paired fresh BC tissues and adjacent normal tissues, and BC cell lines. As shown in Figure 2A, SMARCC1 was highly expressed in 86.7% (26/30) of the BC specimens compared with para-cancerous tissues. The relative expression of SMARCC1 in BC was significantly higher than that in adjacent normal tissues after logarithmic conversion (*p* = 0.0344) (Figure 2B). In addition, our results revealed that SMARCC1 was remarkably overexpressed in three BC cell lines (SW780, UMUC-3 and TCCSUP) compared with normal urothelial cells SV-HUC-1 (Figures 2C,D).

### 3.3 Expression of SMARCC1 Was Closely Related to T Stage and Survival of Patients With Bladder Cancer

**FIGURE 3:**
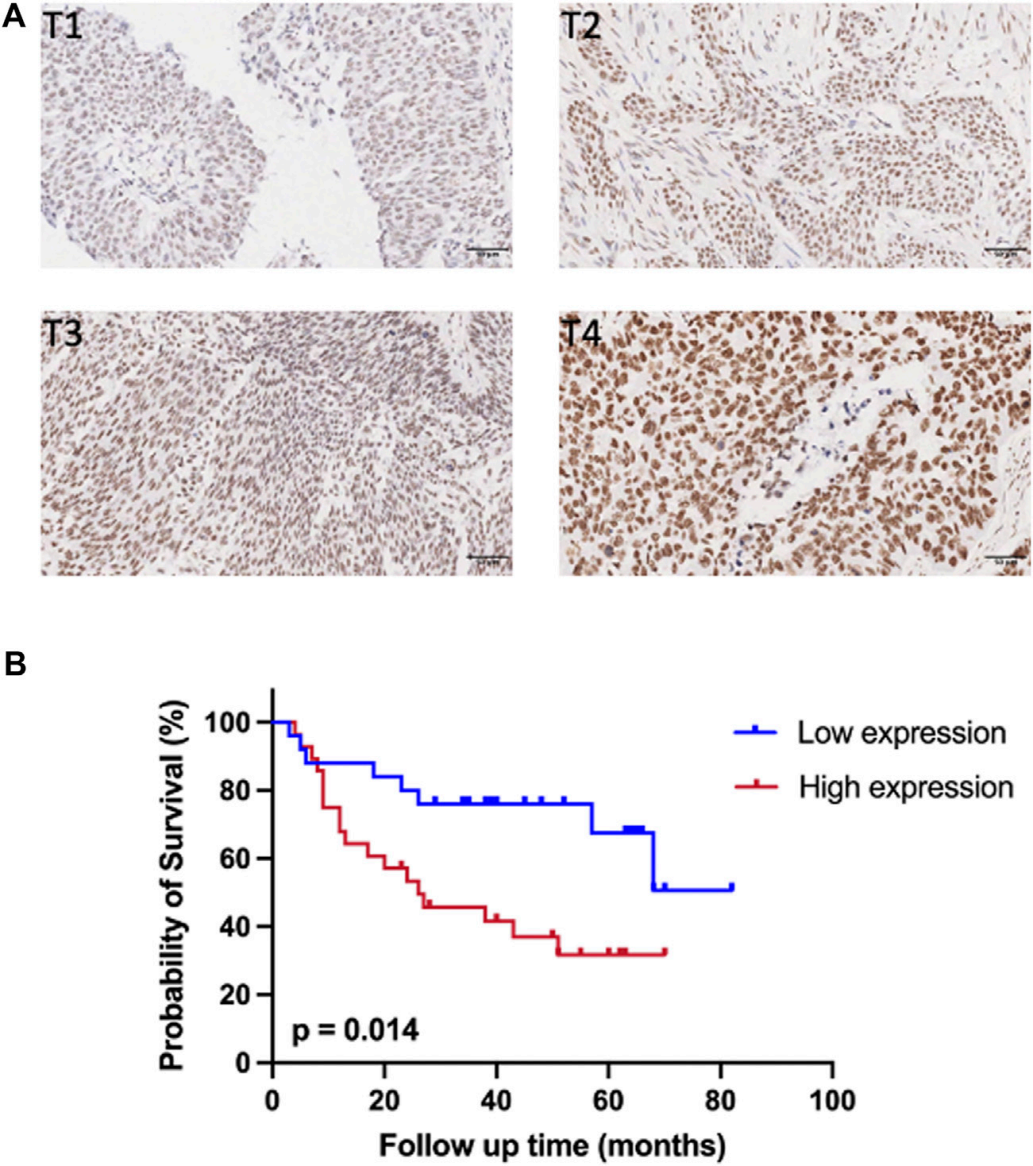
The expression of SMARCC1 was closely related to T stage and survival of patients with BC. **(A)** Representative images of SMARCC1 staining in paraffin-embedded BC tissues of stage T1-T4. The IHC analysis revealed that SMARCC1 was localized mostly in the nucleus in BC specimens, and the positive percentage and intensity of SMARCC1 staining in BC were gradually elevated along with the T stage increased. **(B)** The Kaplan-Meier method was applied to analyze the overall survival of BC patients. The survival rate of patients with low SMARCC1 expression was significantly higher than that of patients with high SMARCC1 expression (log-rank test; *p* = 0.014).

IHC staining was conducted on 54 cases of BC tissues to investigate the relationship between SMARCC1 expression and clinicopathological characteristics. The results revealed that the SMARCC1 protein was mainly detected in the cell nucleus, and the positive percentage and intensity of SMARCC1 staining in BC were gradually elevated along with the T stage increased (Figure 3A). Besides, our data demonstrated that the expression of SMARCC1 was significantly correlated with T stage (*p* = 0.007), while no significant correlation was found between SMARCC1 expression and sex, age, pathological type, clinical stage, lymph node involvement (N stage) and distant metastasis (M stage) (*p* > 0.05) (Table 1). A negative association between overall survival and SMARCC1 expression in BC was identified by Kaplan-Meier analysis. High SMARCC1 expression was significantly associated with an adverse prognosis (*p* = 0.014) (Figure 3B). The median survival time of BC patients exhibiting low and high SMARCC1 protein expression was 50 and 26 months, respectively.

**TABLE 1:** High expression levels of SMARCC1 were correlated with clinicopathological features in BC.

Clinicopathological Variables	No. of Patients	Expression of SMARCC1		*X* ^*2*^	P
		Low	High		
Sex					
Male	46	20	26	0.117	0732
Female	8	4	4		
Age					
<70	26	12	14	0.059	0.808
≥70	28	12	16		
Primary tumor stage					
Tis-T1	15	13	2	7.25	0.007*
T2-T4	37	17	20		
Pathological type					
Low-grade	16	5	11	1.603	0.205
High-grade	38	19	19		
Clinical stage					
0-I	9	3	6	1.239	0.744
II	9	5	4		
III	16	6	10		
IV	6	3	3		
Lymph node metastasis					
N0	33	3	30	0.591	0.4420
N1-N3	6	0	6		
Metastasis					
M0	47	4	43	0.461	0.659
M1	5	0	5		
*p < 0.05 was considered to indicate statistical significance					

### 3.4 Knockdown of SMARCC1 Suppressed Cell Proliferation and Induced G0/G1 Arrest

**FIGURE 4:**
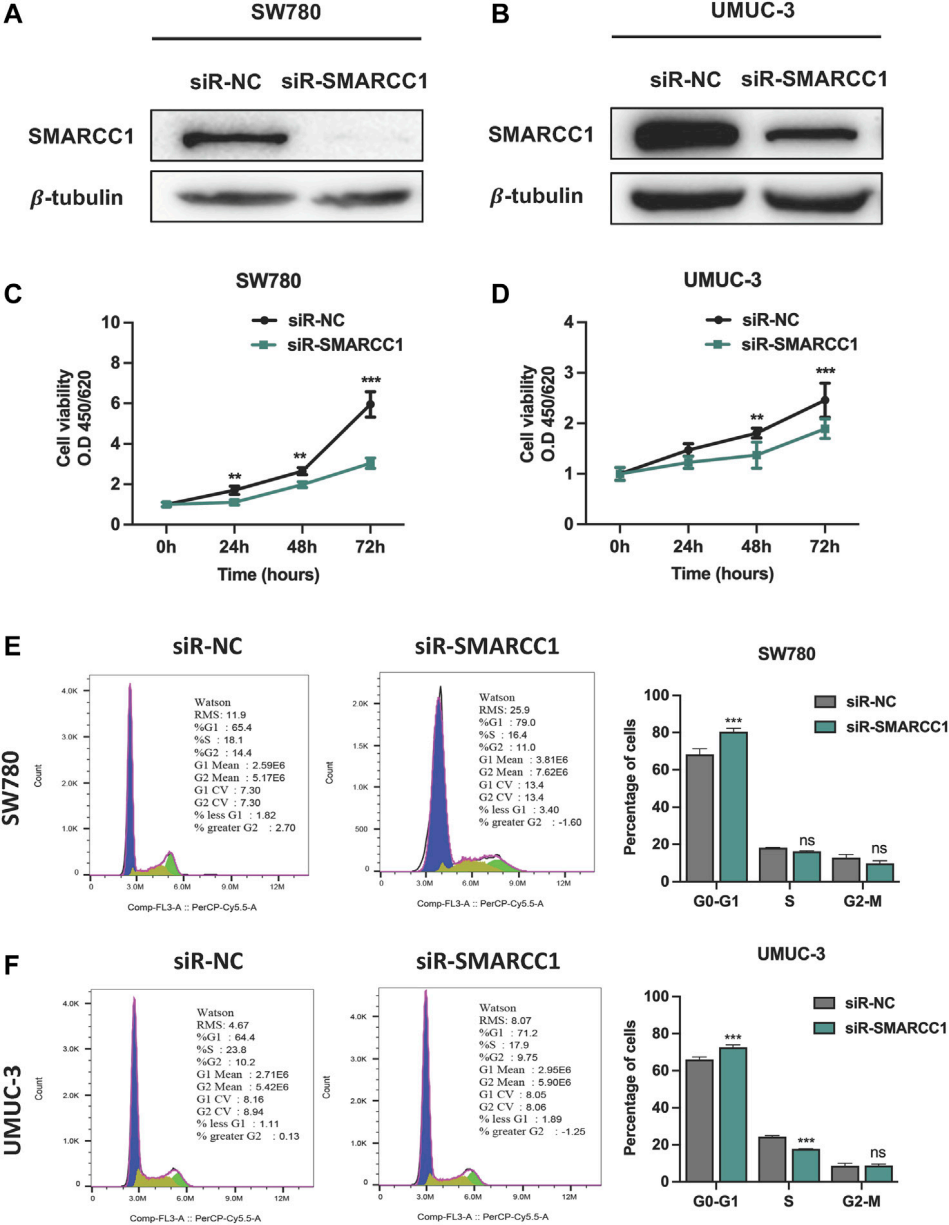
Knockdown of SMARCC1 inhibited cell proliferation and induced cell cycle arrest in G0/G1. **(A–B)** The western blot results indicated that specific siRNAs for SMARCC1 significantly decreased the expression of SMARCC1 in both SW780 and UMUC-3 cells. **(C–D)** Cell proliferation was evaluated using a CCK-8 assay. The proliferation of SW780 and UMUC-3 cells was suppressed after transfection with siR-SMARCC1. **(E–F)** Flow cytometry analysis indicated that knockdown of SMARCC1 induced cell cycle arrest in G0/G1 phase. * *p* < 0.05, ** *p* < 0.01, *** *p* < 0.001.

We selected SW780 and UMUC-3 cells for further *in vitro* investigation of the potential biological functions of SMARCC1 in BC. SW780 and UMUC-3 cells were transfected with specific siRNA for SMARCC1 (siR-SMARCC1) and negative control siRNA (siR-NC) for 48 h. Western blot was conducted to confirm the knockdown efficacy of specific siRNAs, the results of which revealed that SMARCC1 protein expression was significantly downregulated by siR-SMARCC1 (Figures 4A,B). To determine whether SMARCC1 influenced cell proliferation and cell cycle progression of BC, CCK-8 assay and flow cytometry were performed after knockdown of SMARCC1. The results demonstrated that SMARCC1 silencing markedly inhibited the proliferation of SW780 and UMUC-3 cells (Figures 4C,D). In addition, the percentage of siR-SMARCC1-transfected cells in G0/G1 phase was markedly increased compared with siR-NC-transfected cells, which indicated that down-regulated the expression of SMARCC1 induced G1/S cell cycle arrest in BC cells (Figures 4E,F).

### 3.5 Silencing SMARCC1 Induced Cell Apoptosis and Suppressed Cell Migration of Bladder Cancer

**FIGURE 5:**
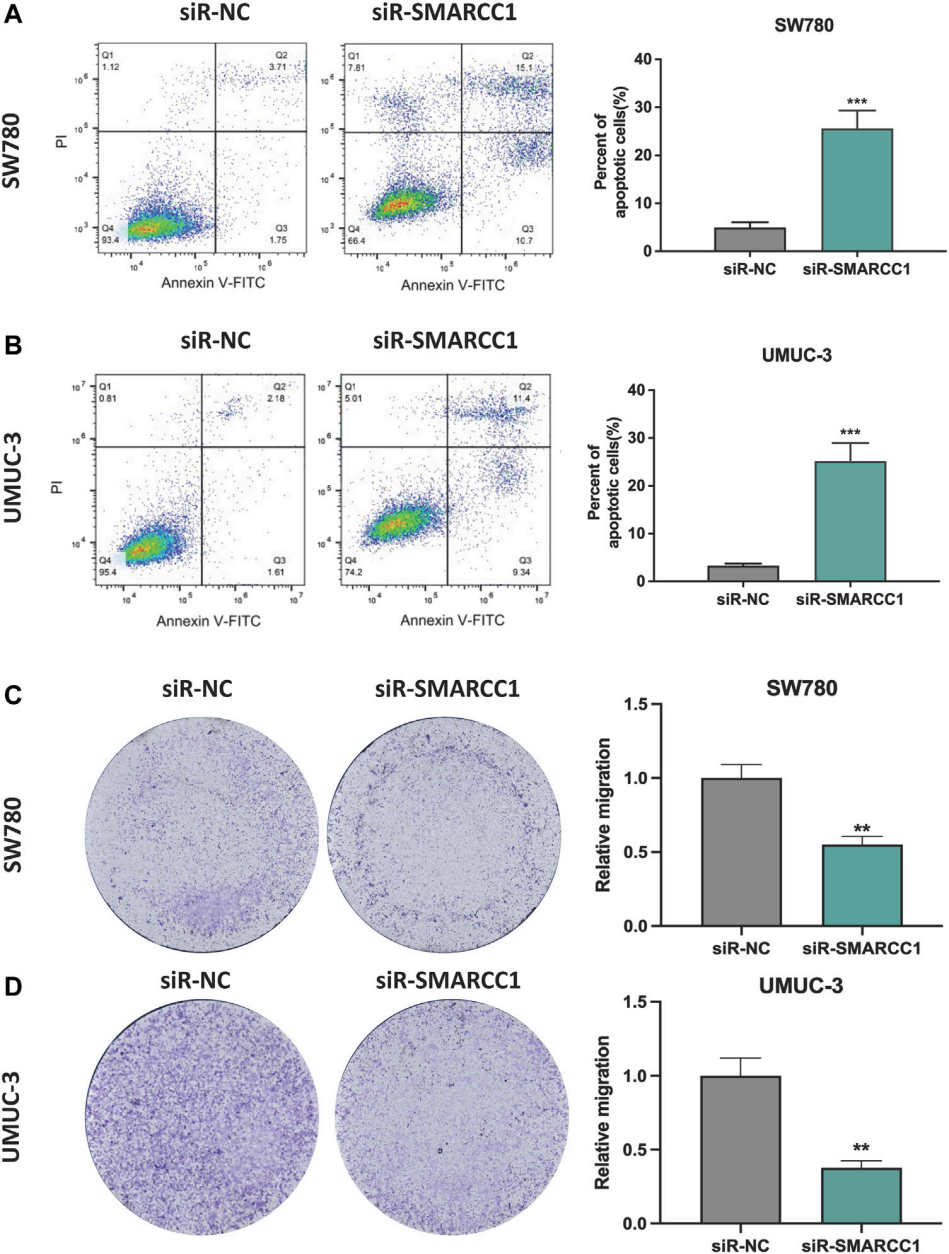
Silencing SMARCC1 induced cell apoptosis and suppressed cell migration of BC. **(A–B)** Flow cytometry analysis was performed to determine the apoptosis rate of cells. Knockdown of SMARCC1 expression markedly increased the number of apoptotic cells in both SW780 and UMUC-3 cell lines. **(C–D)** The transwell assay results revealed that the migration ability of SW780 and UMUC-3 cells were significantly inhibited after silencing SMARCC1. * *p* < 0.05, ** *p* < 0.01, *** *p* < 0.001.

In this study, we performed flow cytometry and transwell assay to observe the role of SMARCC1 in cell apoptosis and migration of BC. The results showed that there was a higher percentage of apoptotic cells in the siR-SMARCC1-transfected BC cell population than that in the siR-NC-transfected BC cell population, which indicated SMARCC1 silencing induced cell apoptosis (Figures 5A,B). In addition, transwell assay revealed that downregulation of SMARCC1 caused significant inhibition of cell migration in both SW780 and UMUC-3 cell lines (Figures 5C,D).

### 3.6 Downregulation of SMARCC1 Abrogated Oncogenic Potential in Bladder Cancer *in Vivo*

**FIGURE 6:**
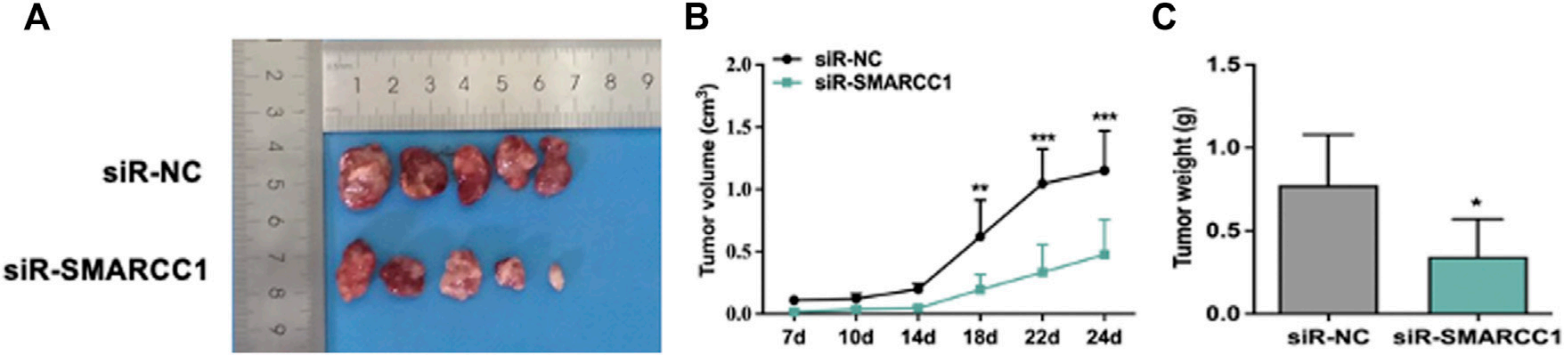
SMARCC1 promoted tumor growth of BC. UMUC-3 cells infected with siR-NC or siR-SMARCC1 were subcutaneously injected into nude mice. The tumors were harvested and photographed **(A)** on day 24. Data for tumor volume **(B)** and tumor weight **(C)** were shown as the mean ± SD (*n* = 5). Each siR-SMARCC1 group was compared with siR-NC group. * *p* < 0.05, ** *p* < 0.01, *** *p* < 0.001.

The effects of SMARCC1 knockdown on tumorigenicity were examined in a nude mouse model. Tumorigenesis was monitored in the siR-NC and siR-SMARCC1 groups. We generated a growth curve to measure tumor growth. The growth curve in Figure 6B showed that there was no significant difference in tumor volume between the two groups within the first 7–14 days. After 14 days, tumor growth was significantly accelerated in the siR-NC group and the difference of two groups in tumor volume was statistically significant (Figures 6A,B). The average tumor mass in the two groups were as follows: siR-NC vs. siR-SMARCC1, 0.774 ± 0.273 g vs. 0.346 ± 0.200 g (Figure 6C; *p* < 0.05). These data provided further evidence that down-regulation of SMARCC1 abrogated its oncogenic potential in BC *in vivo*.

## 4 Discussion

Bladder cancer, one of the most common malignant tumors of the urinary system, requires intense treatment and monitoring due to the high risk of recurrence and metastasis and may negatively affect health‐related quality of life (Smith et al., 2018). Thus, it is necessary to search for biomarkers of BC and further explore the mechanism of its carcinogenesis. Here, we proved that SMARCC1 enters the nucleus via KPNA2 and plays an oncogenic role in BC.

As previously mentioned, the IHC results showed that SMARCC1 was localized mainly in the nucleus. Protein complexes of the SWI/SNF family, which contain SMARCC1, remodel the nucleosome structure in an ATP-dependent manner (Phelan et al., 1999). It has been suggested that SMARCC1 can be localized in the nucleus and act as a transcription factor to regulate gene transcription. However, its specific mode of entry into the nucleus has not been researched. Small molecules with molecular weights of less than 40–50 kD pass freely through the nuclear membrane via diffusion, while biological macromolecules whose molecular weight exceeds this range or whose diameter is larger than 6 nm are actively transported in an energy-dependent manner mediated by soluble transport receptor proteins (Kurz et al., 1997). The classical nuclear protein import pathway is regulated by heterodimers composed of importin α/β and Karyopherin. Importin α/β can identify proteins containing a classical nuclear localization signal (cNLS), bind to karyopherin, and then enter the nucleus through the nuclear pore complex (NPC). Finally, nuclear RanGTP dissociates the import complex, and the importins are recycled (Goldfarb et al., 2004). KPNA2, one of the most important members of the Karyopherin α nuclear transporter family (Kelley et al., 2010), has been proven to be involved in the occurrence and development of tumors by regulating the nuclear translocation of tumor-related proteins (Sandrock et al., 2010; Wang et al., 2012; Li et al., 2013). The results of our Co-IP assay revealed that SMARCC1 interacts with KPNA2 under physiological conditions. We also found that the amount of SMARCC1 in the nucleus was significantly decreased when KPNA2 was silenced. Many nuclear-targeted proteins are transported through the NPC. Nup50, a structural component of the NPC, enhances the nucleocytoplasmic transport of proteins with a nuclear localization sequence by binding to import cargo-carrier complexes (Moore 2003). Nup153 provides a scaffold for Nup50, which contributes to the nuclear pore localization of Nup50 and the interaction between Nup50 and importin α, as well as other soluble factors involved in transport (Makise et al., 2012). Hence, we observed that SMARCC1 was also significantly decreased in the nuclear fraction when Nup50 and Nup153 were silenced. These results suggest that SMARCC1 promotes the occurrence and development of BC partially through the process of nucleocytoplasmic transport mediated by KPNA2, Nup50 and Nup153.

SMARCC1, also named BAF155, exists in a specific SWI/SNF complex that is present in almost all identified tissue and cell types and participates in the dynamic regulation of chromatin structure, gene transcription, cell cycle progression, the DNA damage response and other basic cellular processes (Yan et al., 2017). Here, we found that SMARCC1 participated in the occurrence and development of BC and affected the prognosis of patients with BC in a manner related to its aberrant expression in BC. In our research, we used RT-qPCR and western blot analyses to confirm that SMARCC1 was upregulated at both the mRNA and protein levels in 30 BC tissues and in BC cells compared with the corresponding normal tissues and cells. This discovery was consistent with the observed overexpression of SMARCC1 in several tumors (Heebøll et al., 2008; Shadeo et al., 2008; Wang et al., 2014; Ke et al., 2018). These findings suggest that high expression of SMARCC1 is closely related to BC carcinogenesis and development. In addition, the relationships between the protein level of SMARCC1 and clinical parameters were evaluated by IHC staining in 54 samples of BC sections. The results showed that high expression of SMARCC1 was positively correlated with the T stage of BC but was not related to the tumor grade, tumor volume or lymph node metastasis status. A Kaplan‐Meier survival analysis demonstrated that high expression of SMARCC1 in patients with BC resulted in decreased survival rates compared to those of patients negative for SMARCC1 expression. These results strongly suggest that SMARCC1 has clinical value as a novel diagnostic and prognostic marker for BC. Considering the high SMARCC1 expression in BC cell lines shown by RT-qPCR and western blot analyses, we knocked down the expression of SMARCC1 in the SW780 and UMUC-3 cell lines. We observed that SMARCC1 downregulation inhibited cell proliferation both *in vivo* and *in vitro* and also resulted in G1/S arrest and an increase in the number of apoptotic cells. Moreover, SMARCC1 silencing led to reduced cell migration in BC cell lines.

In summary, we proposed that KPNA2, Nup50 and Nup153 regulated the process of SMARCC1 nuclear translocation in BC. Our results revealed that SMARCC1 was significantly upregulated in BC. Positive SMARCC1 expression was associated with the T stage of BC patients. Silencing SMARCC1 expression in BC cells by specific siRNA significantly decreased the proliferative and migratory abilities and enhanced G1/S cell cycle arrest and apoptosis. Consequently, SMARCC1 may be a competent candidate as a diagnostic and therapeutic target for BC. Further studies are required to explore the mechanism and assess the role of SMARCC1 *in vivo*.

## Data Availability

The datasets presented in this study can be found in online repositories. The names of the repository/repositories and accession number(s) can be found in the article/Supplementary Material.
