# Supplementary material for: SMARCC1 Enters the Nucleus via KPNA2 and Plays an Oncogenic Role in Bladder Cancer
Source: Front Mol Biosci. 2022 May 20;9:902220. doi: 10.3389/fmolb.2022.902220 (PMC9163745; doi:10.3389/fmolb.2022.902220)
Supplement: Supplementary file 1 [file DataSheet1.ZIP › SMARCC1 RAW data/Figure 5/apoptosis raw data/UMUC-3/UMUC3 cell apoptosis all .pdf]

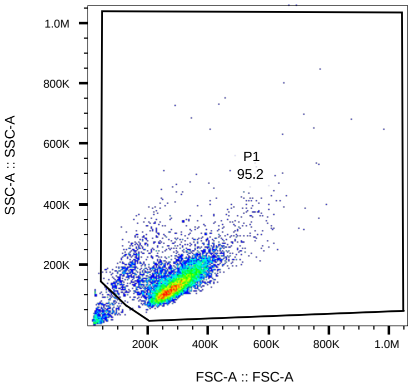

UMUC3 NC.fcs
Ungated
10541

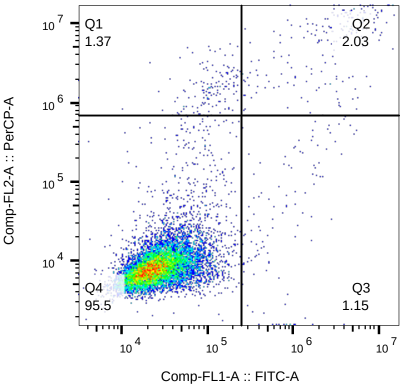

UMUC3 NC.fcs
P1
10036

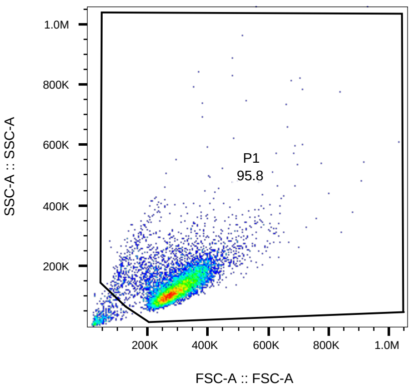

UMUC3 NC2.fcs
Ungated
10458

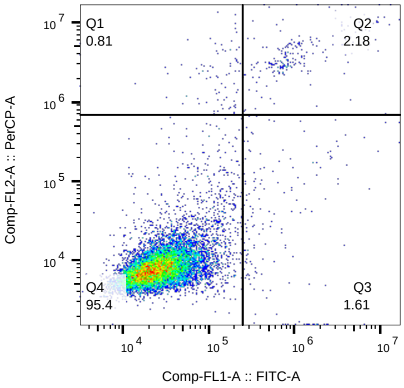

UMUC3 NC2.fcs
P1
10016

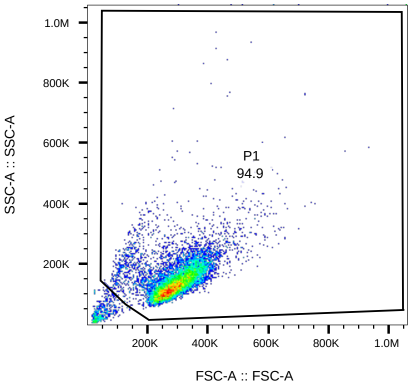

UMUC3 NC3.fcs
Ungated
10568

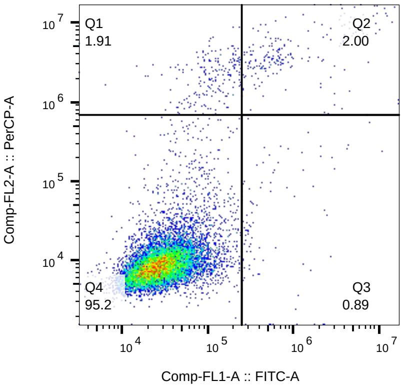

UMUC3 NC3.fcs
P1
10024

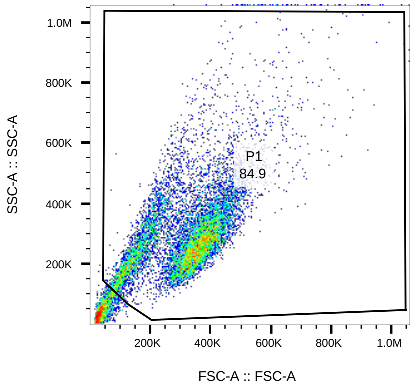

UMUC3 siR-SMARCC1-1.fcs
Ungated
11972

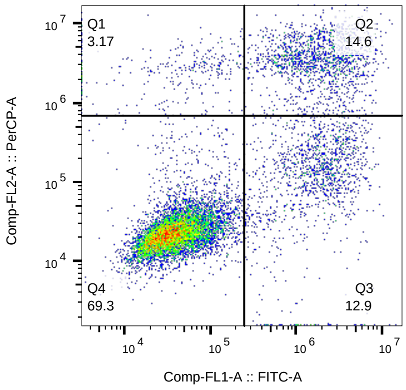

UMUC3 siR-SMARCC1-1.fcs
P1
10162

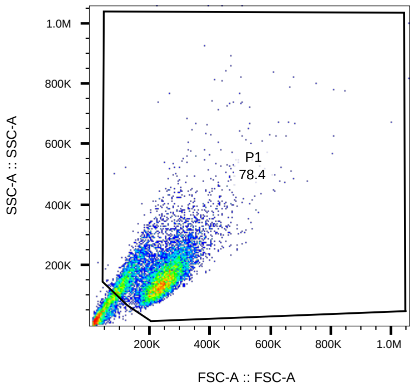

UMUC3 siR-SMARCC1-2.fcs
Ungated
12819

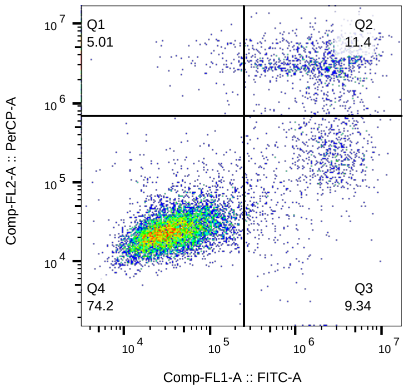

UMUC3 siR-SMARCC1-2.fcs
P1
10055

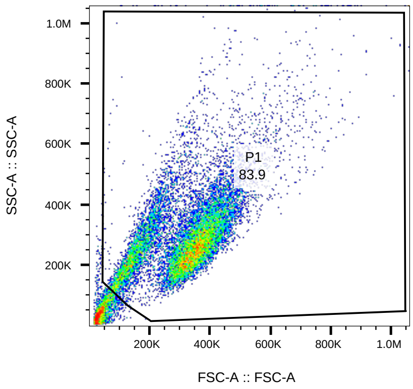

UMUC3 siR-SMARCC1-3.fcs
Ungated
15992

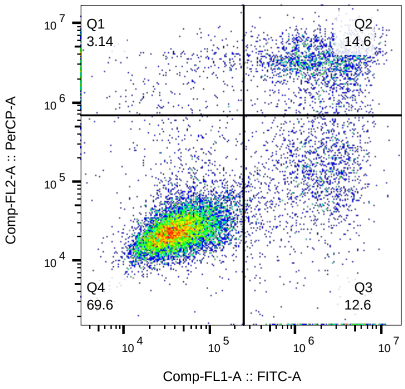

UMUC3 siR-SMARCC1-3.fcs
P1
13423

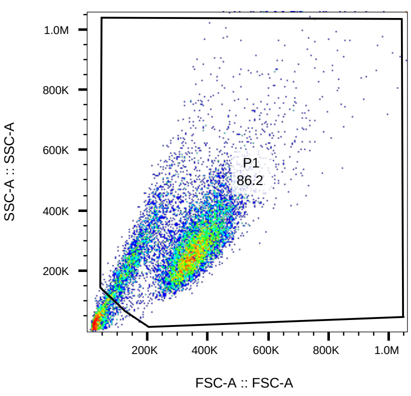

UMUC3 siR-SMARCC1-4.fcs
Ungated
11746

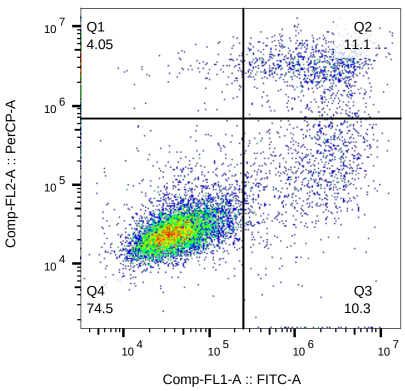

UMUC3 siR-SMARCC1-4.fcs
P1
10128
