# Supplementary material for: SMARCC1 Enters the Nucleus via KPNA2 and Plays an Oncogenic Role in Bladder Cancer
Source: Front Mol Biosci. 2022 May 20;9:902220. doi: 10.3389/fmolb.2022.902220 (PMC9163745; doi:10.3389/fmolb.2022.902220)
Supplement: Supplementary file 1 [file DataSheet1.ZIP › SMARCC1 RAW data/Figure 5/apoptosis raw data/SW780/sw780 app 21-Mar-2022-Layout.pdf]

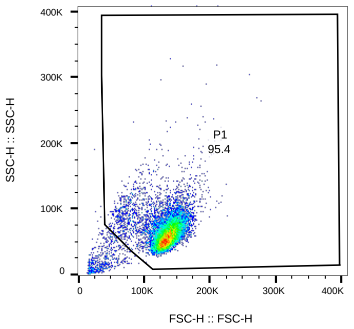

SW780 SIR-NC1.fcs
Ungated
10689

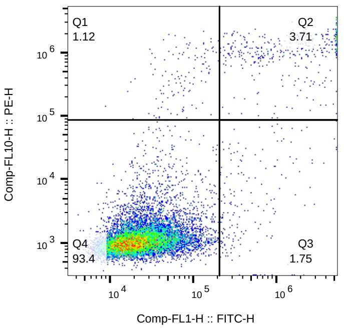

SW780 SIR-NC1.fcs
P1
10194

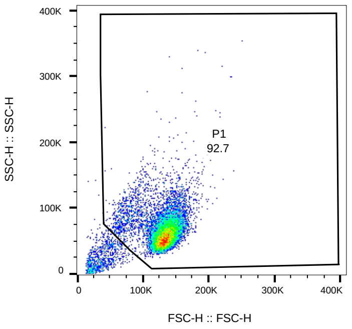

SW780 SIR-NC2.fcs
Ungated
10975

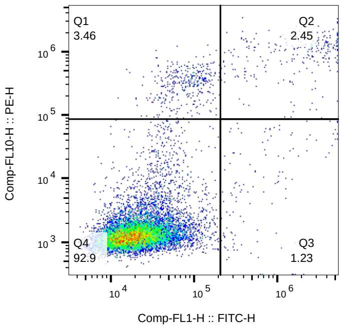

SW780 SIR-NC2.fcs
P1
10170

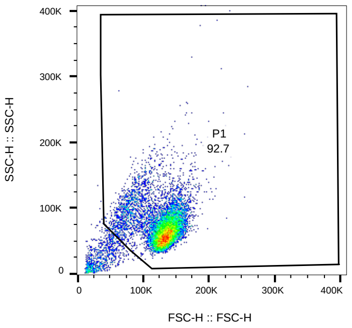

SW780 SIR-NC3.fcs
Ungated
11090

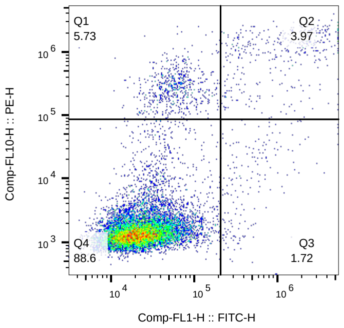

SW780 SIR-NC3.fcs
P1
10279

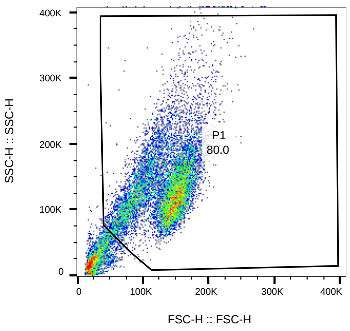

SW780 SIR-SMARCC1-1.fcs
Ungated
13492

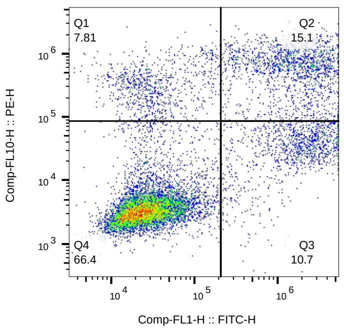

SW780 SIR-SMARCC1-1.fcs
P1
10796

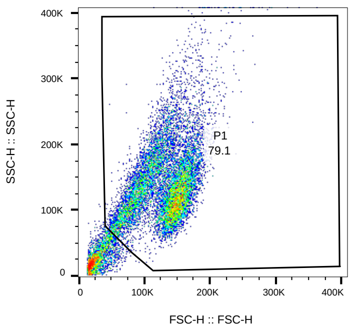

SW780 SIR-SMARCC1-2.fcs
Ungated
13270

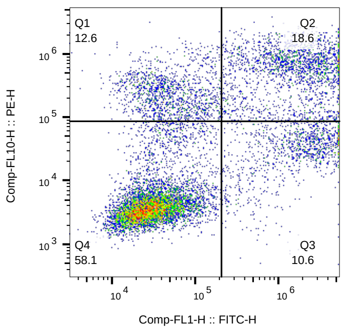

SW780 SIR-SMARCC1-2.fcs
P1
10502

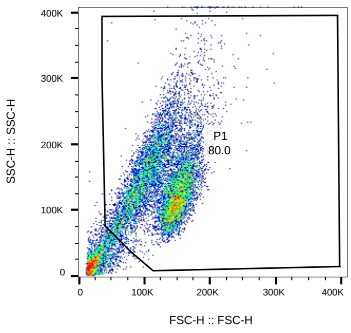

SW780 SIR-SMARCC1-3.fcs
Ungated
12973

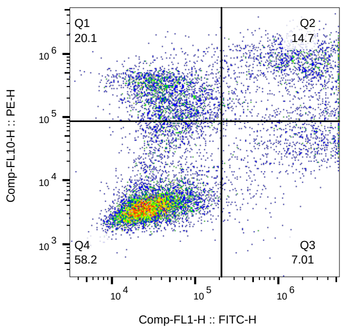

SW780 SIR-SMARCC1-3.fcs
P1
10374
