# Supplementary figures and images for: SMARCC1 Enters the Nucleus via KPNA2 and Plays an Oncogenic Role in Bladder Cancer
Source: Front Mol Biosci. 2022 May 20;9:902220. doi: 10.3389/fmolb.2022.902220 (PMC9163745; doi:10.3389/fmolb.2022.902220)

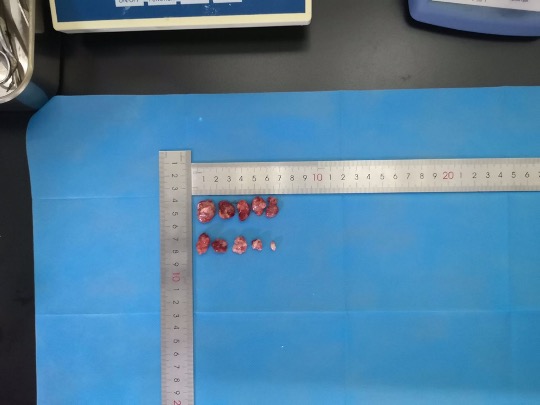

Supplement: Supplementary file 1 [file DataSheet1.ZIP › SMARCC1 RAW data/Figure 6/tumor.jpg]

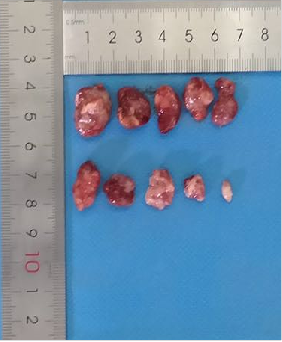

Supplement: Supplementary file 1 [file DataSheet1.ZIP › SMARCC1 RAW data/Figure 6/tumor.tif]

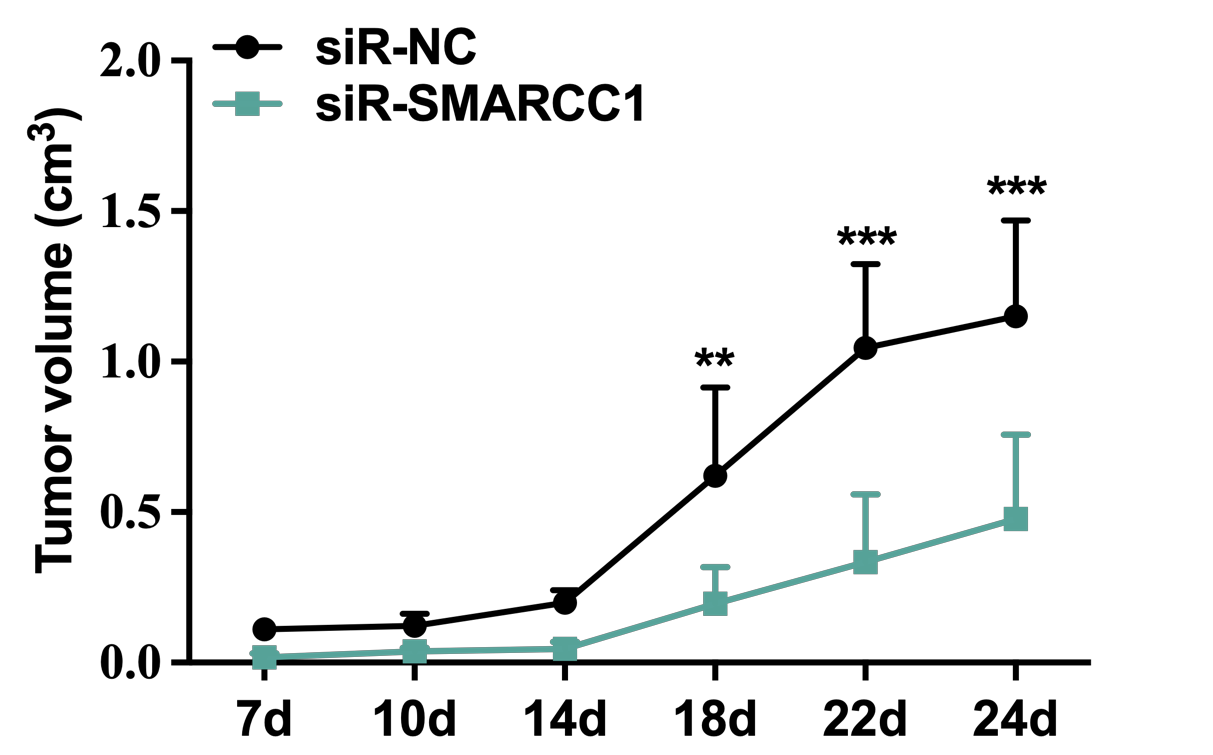

Supplement: Supplementary file 1 [file DataSheet1.ZIP › SMARCC1 RAW data/Figure 6/Tumor volume.tiff]

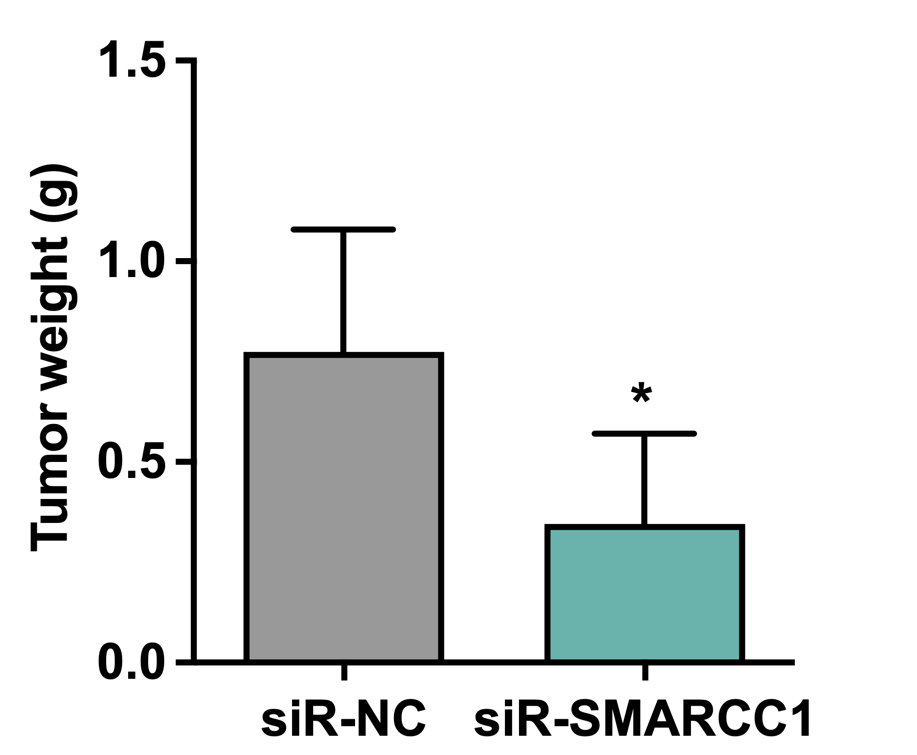

Supplement: Supplementary file 1 [file DataSheet1.ZIP › SMARCC1 RAW data/Figure 6/Tumor weight.tiff]

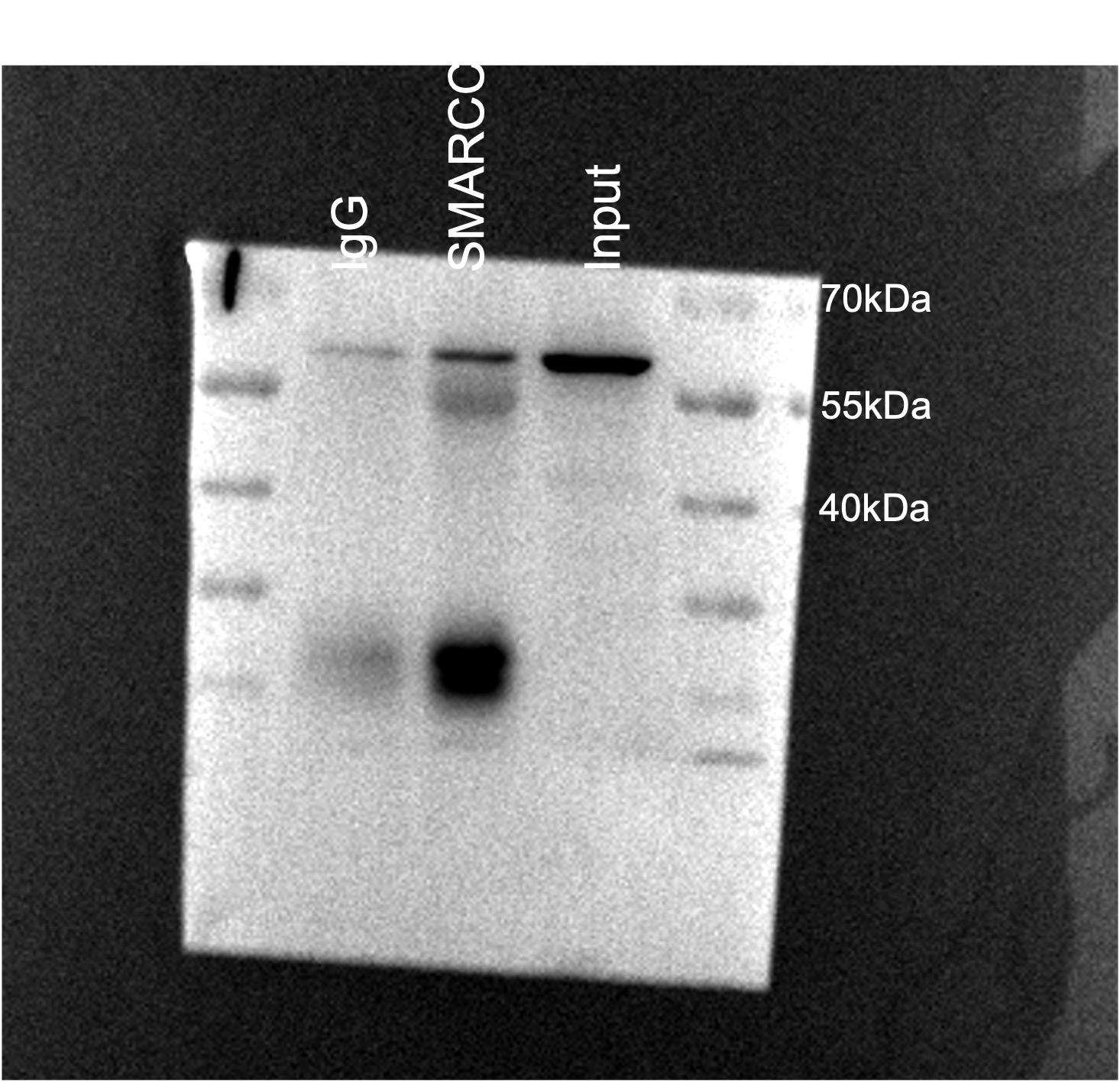

Supplement: Supplementary file 1 [file DataSheet1.ZIP › SMARCC1 RAW data/Figure 1/figure 1B KPNA2.jpg]

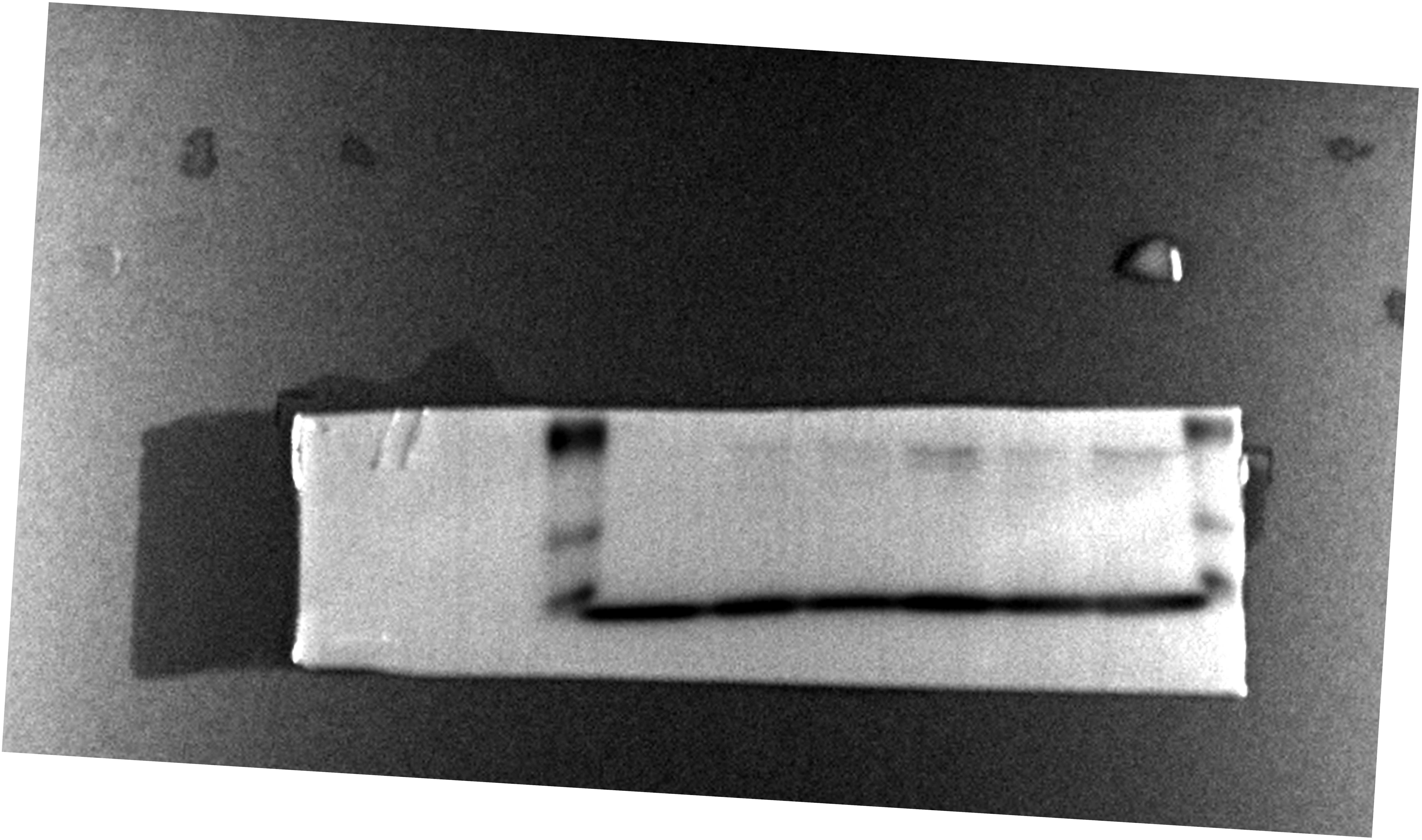

Supplement: Supplementary file 1 [file DataSheet1.ZIP › SMARCC1 RAW data/Figure 1/figure 1C H3.jpg]

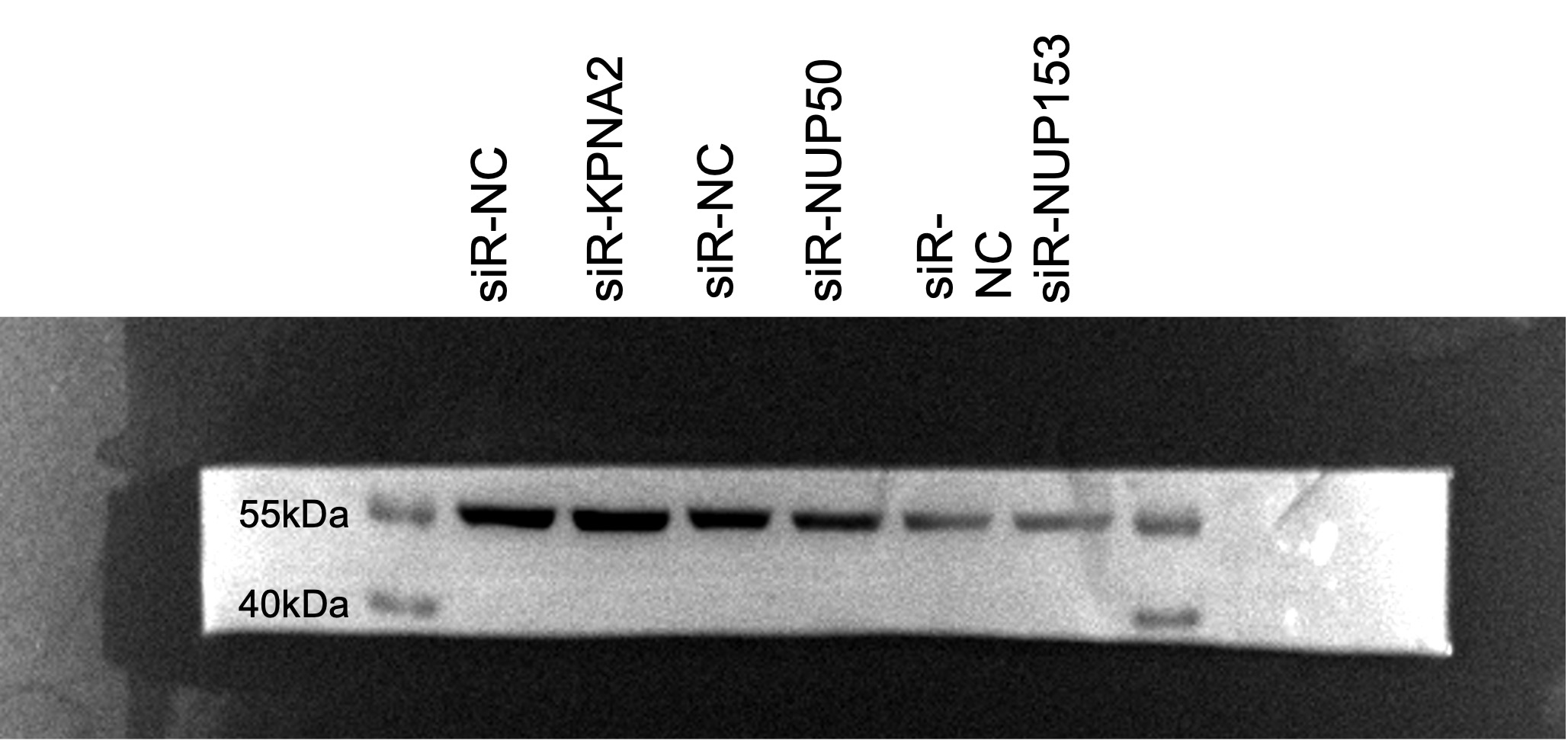

Supplement: Supplementary file 1 [file DataSheet1.ZIP › SMARCC1 RAW data/Figure 1/figure 1D b-tubulin.jpg]

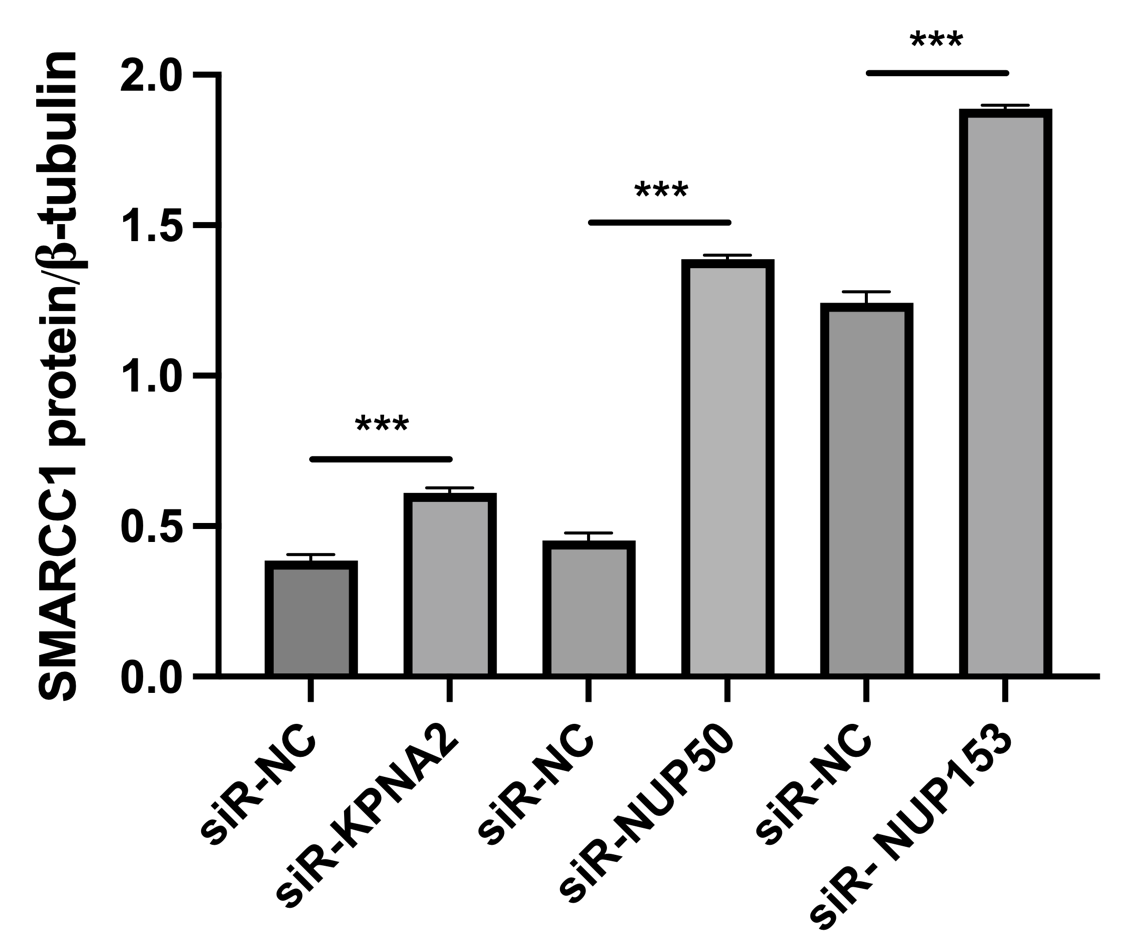

Supplement: Supplementary file 1 [file DataSheet1.ZIP › SMARCC1 RAW data/Figure 1/Gray analysis of figure 1D.tiff]

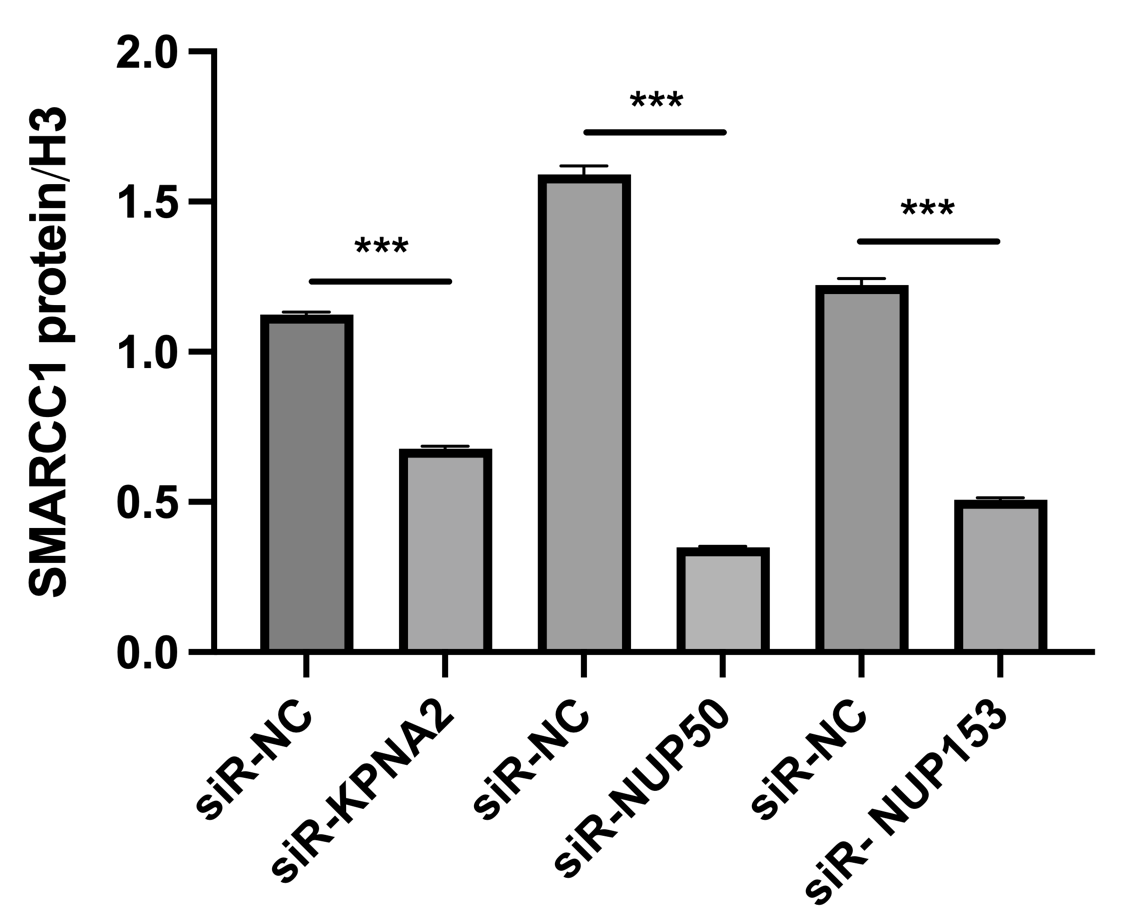

Supplement: Supplementary file 1 [file DataSheet1.ZIP › SMARCC1 RAW data/Figure 1/Gray analysis of figure 1C.tiff]

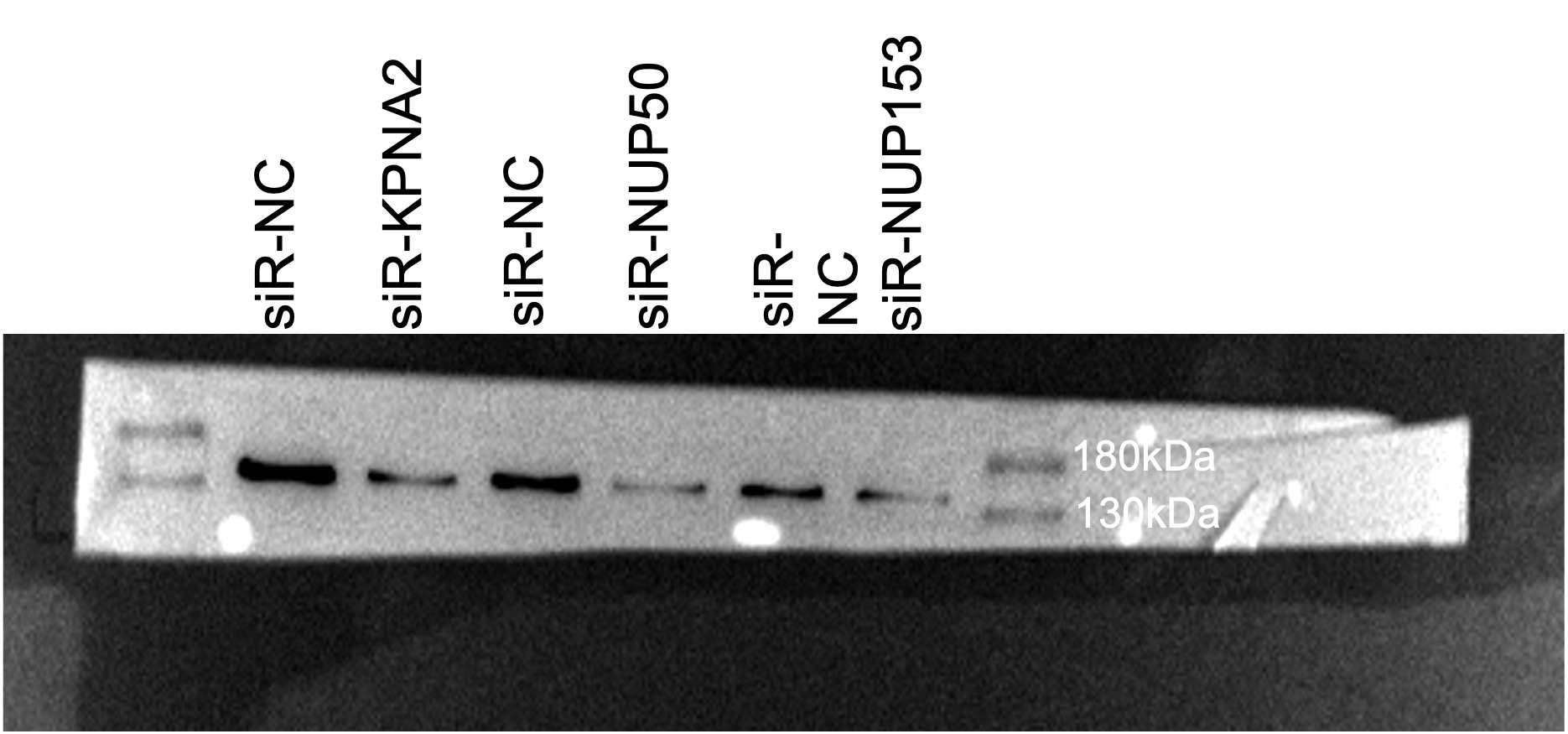

Supplement: Supplementary file 1 [file DataSheet1.ZIP › SMARCC1 RAW data/Figure 1/figure 1C SMARCC1.jpg]

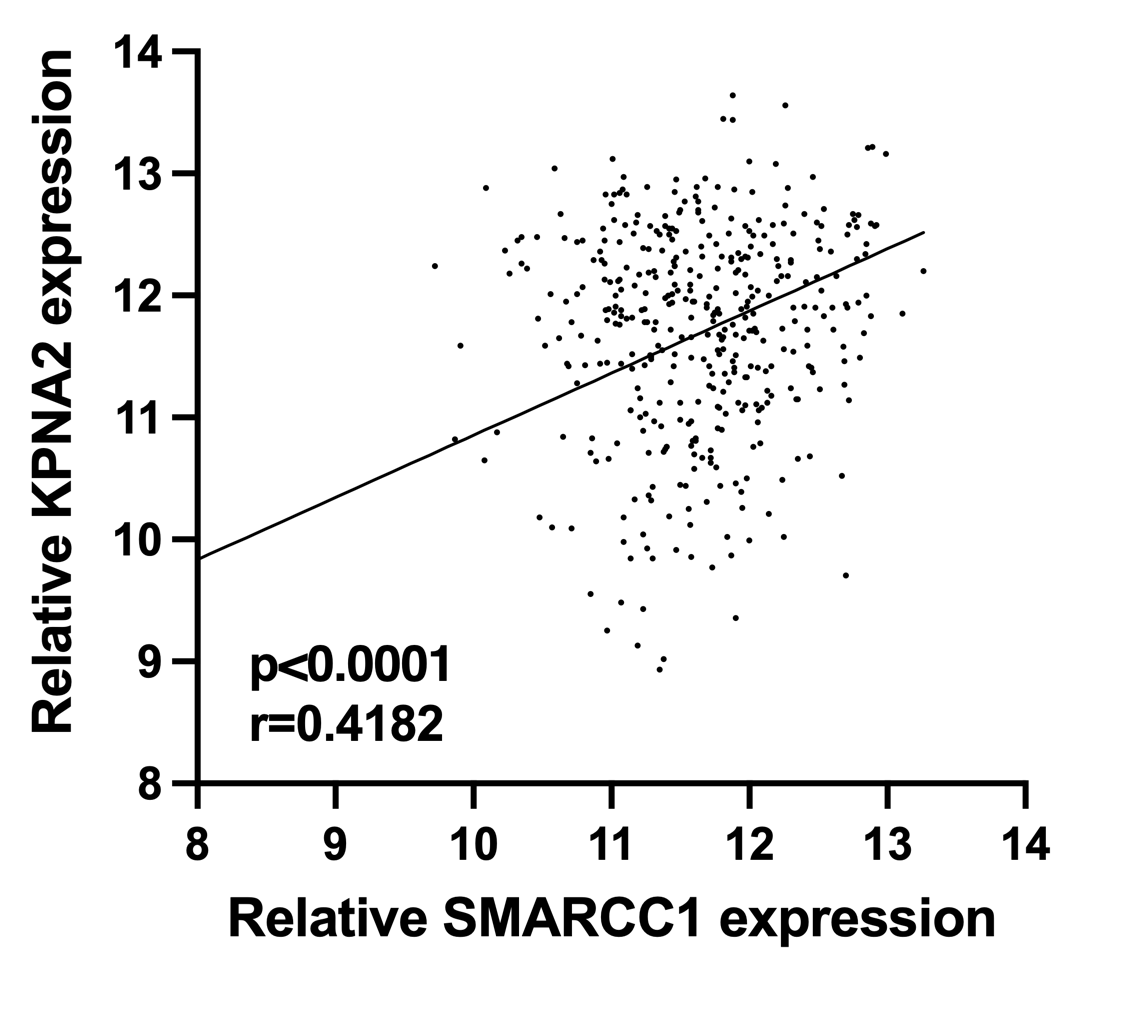

Supplement: Supplementary file 1 [file DataSheet1.ZIP › SMARCC1 RAW data/Figure 1/the correlation between SMARCC1 and KPNA2.tiff]

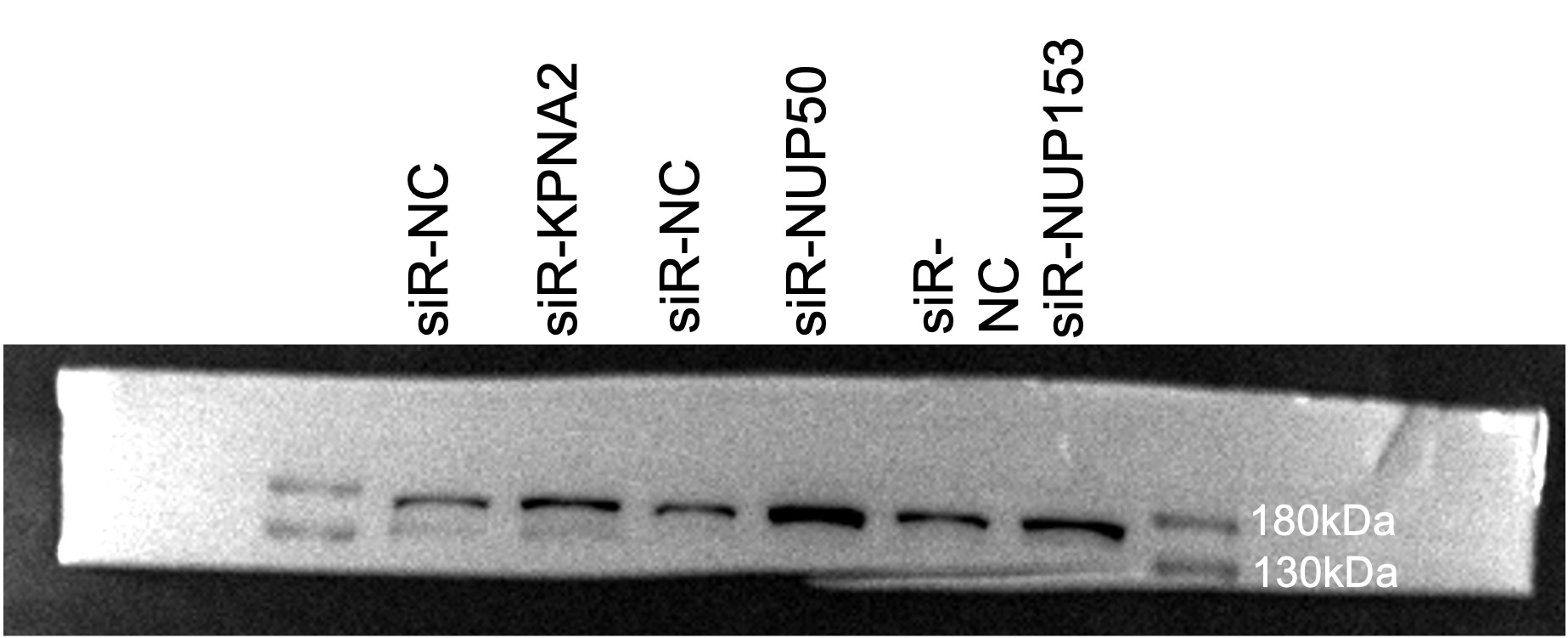

Supplement: Supplementary file 1 [file DataSheet1.ZIP › SMARCC1 RAW data/Figure 1/figure 1D SMARCC1.jpg]

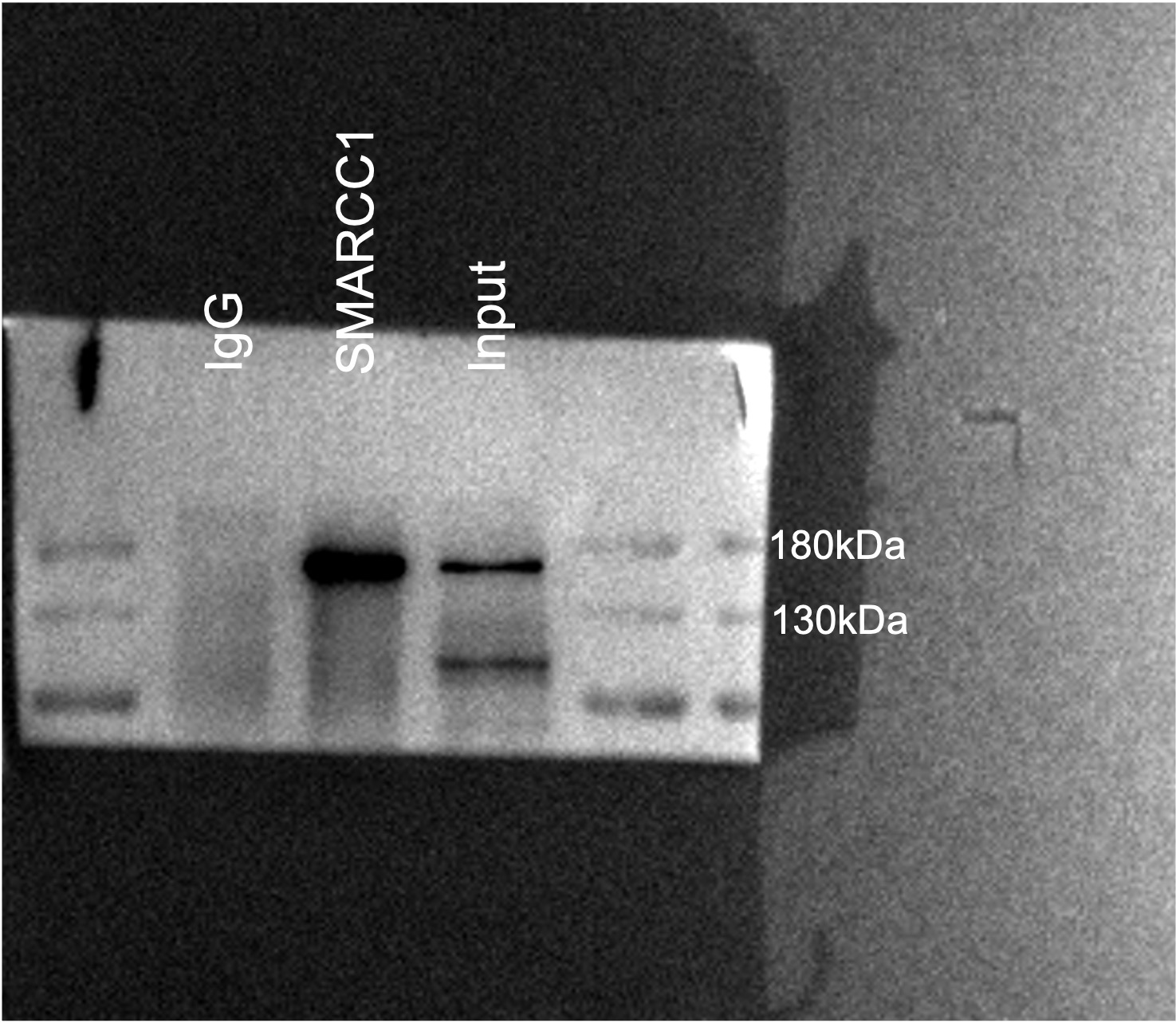

Supplement: Supplementary file 1 [file DataSheet1.ZIP › SMARCC1 RAW data/Figure 1/figure 1B SMARCC1.jpg]

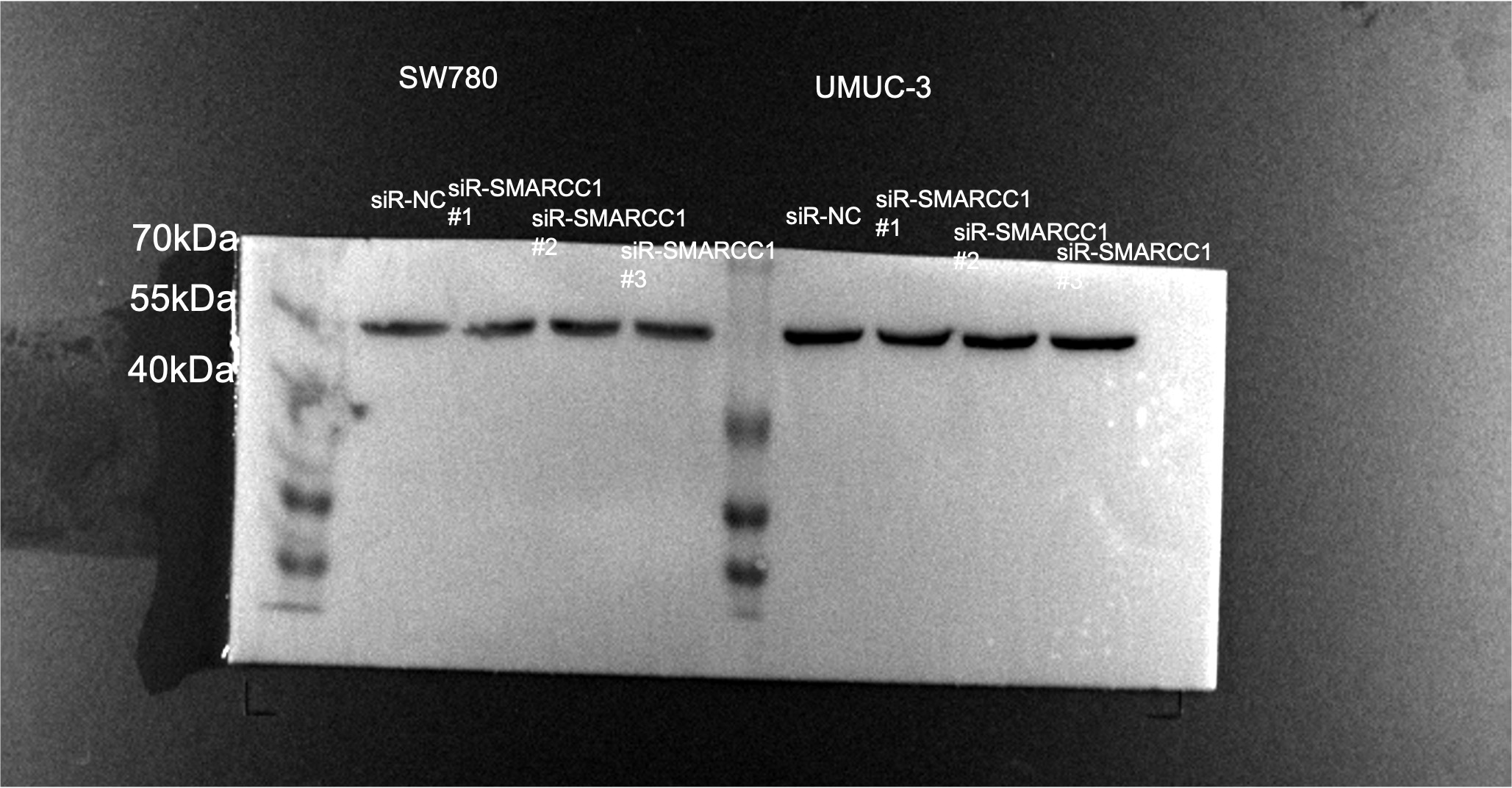

Supplement: Supplementary file 1 [file DataSheet1.ZIP › SMARCC1 RAW data/Supplementary data/b-tubulin siR-SMARCC1.jpg]

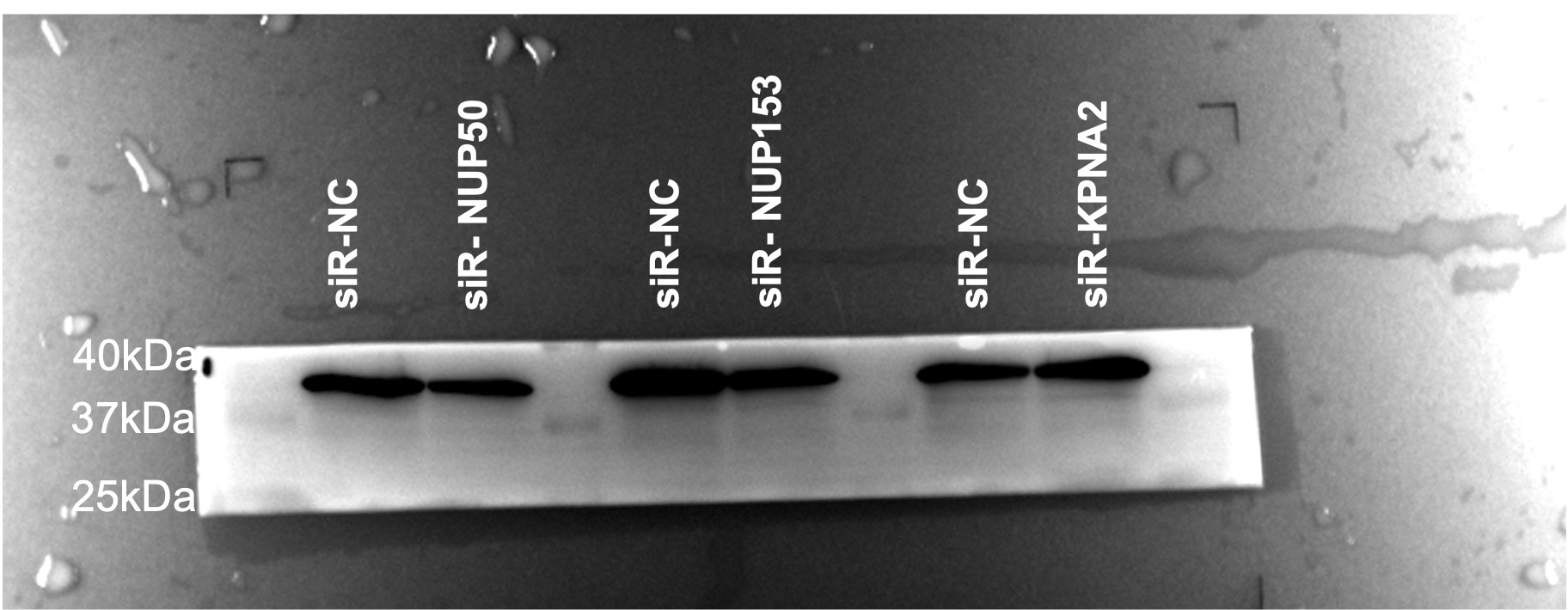

Supplement: Supplementary file 1 [file DataSheet1.ZIP › SMARCC1 RAW data/Supplementary data/GAPDH siRNA of NUP50 NUP153 KPNA2.jpg]

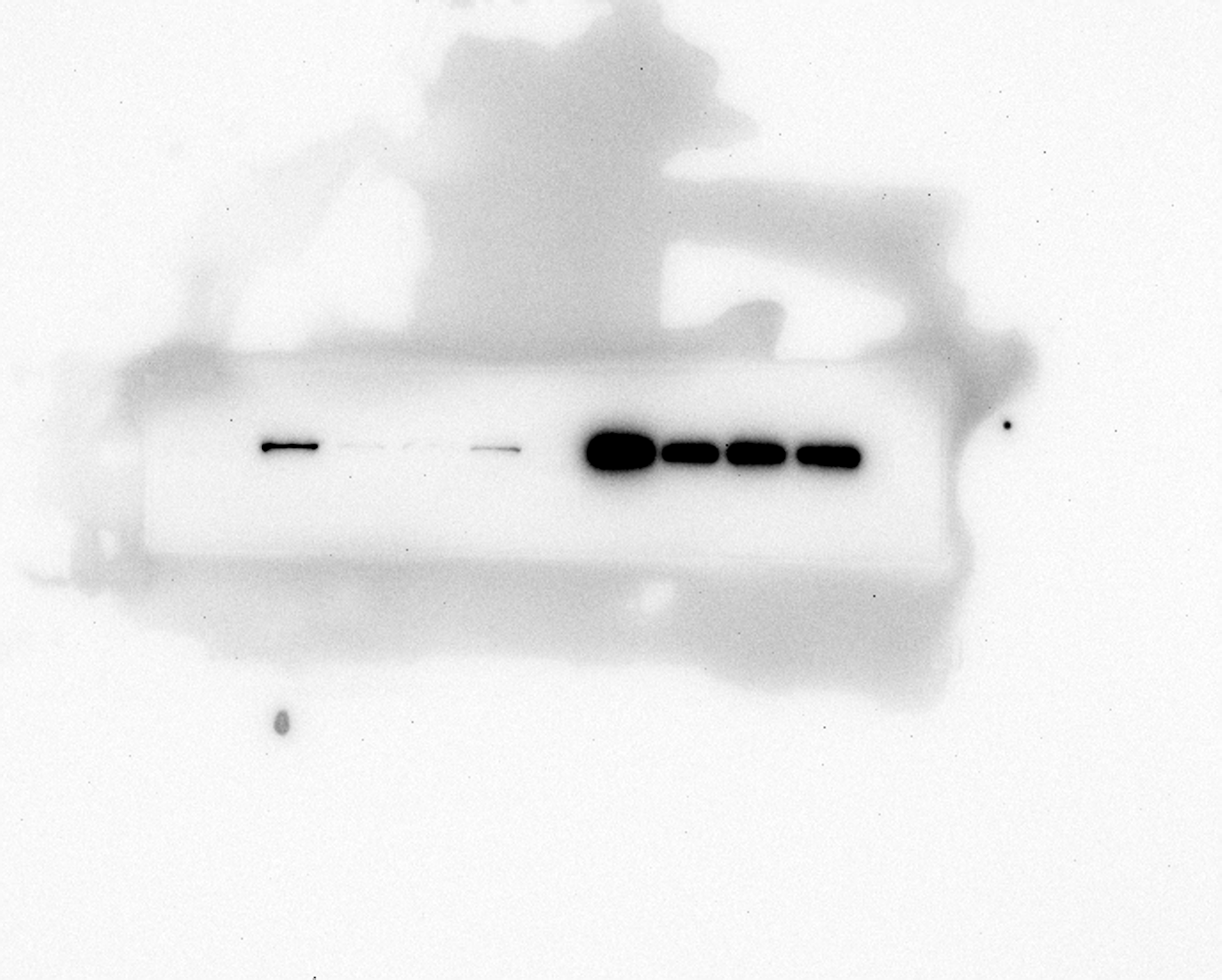

Supplement: Supplementary file 1 [file DataSheet1.ZIP › SMARCC1 RAW data/Supplementary data/SMARCC1 siR-SMARCC1.Tif]

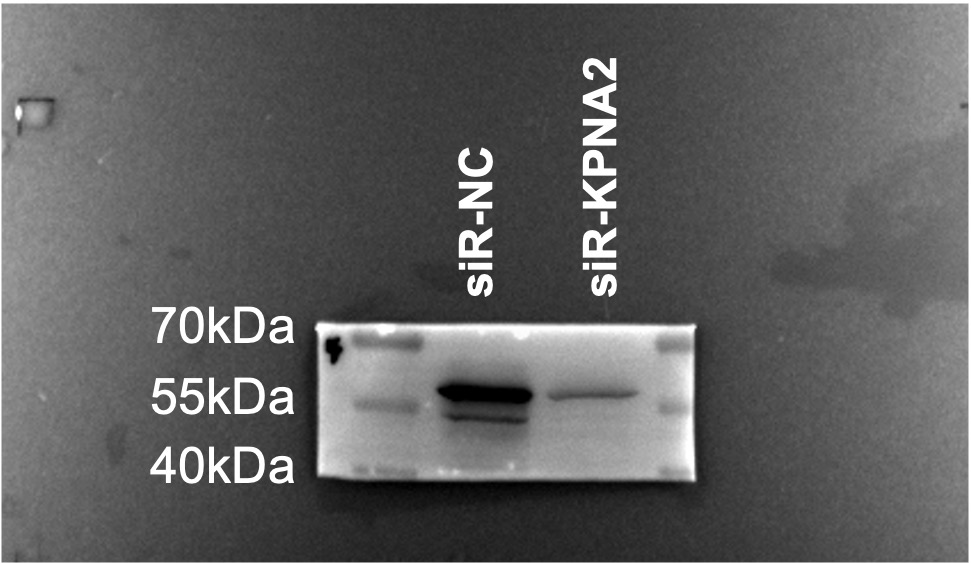

Supplement: Supplementary file 1 [file DataSheet1.ZIP › SMARCC1 RAW data/Supplementary data/KPNA2 siRNA.jpg]

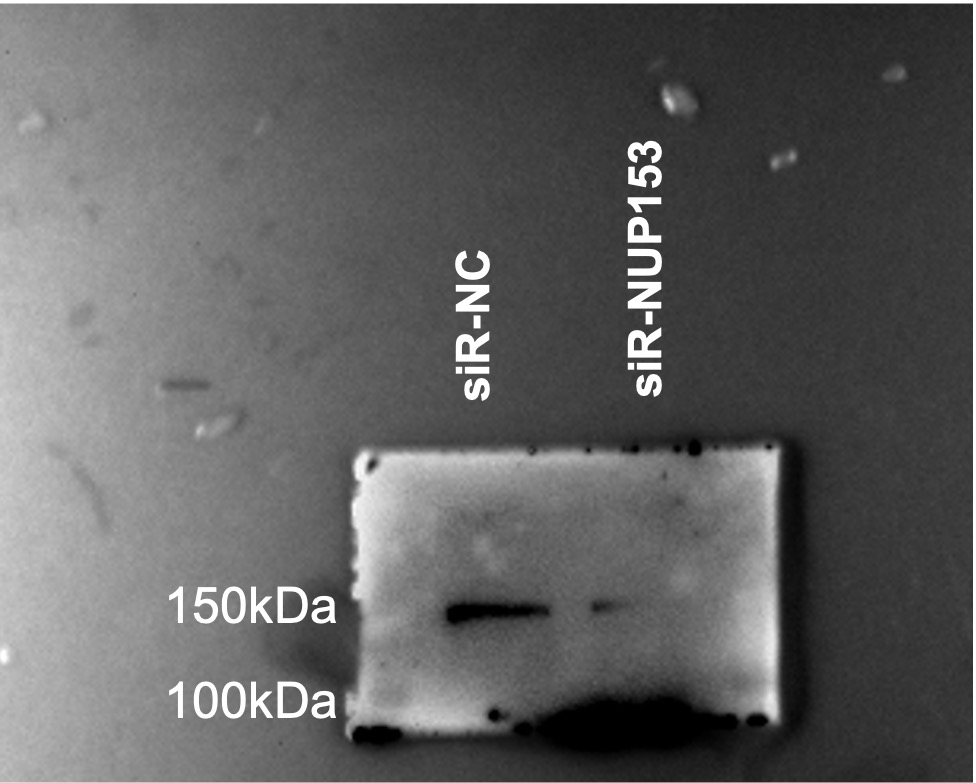

Supplement: Supplementary file 1 [file DataSheet1.ZIP › SMARCC1 RAW data/Supplementary data/NUP153 siRNA.jpg]

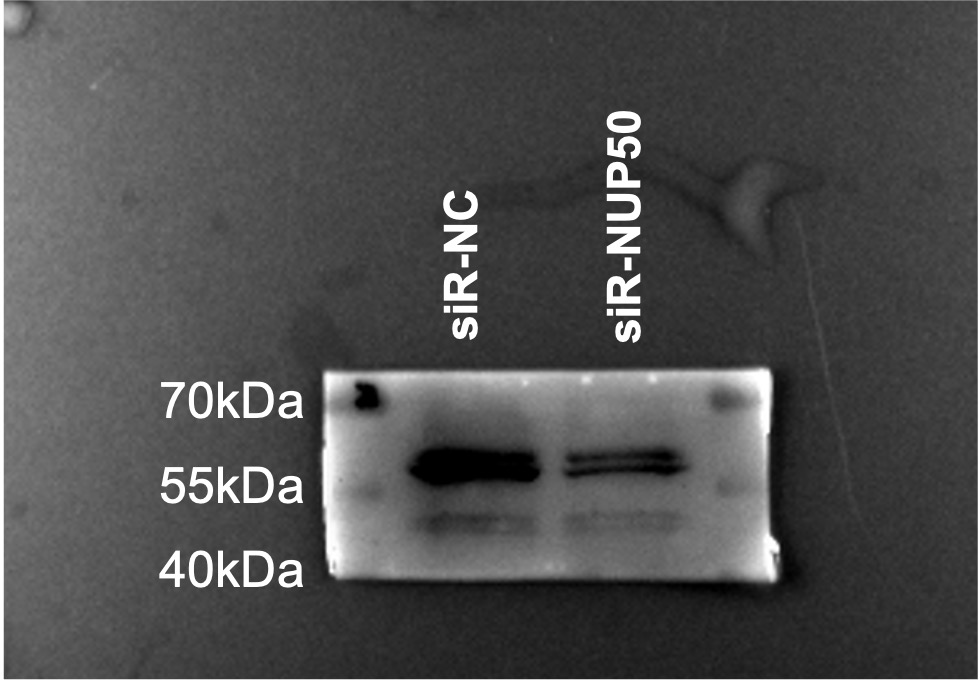

Supplement: Supplementary file 1 [file DataSheet1.ZIP › SMARCC1 RAW data/Supplementary data/NUP50 siRNA.jpg]

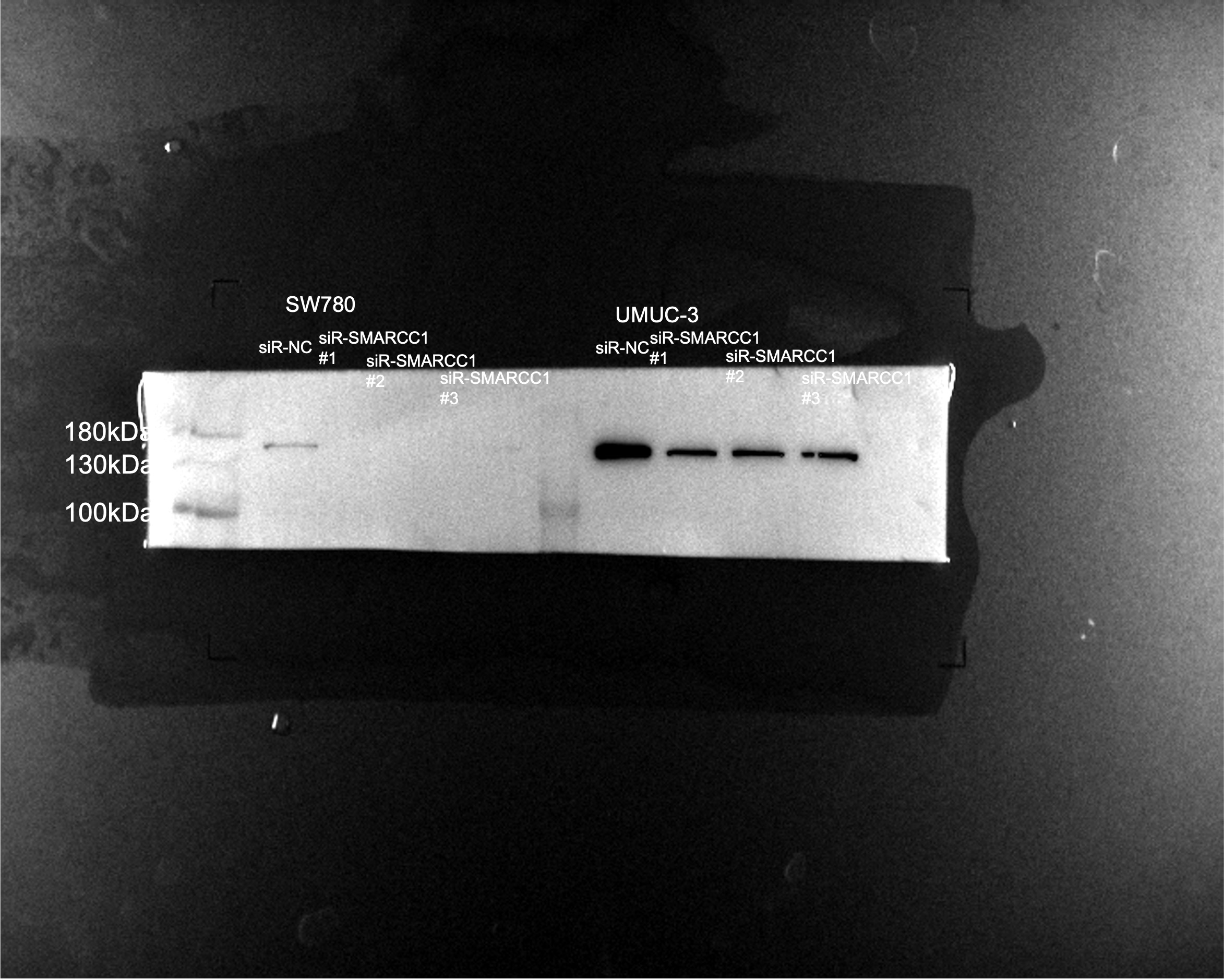

Supplement: Supplementary file 1 [file DataSheet1.ZIP › SMARCC1 RAW data/Supplementary data/SMARCC1 siR-SMARCC1.jpg]

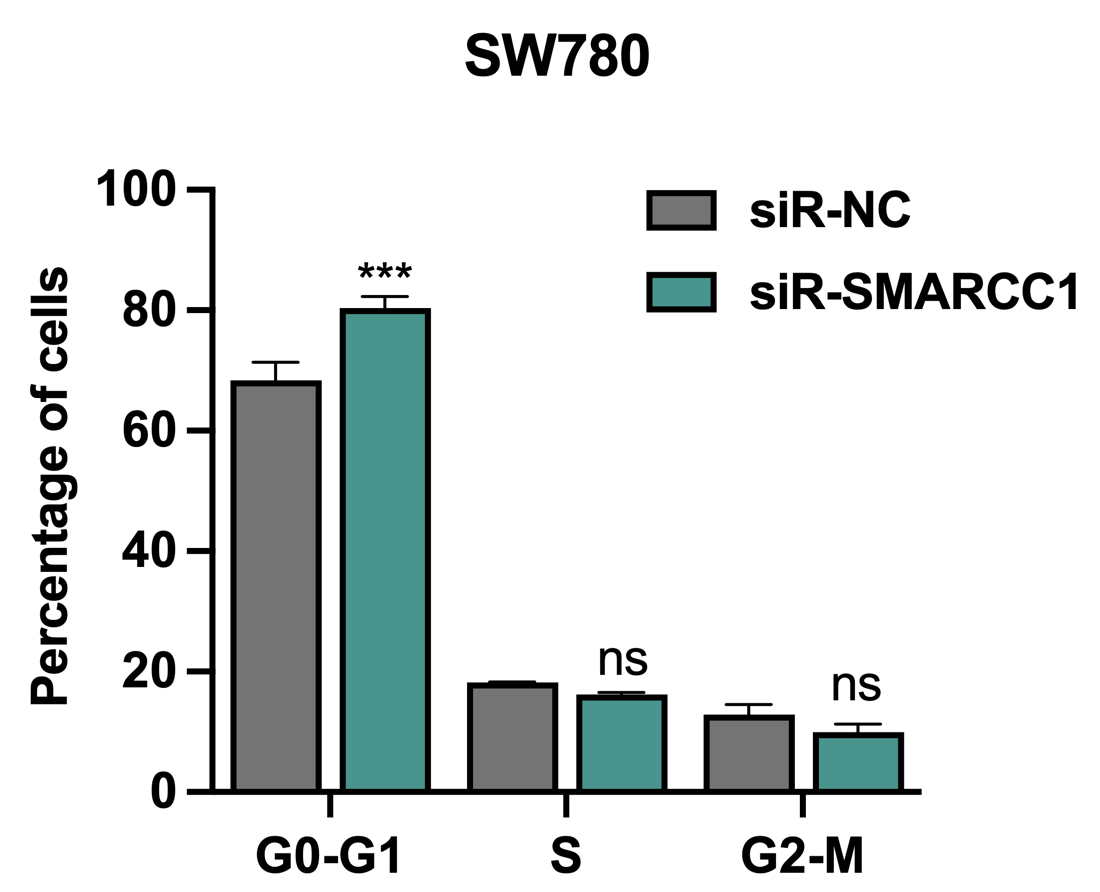

Supplement: Supplementary file 1 [file DataSheet1.ZIP › SMARCC1 RAW data/Figure 4/SW780 Cell Cycle.tiff]

# SW780

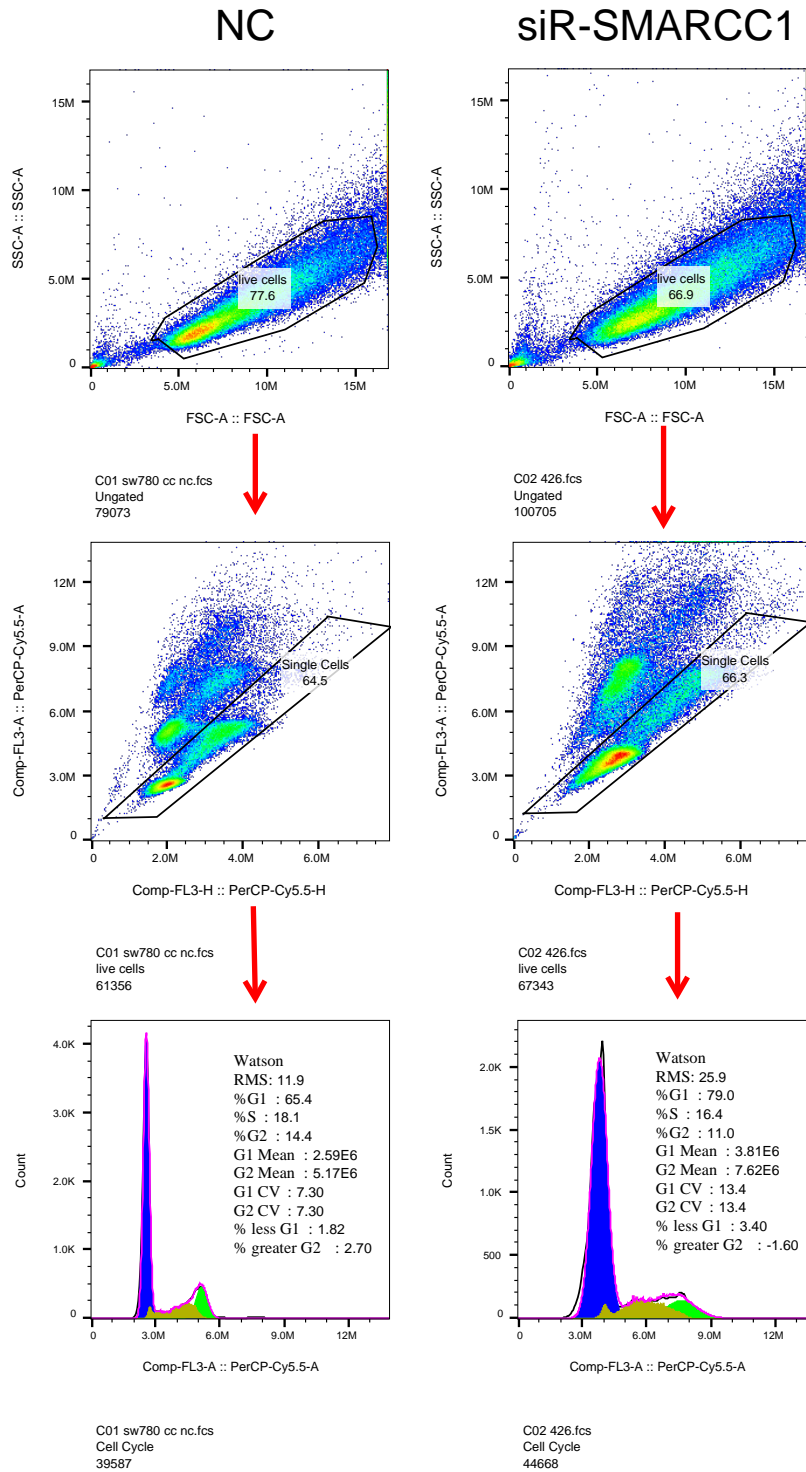

Gating for Figure 4E: Gates are shown sequentially from up to down.

Supplement: Supplementary file 1 [file DataSheet1.ZIP › SMARCC1 RAW data/Figure 4/SW780 cell cycle.pdf]

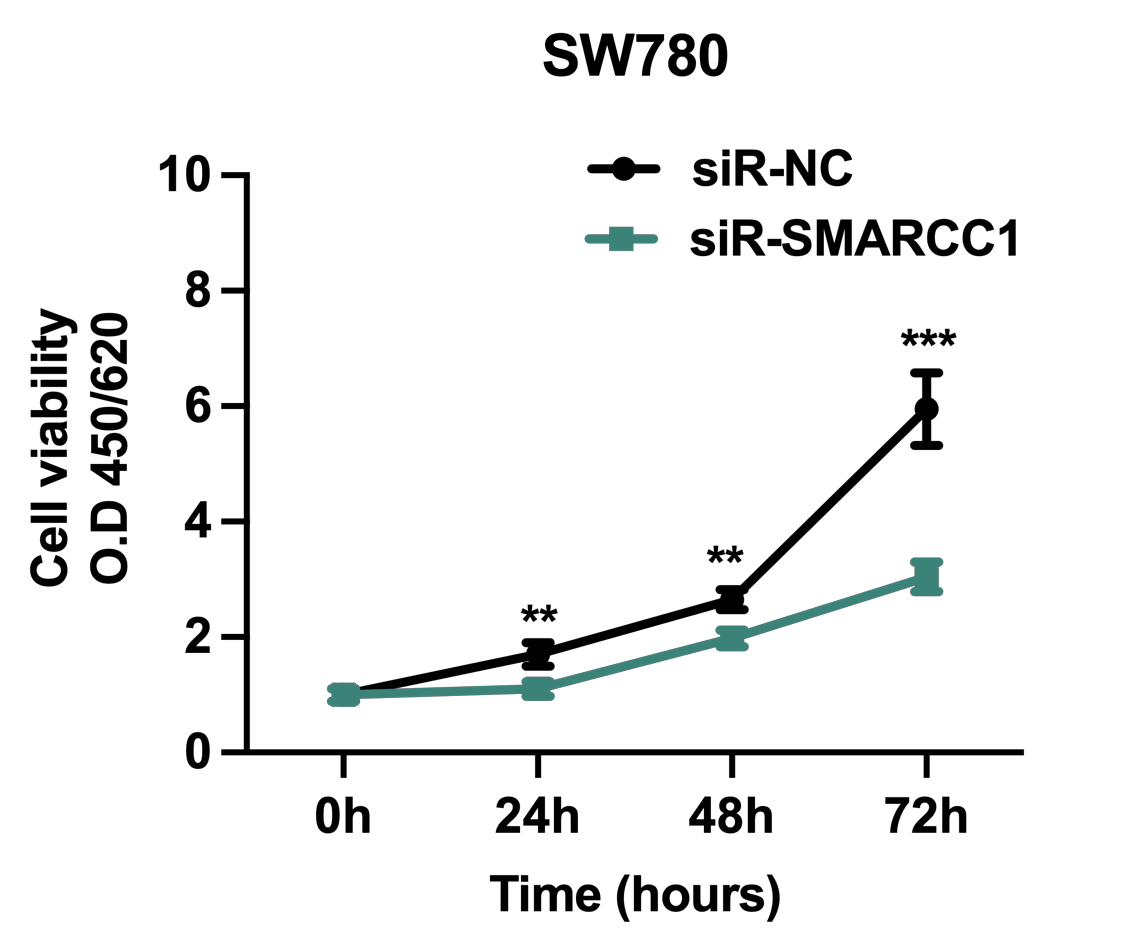

Supplement: Supplementary file 1 [file DataSheet1.ZIP › SMARCC1 RAW data/Figure 4/SW780 cck8.tiff]

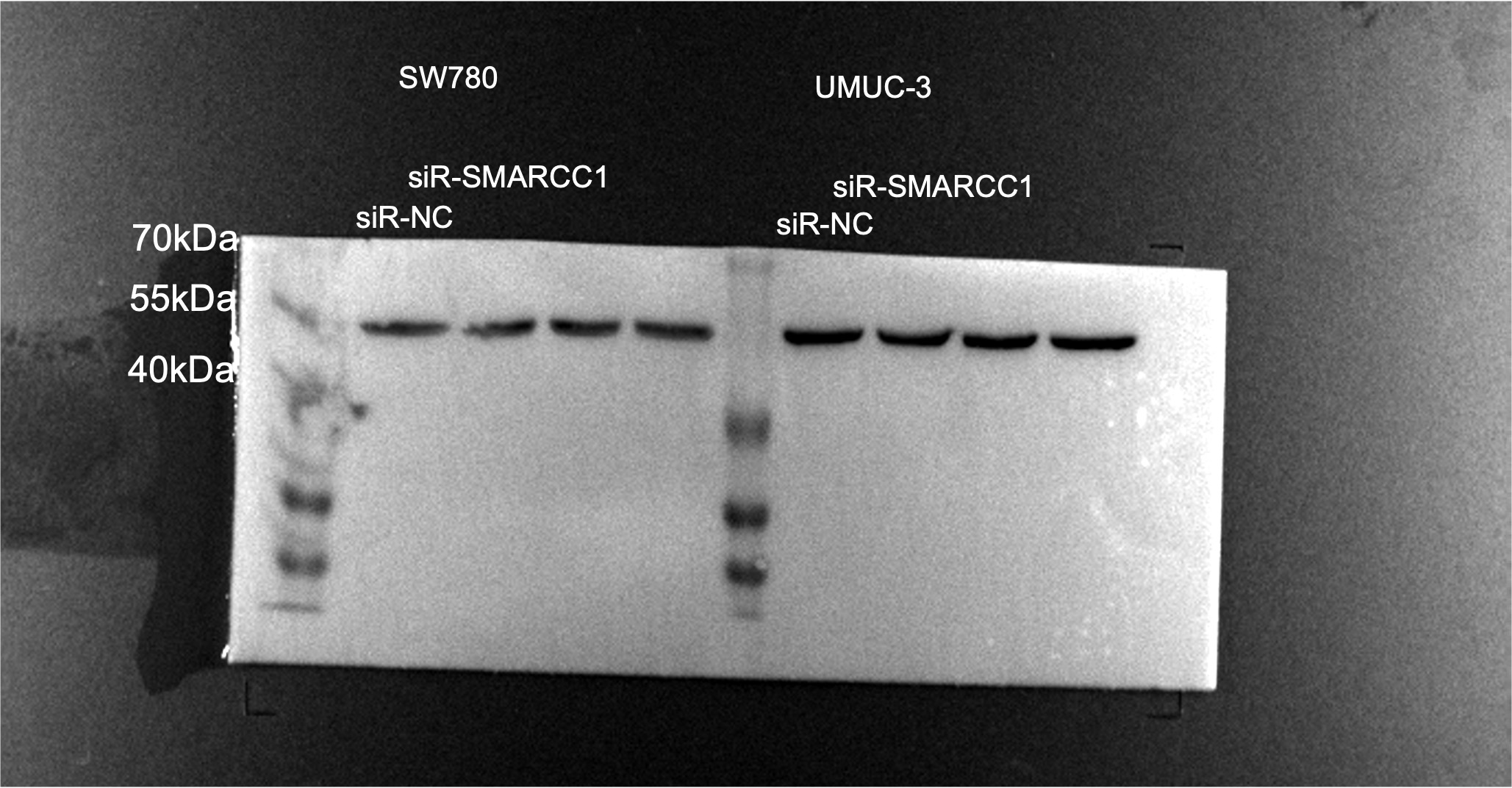

Supplement: Supplementary file 1 [file DataSheet1.ZIP › SMARCC1 RAW data/Figure 4/figure 4 b-tubulin.jpg]

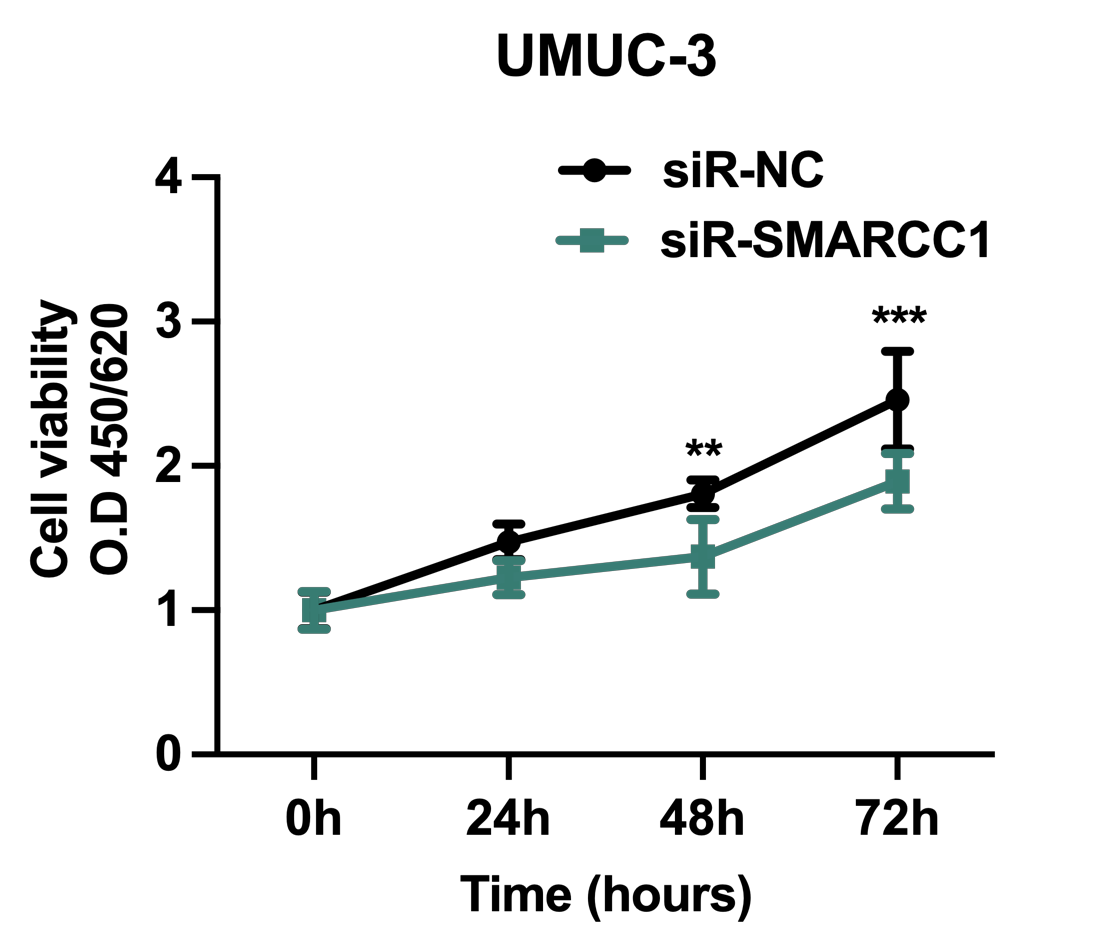

Supplement: Supplementary file 1 [file DataSheet1.ZIP › SMARCC1 RAW data/Figure 4/UMUC-3 cck8.tiff]

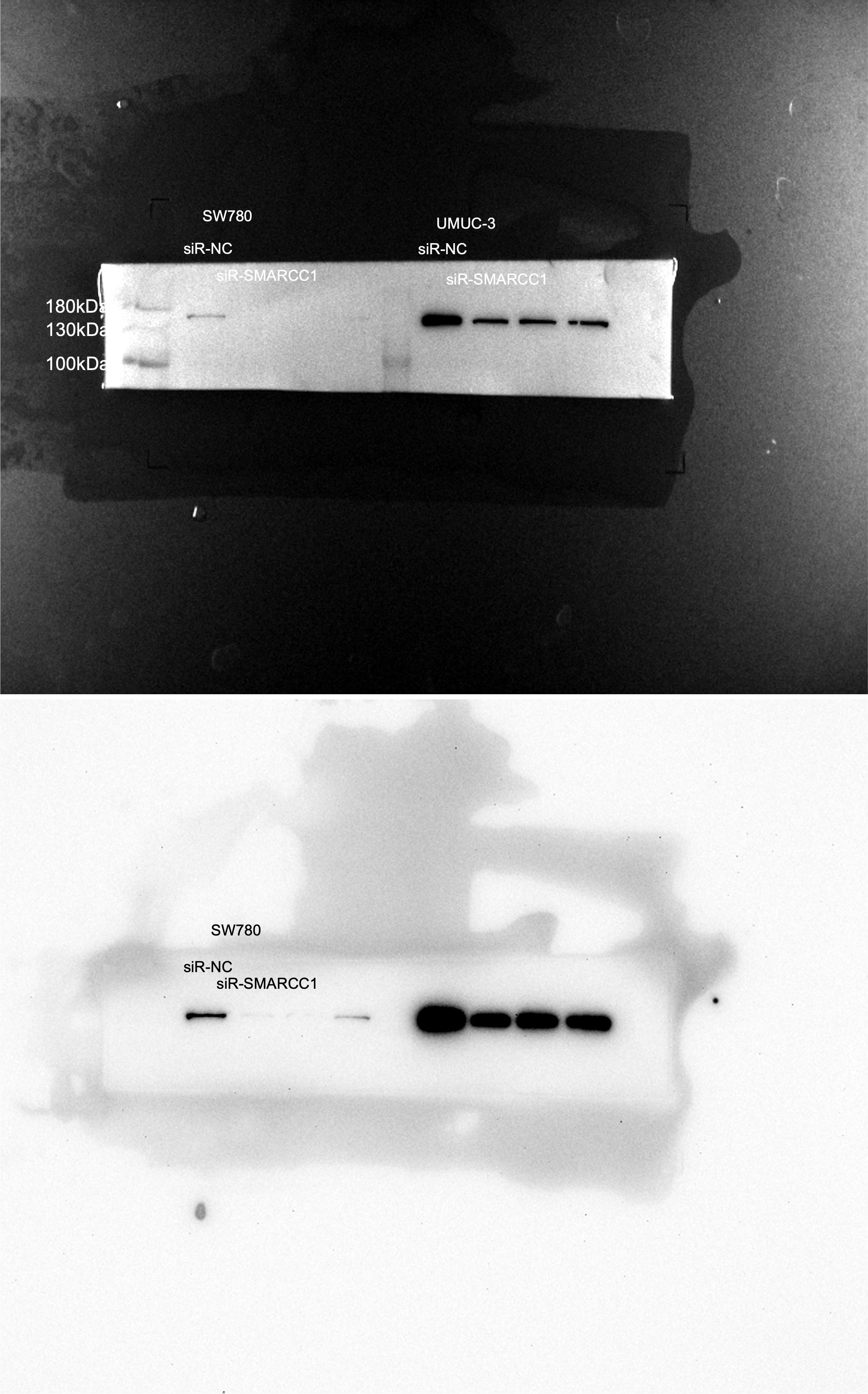

Supplement: Supplementary file 1 [file DataSheet1.ZIP › SMARCC1 RAW data/Figure 4/figure 4 SMARCC1.jpg]

# UMUC3

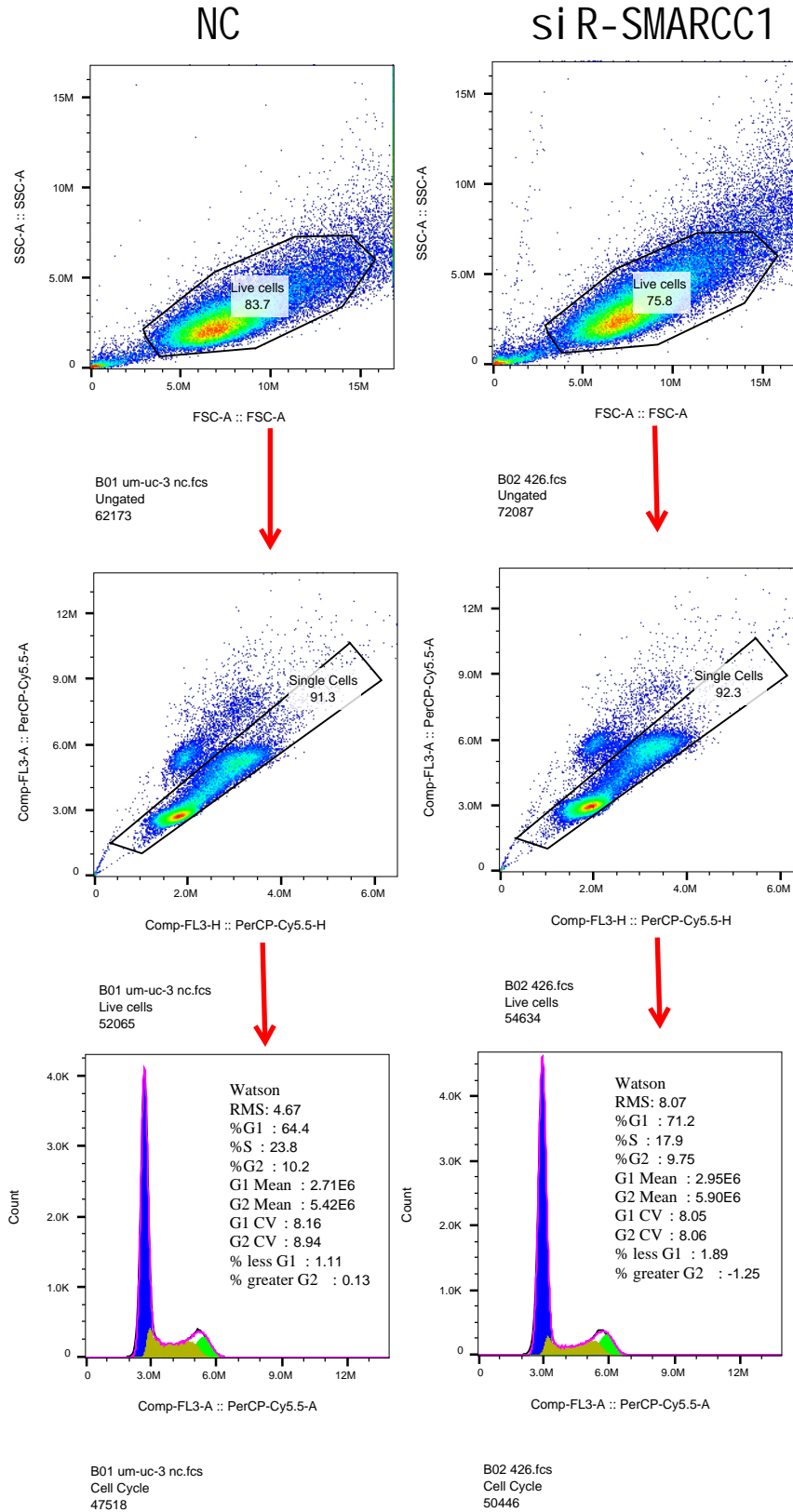

Gating for Figure 4E: Gates are shown sequentially from up to down.

Supplement: Supplementary file 1 [file DataSheet1.ZIP › SMARCC1 RAW data/Figure 4/UMUC-3 cell cycle.pdf]

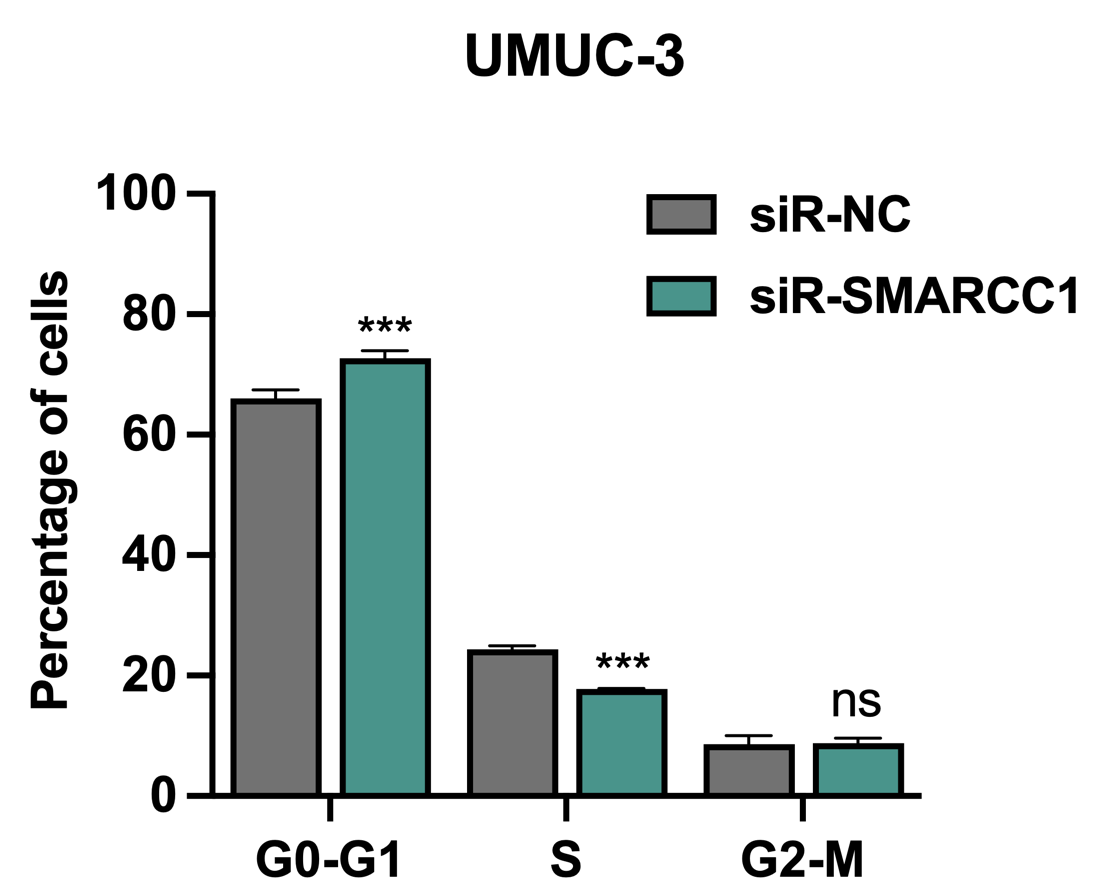

Supplement: Supplementary file 1 [file DataSheet1.ZIP › SMARCC1 RAW data/Figure 4/UMUC-3 Cell Cycle.tiff]

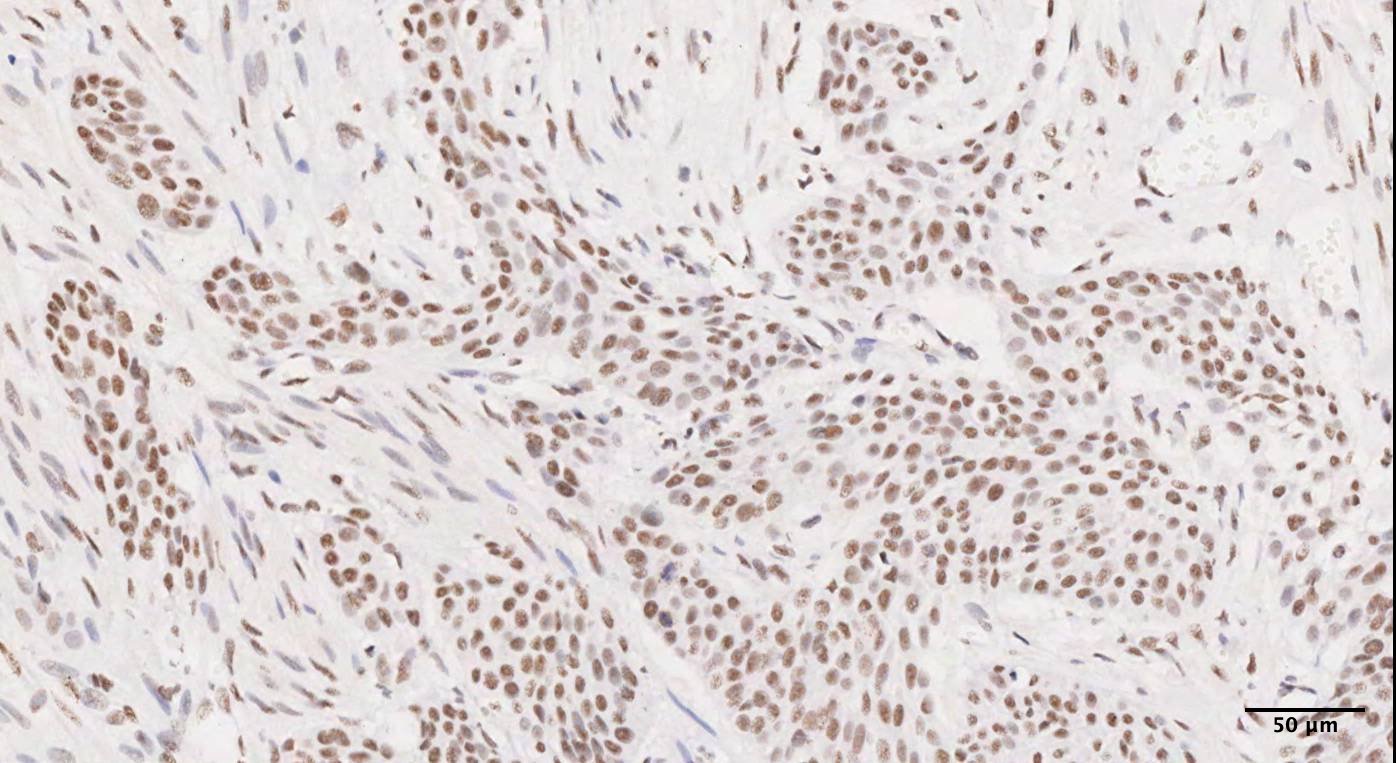

Supplement: Supplementary file 1 [file DataSheet1.ZIP › SMARCC1 RAW data/Figure 3/T2-.jpg]

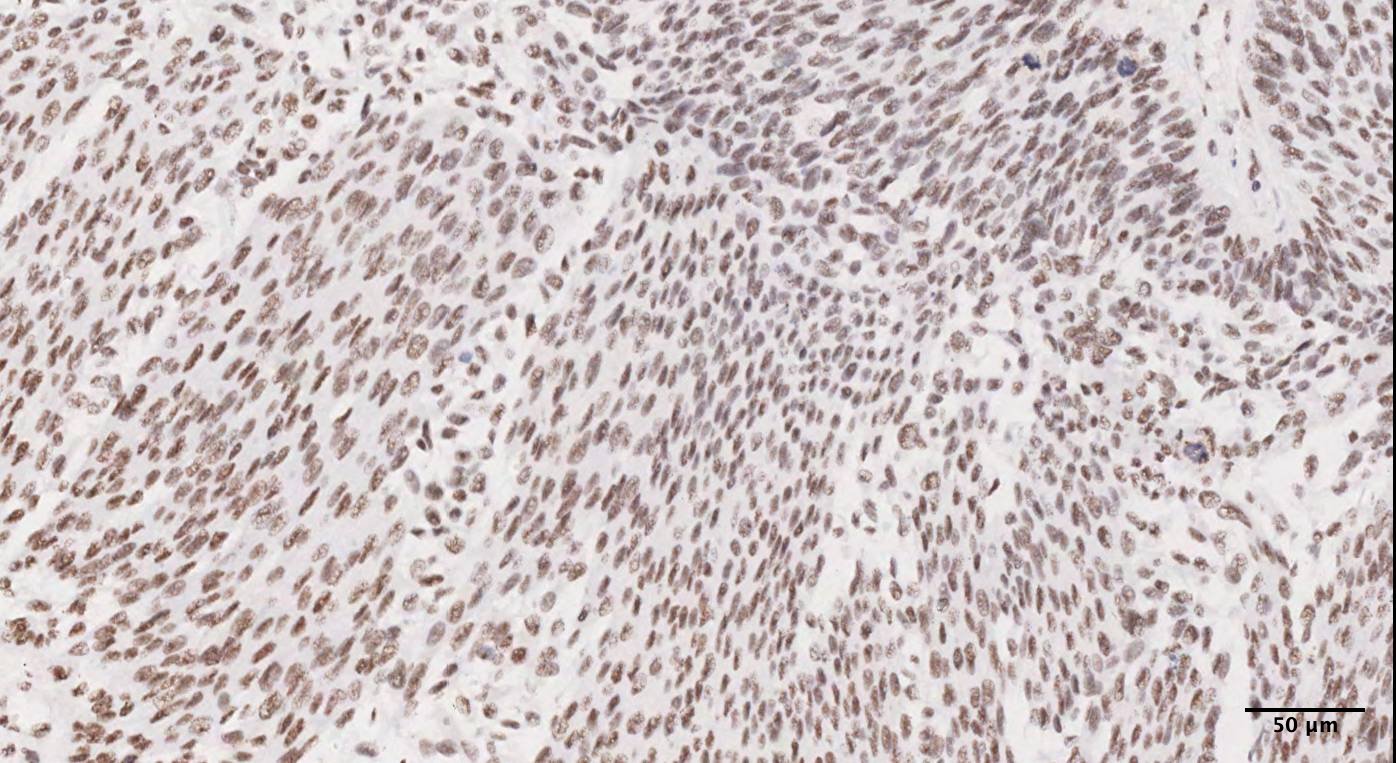

Supplement: Supplementary file 1 [file DataSheet1.ZIP › SMARCC1 RAW data/Figure 3/T3-.jpg]

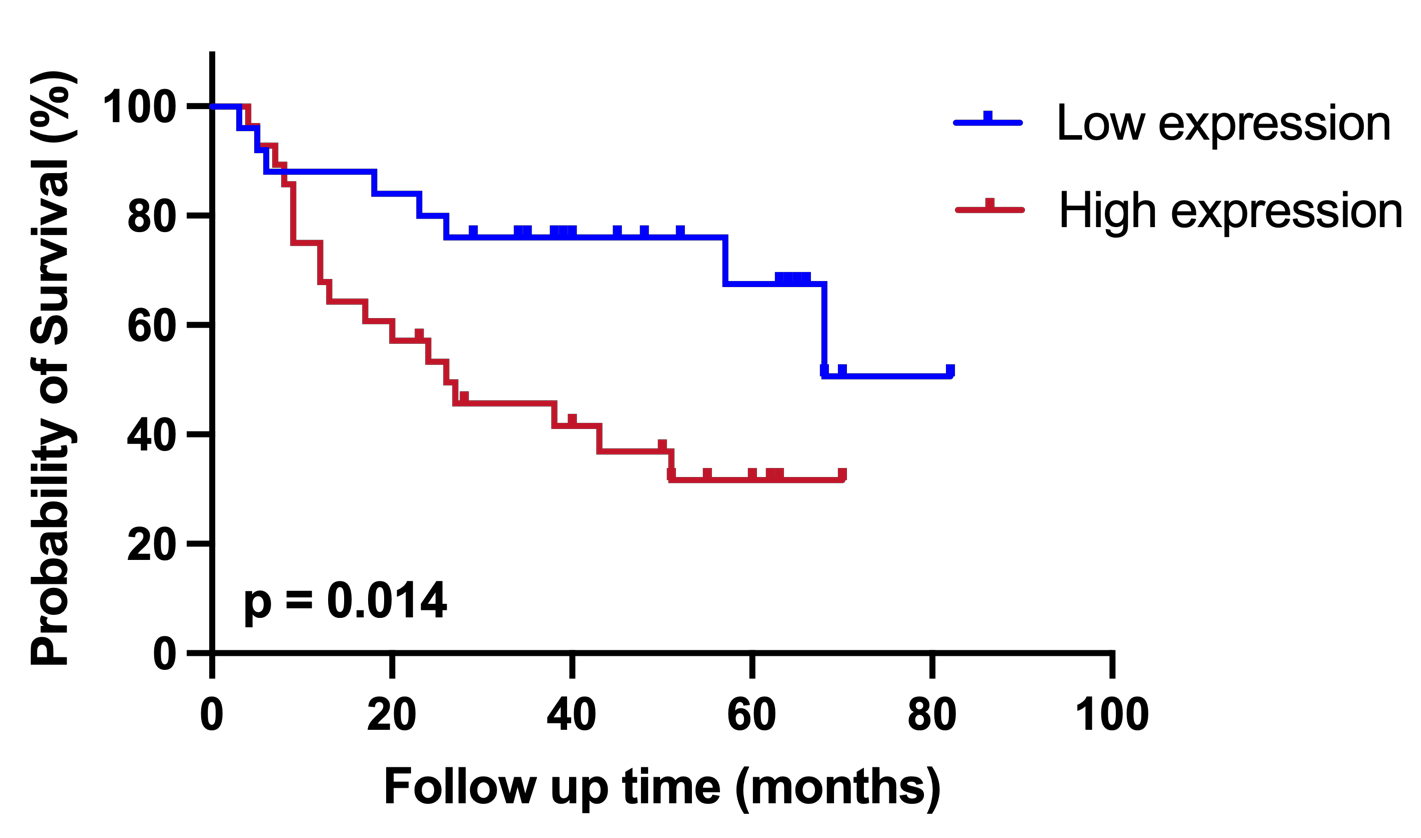

Supplement: Supplementary file 1 [file DataSheet1.ZIP › SMARCC1 RAW data/Figure 3/Survival.tiff]

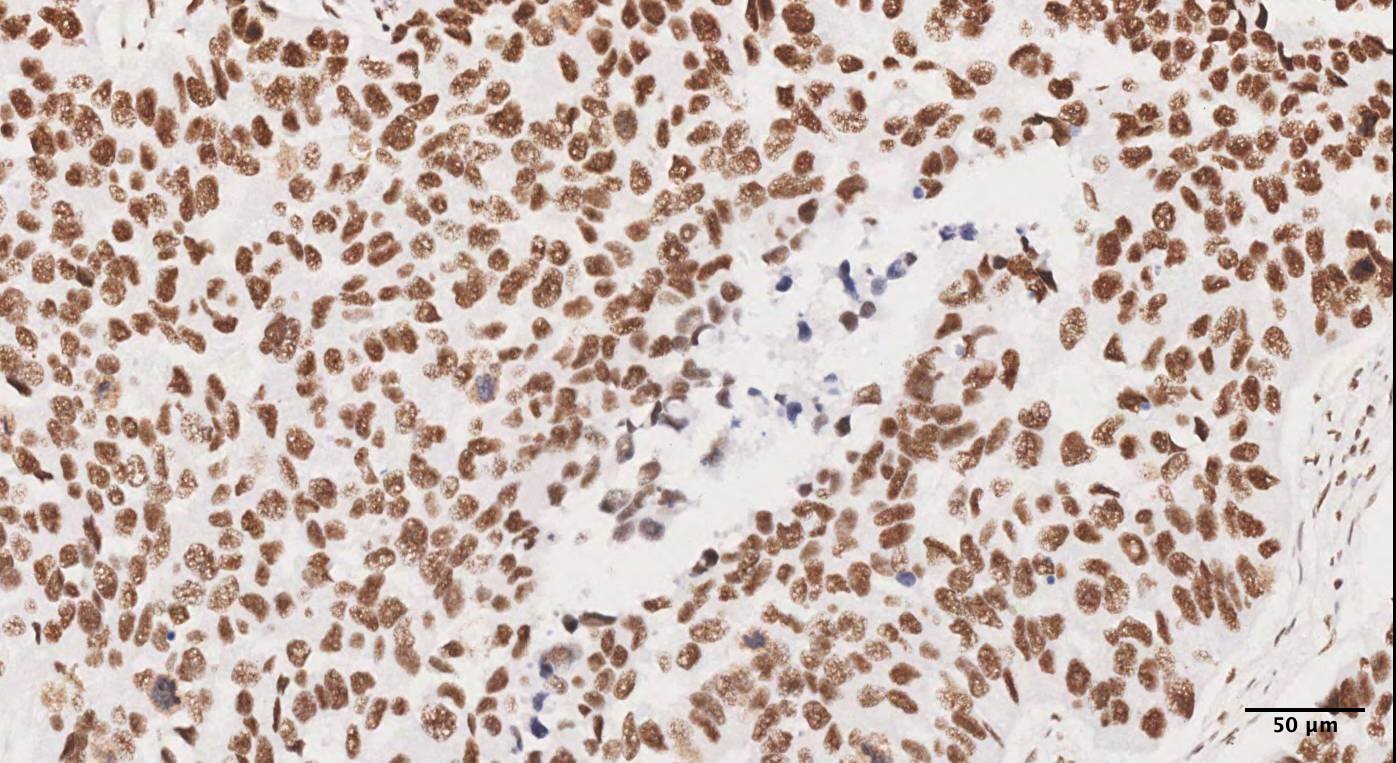

Supplement: Supplementary file 1 [file DataSheet1.ZIP › SMARCC1 RAW data/Figure 3/T4-.jpg]

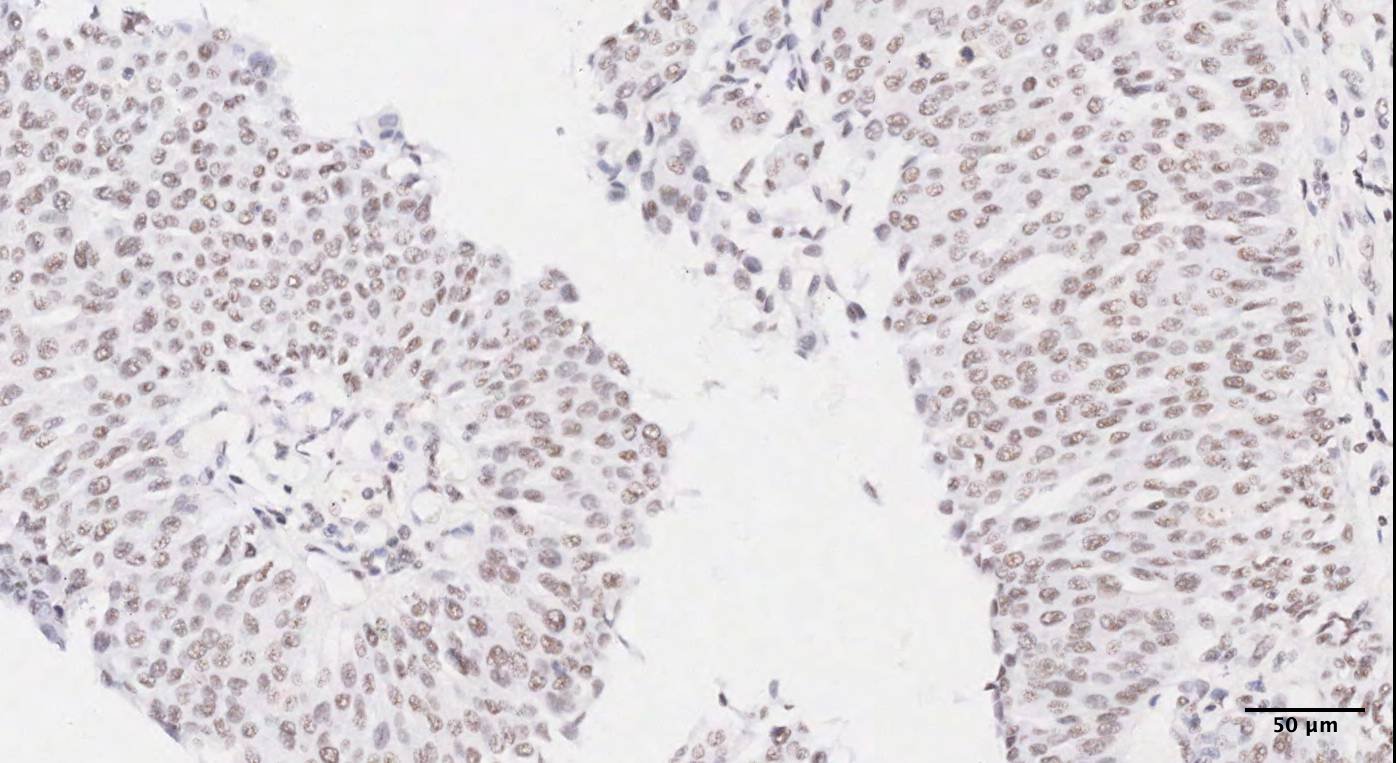

Supplement: Supplementary file 1 [file DataSheet1.ZIP › SMARCC1 RAW data/Figure 3/T1-.jpg]

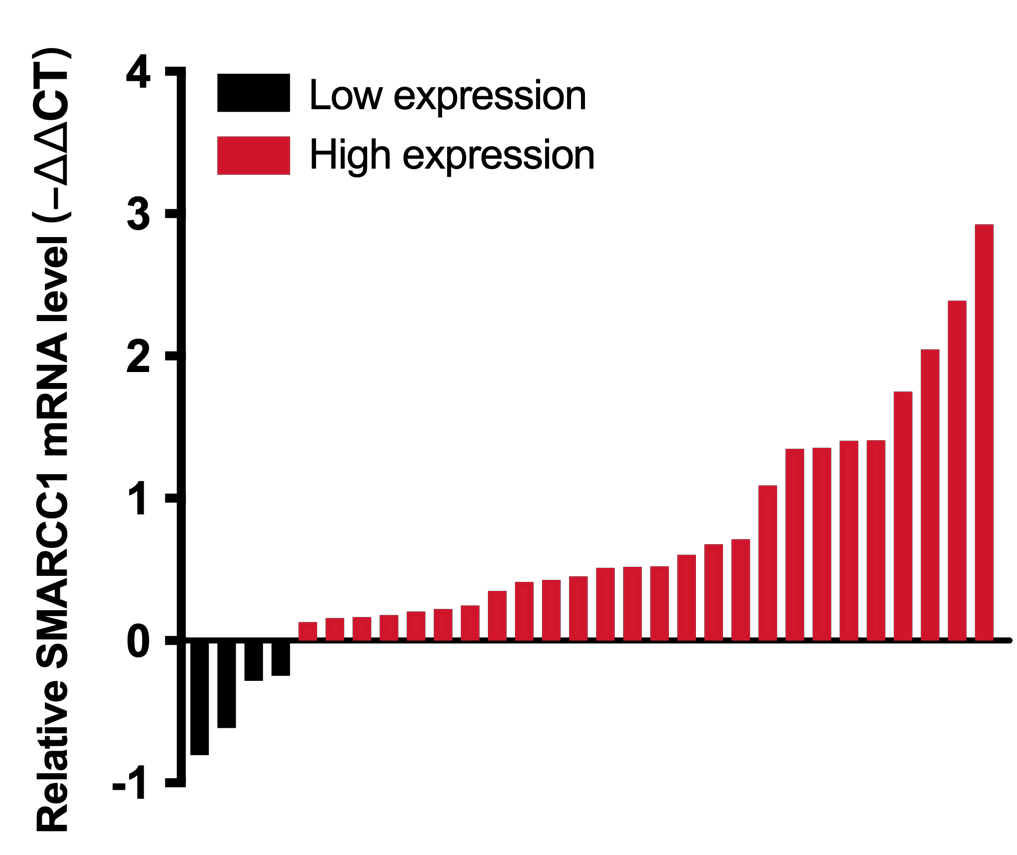

Supplement: Supplementary file 1 [file DataSheet1.ZIP › SMARCC1 RAW data/Figure 2/-╬ö╬öCT of SMARCC1 in 30 pairs BC tissues and adjacent normal bladder tissue.tiff]

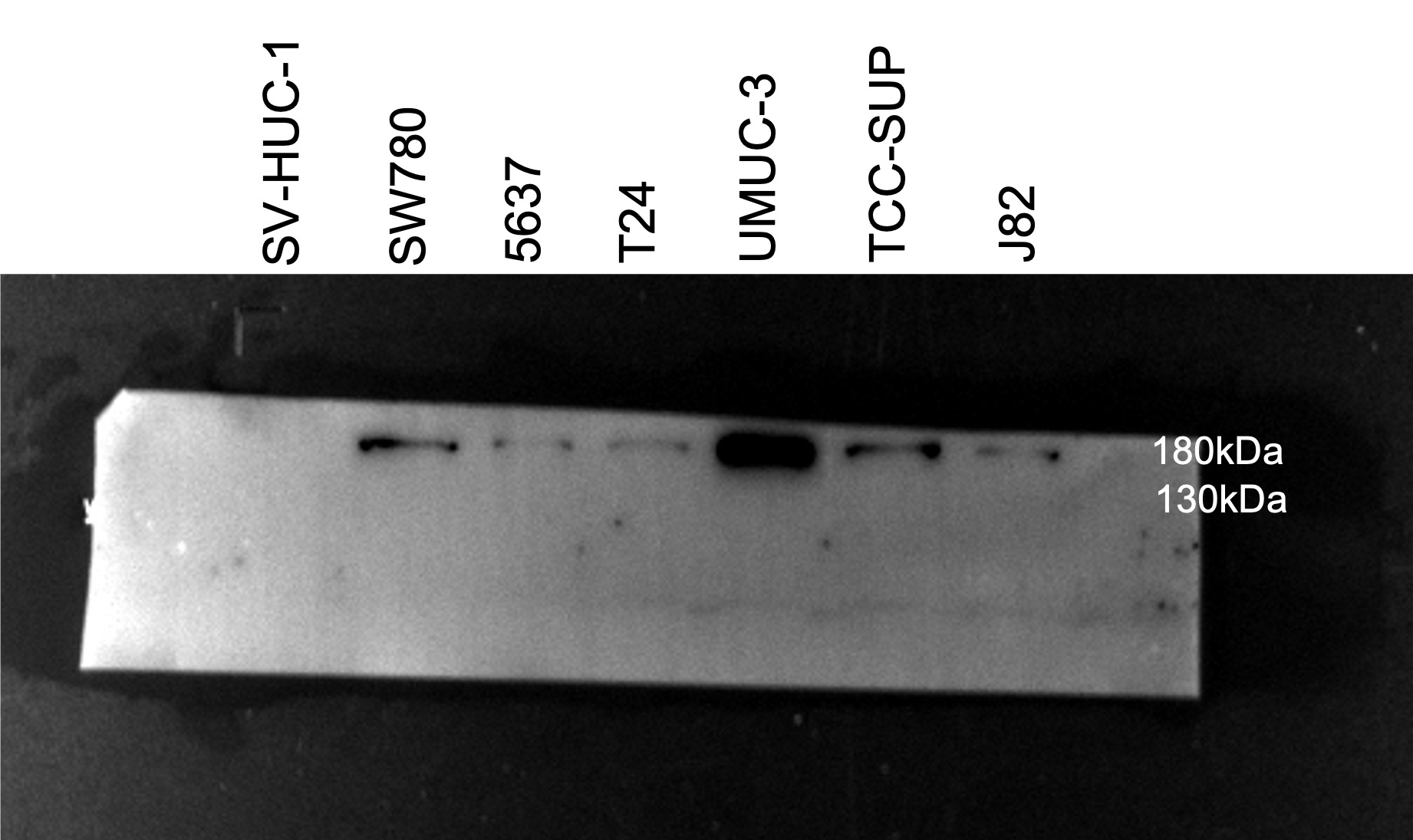

Supplement: Supplementary file 1 [file DataSheet1.ZIP › SMARCC1 RAW data/Figure 2/figure 2D SMARCC1.jpg]

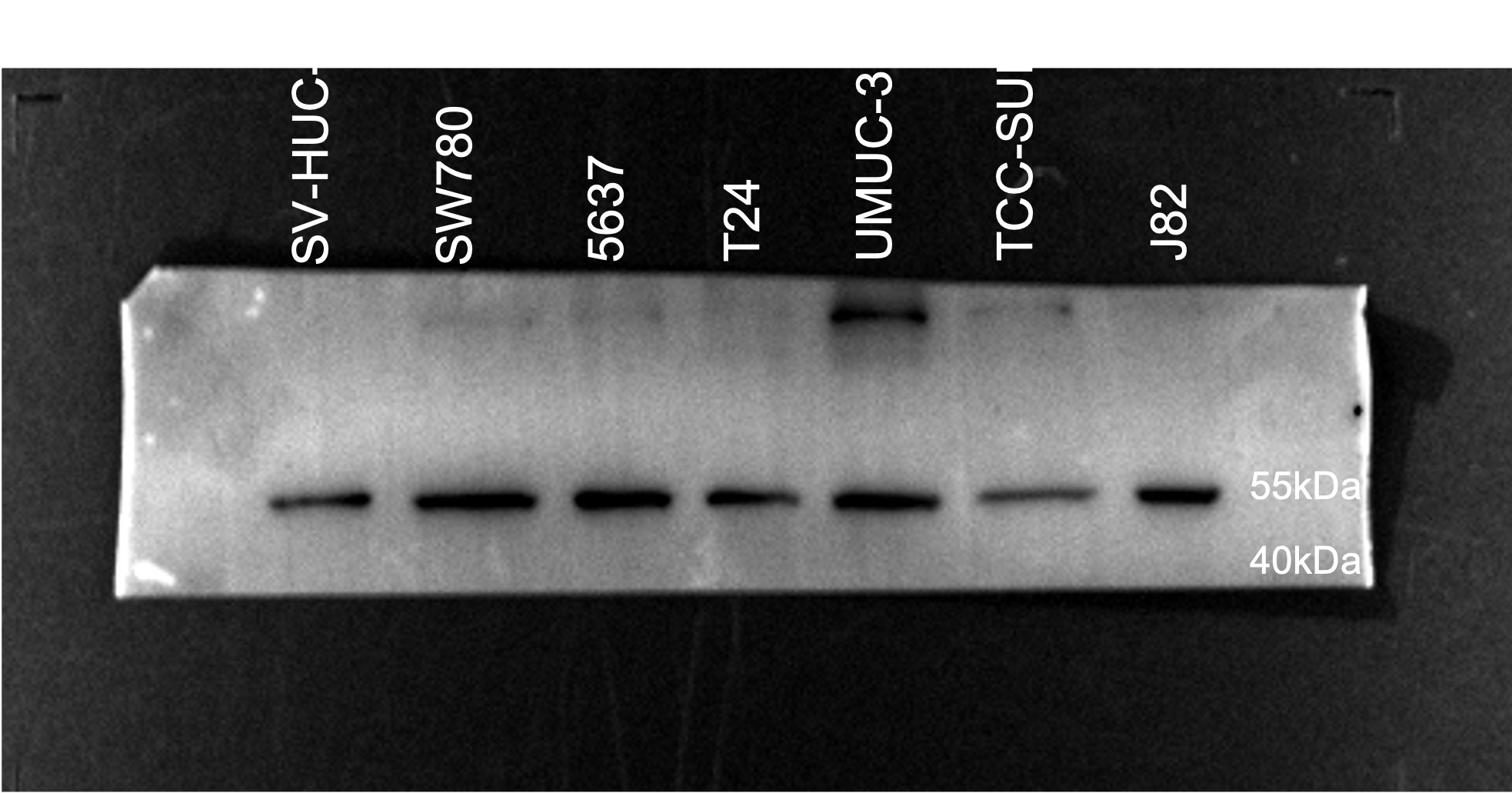

Supplement: Supplementary file 1 [file DataSheet1.ZIP › SMARCC1 RAW data/Figure 2/figure 2D b-tubulin.jpg]

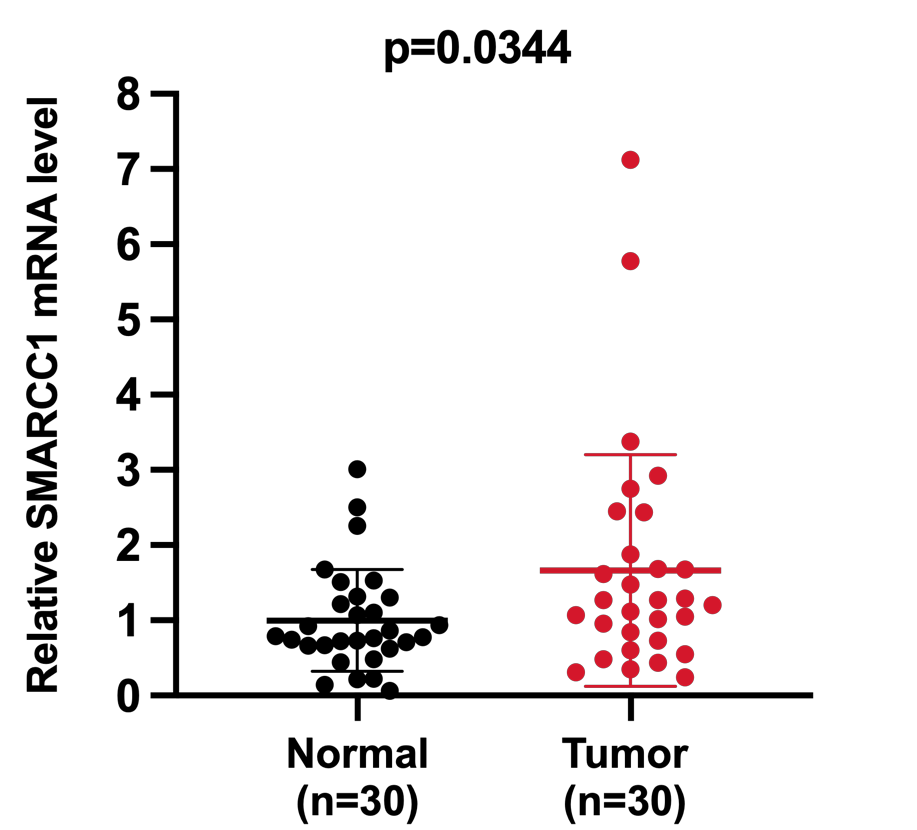

Supplement: Supplementary file 1 [file DataSheet1.ZIP › SMARCC1 RAW data/Figure 2/30 pairs BC tissues.tiff]

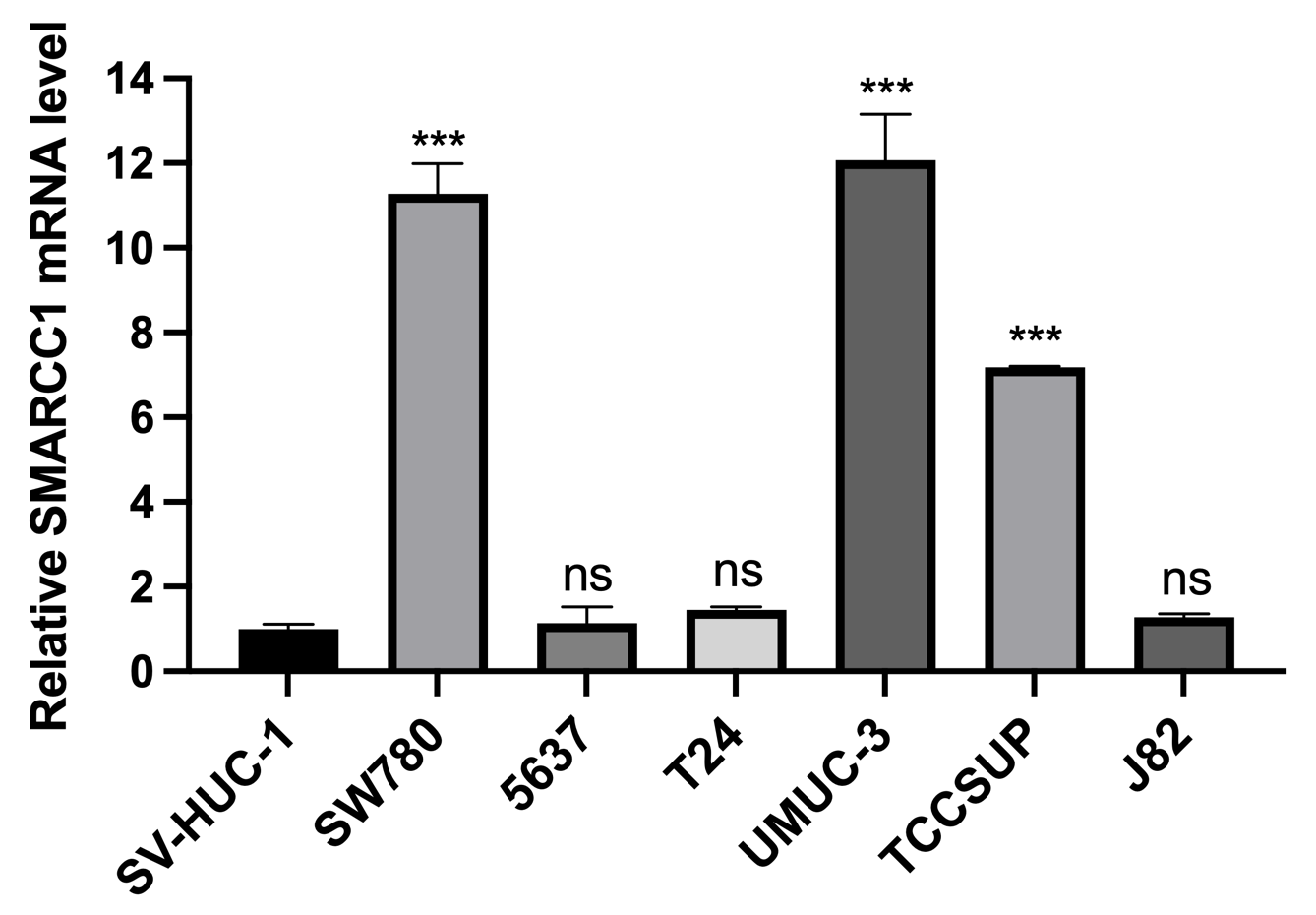

Supplement: Supplementary file 1 [file DataSheet1.ZIP › SMARCC1 RAW data/Figure 2/SMARCC1 in BC cell lines pcr.tiff]

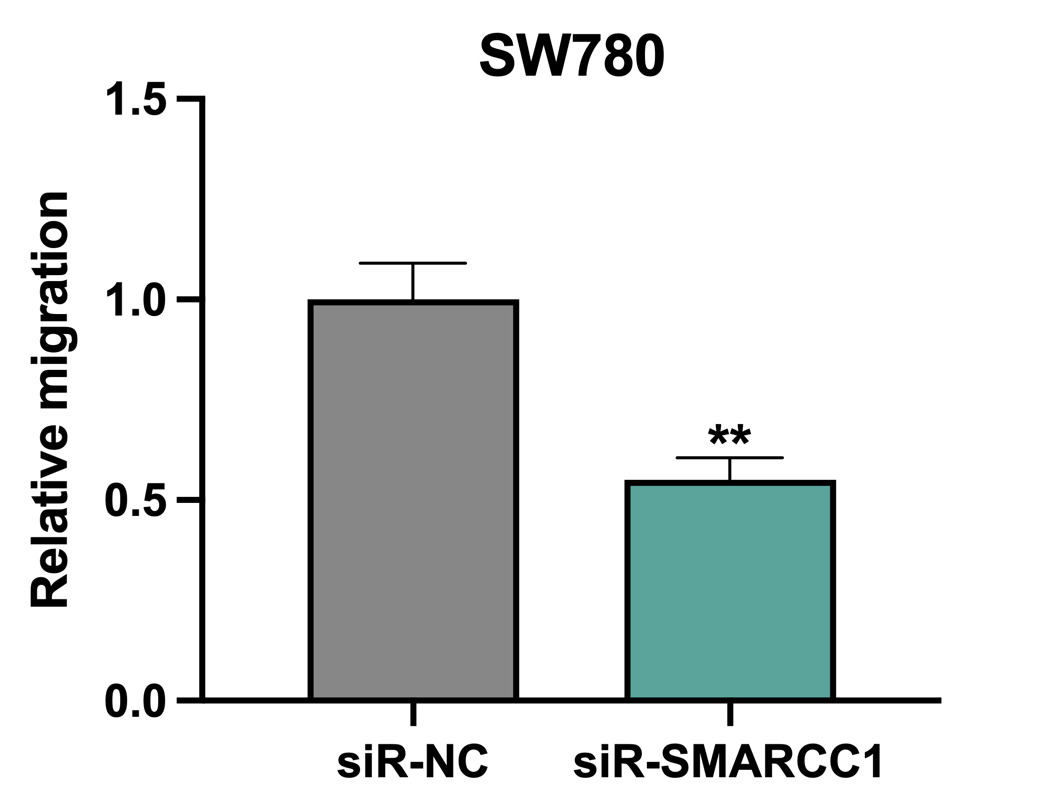

Supplement: Supplementary file 1 [file DataSheet1.ZIP › SMARCC1 RAW data/Figure 5/SW780 migration.tiff]

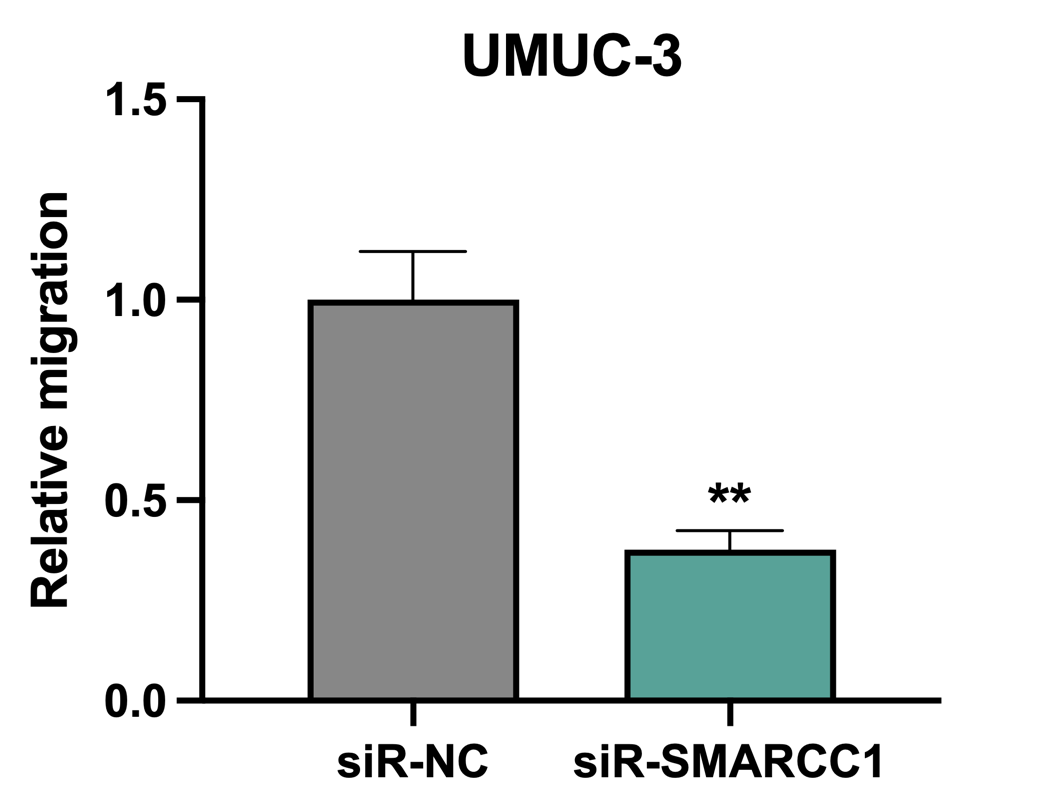

Supplement: Supplementary file 1 [file DataSheet1.ZIP › SMARCC1 RAW data/Figure 5/UMUC-3 migration.tiff]

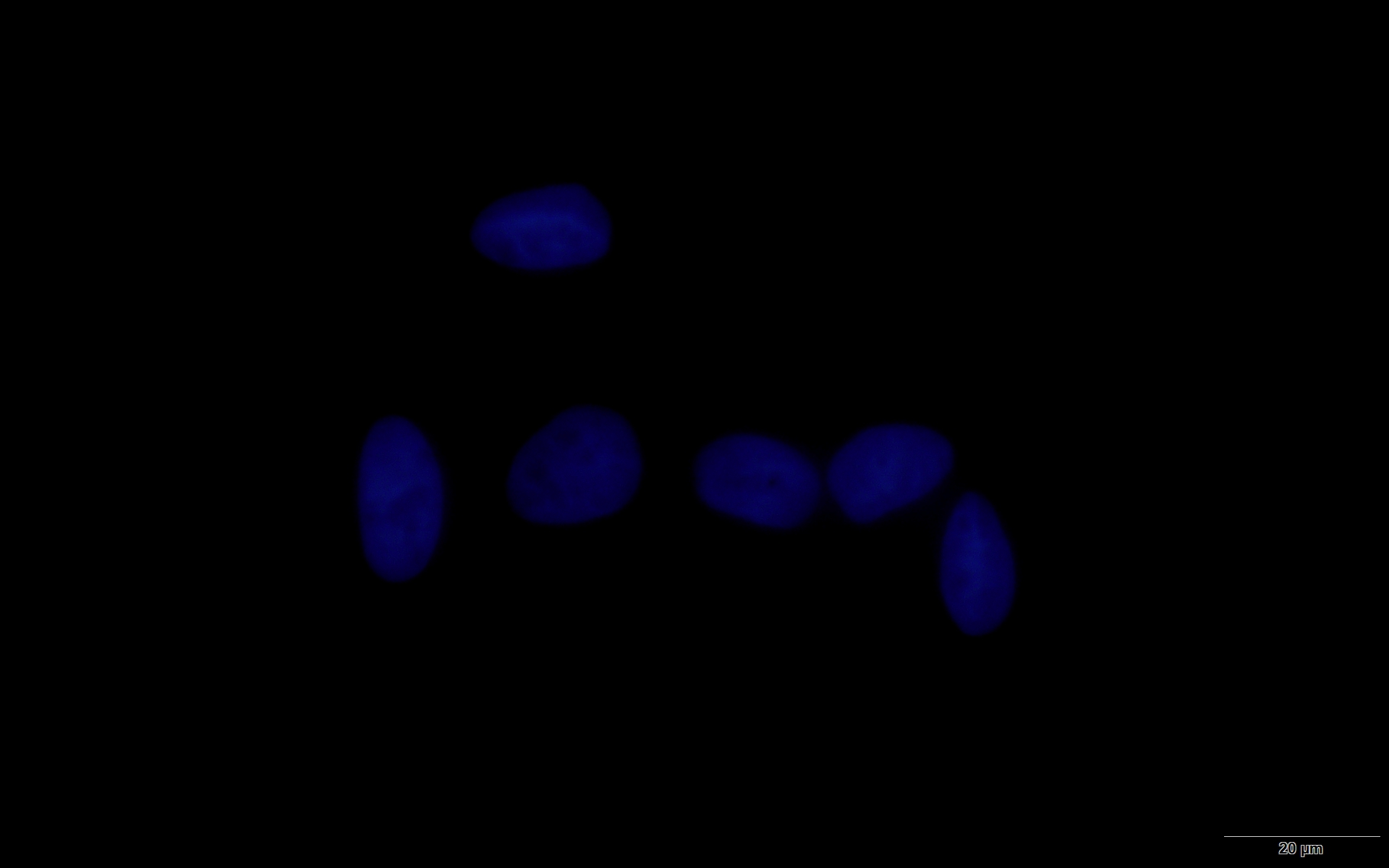

Supplement: Supplementary file 1 [file DataSheet1.ZIP › SMARCC1 RAW data/Figure 1/immunofluorescence staining /immunofluorescence staining for DAPI.jpg]

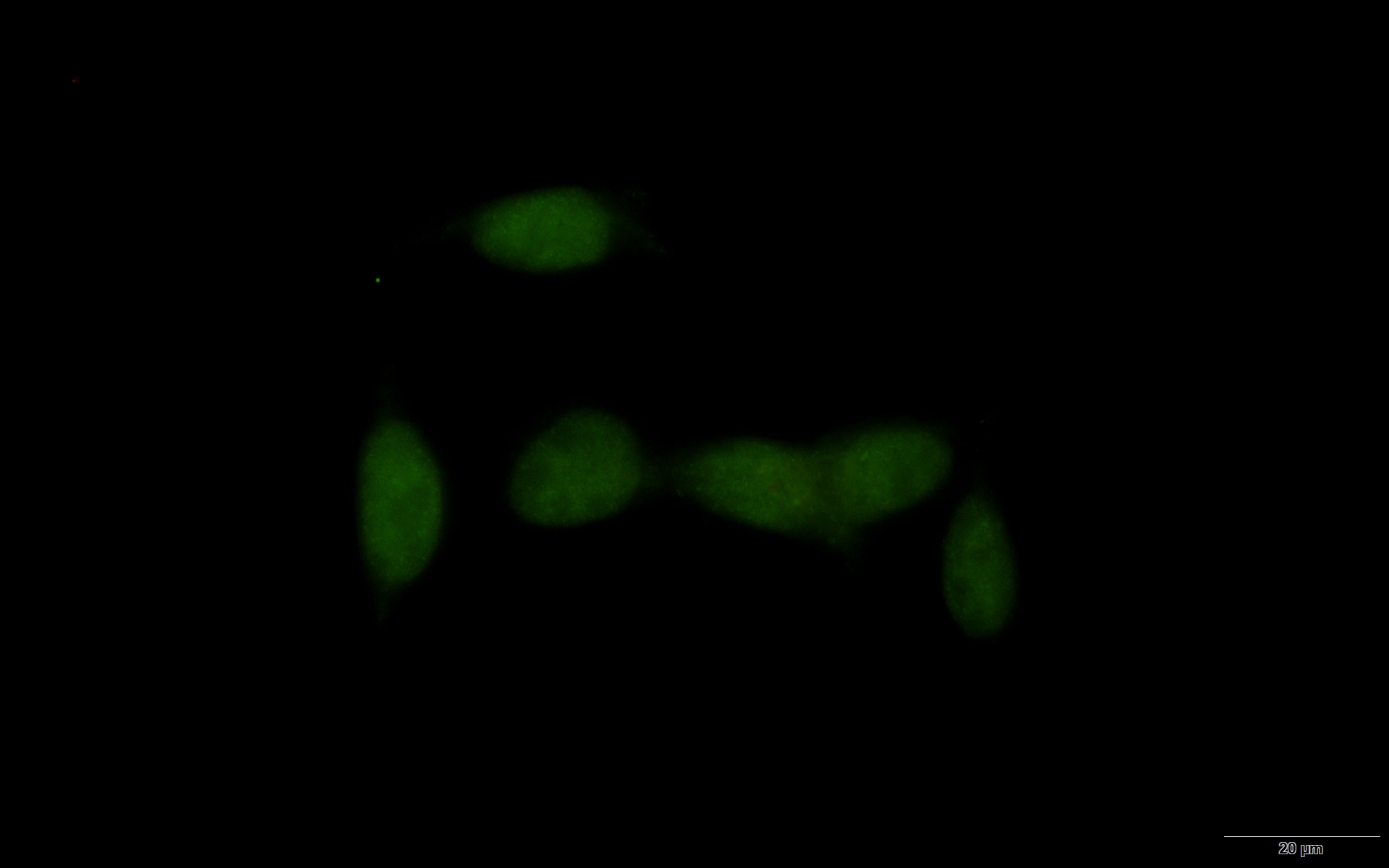

Supplement: Supplementary file 1 [file DataSheet1.ZIP › SMARCC1 RAW data/Figure 1/immunofluorescence staining /immunofluorescence staining for SMARCC1.jpg]

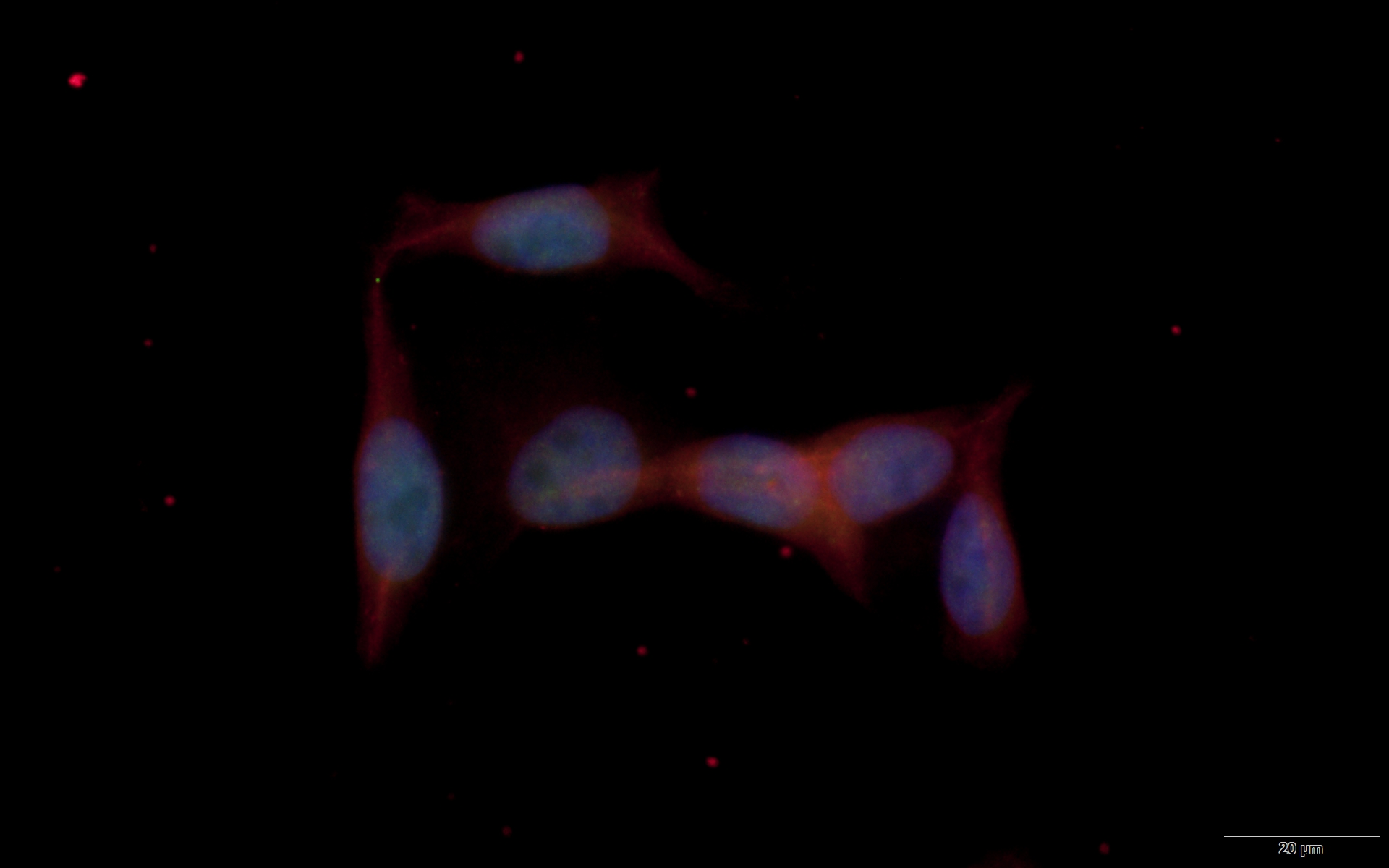

Supplement: Supplementary file 1 [file DataSheet1.ZIP › SMARCC1 RAW data/Figure 1/immunofluorescence staining /immunofluorescence staining for MERGE.jpg]

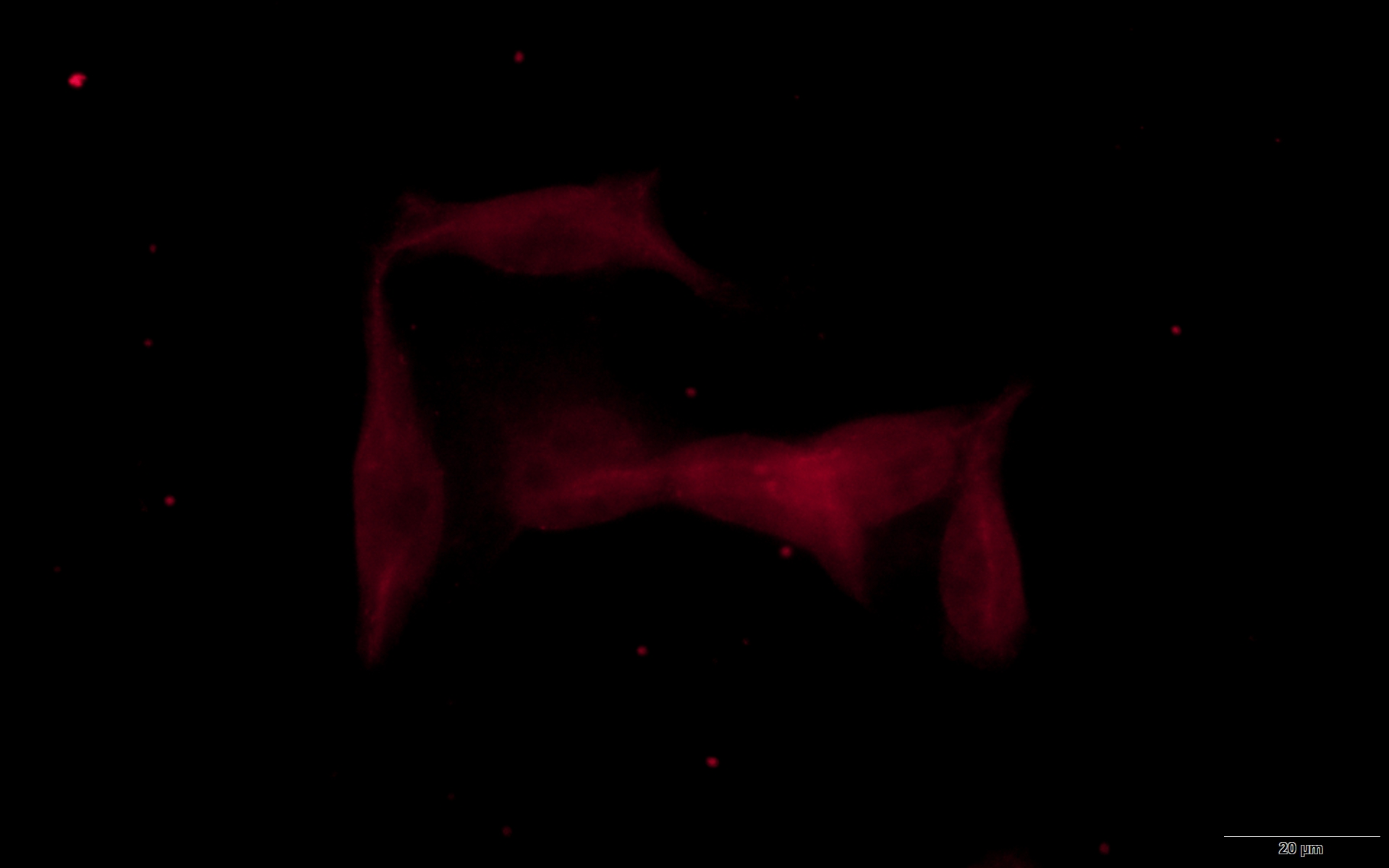

Supplement: Supplementary file 1 [file DataSheet1.ZIP › SMARCC1 RAW data/Figure 1/immunofluorescence staining /immunofluorescence staining for KPNA2.jpg]

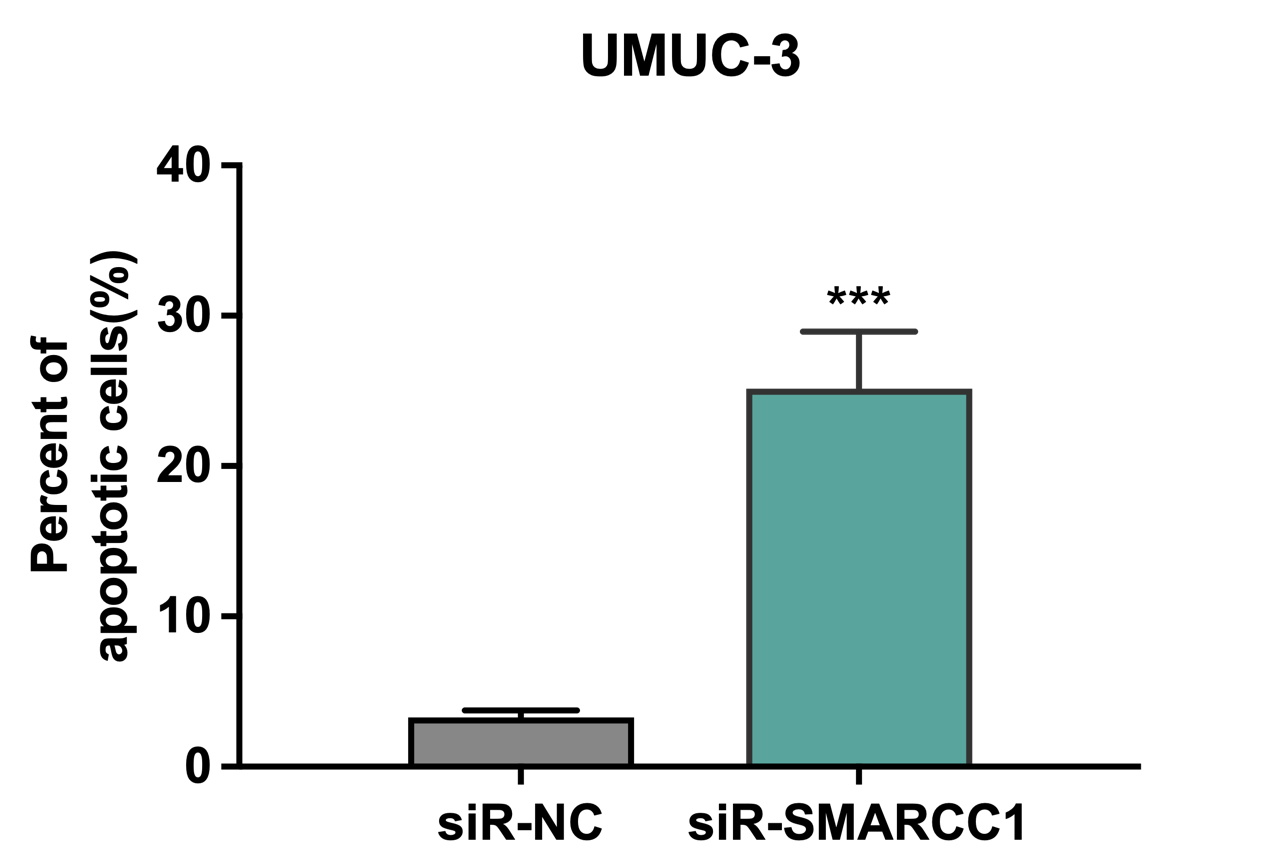

Supplement: Supplementary file 1 [file DataSheet1.ZIP › SMARCC1 RAW data/Figure 5/apoptosis raw data/UMUC-3/UMUC-3 apoptosis.tiff]

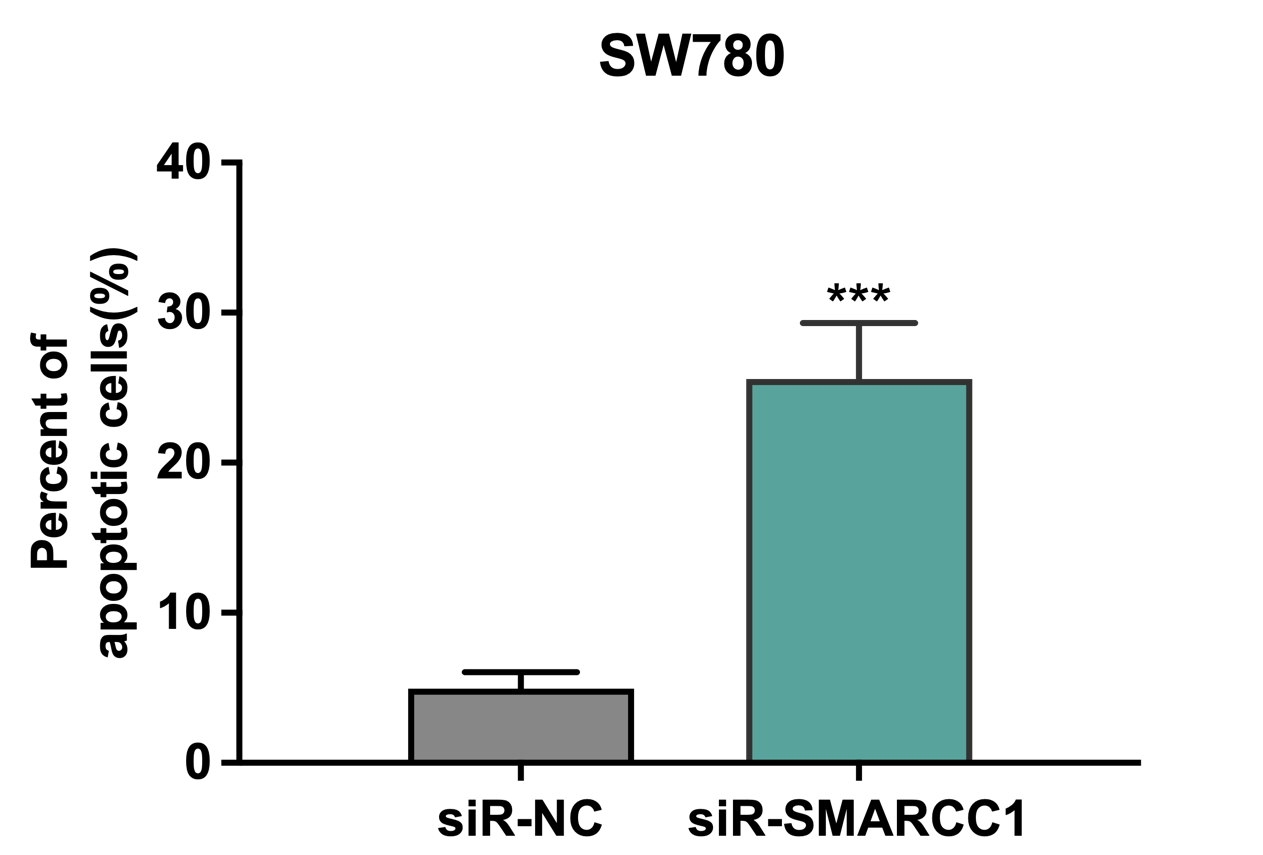

Supplement: Supplementary file 1 [file DataSheet1.ZIP › SMARCC1 RAW data/Figure 5/apoptosis raw data/SW780/SW780 apoptosis.tiff]
