# Supplementary material for: SMARCC1 Enters the Nucleus via KPNA2 and Plays an Oncogenic Role in Bladder Cancer
Source: Front Mol Biosci. 2022 May 20;9:902220. doi: 10.3389/fmolb.2022.902220 (PMC9163745; doi:10.3389/fmolb.2022.902220)
Supplement: Supplementary file 2 [file Image1.pdf]

Supplementary Figure

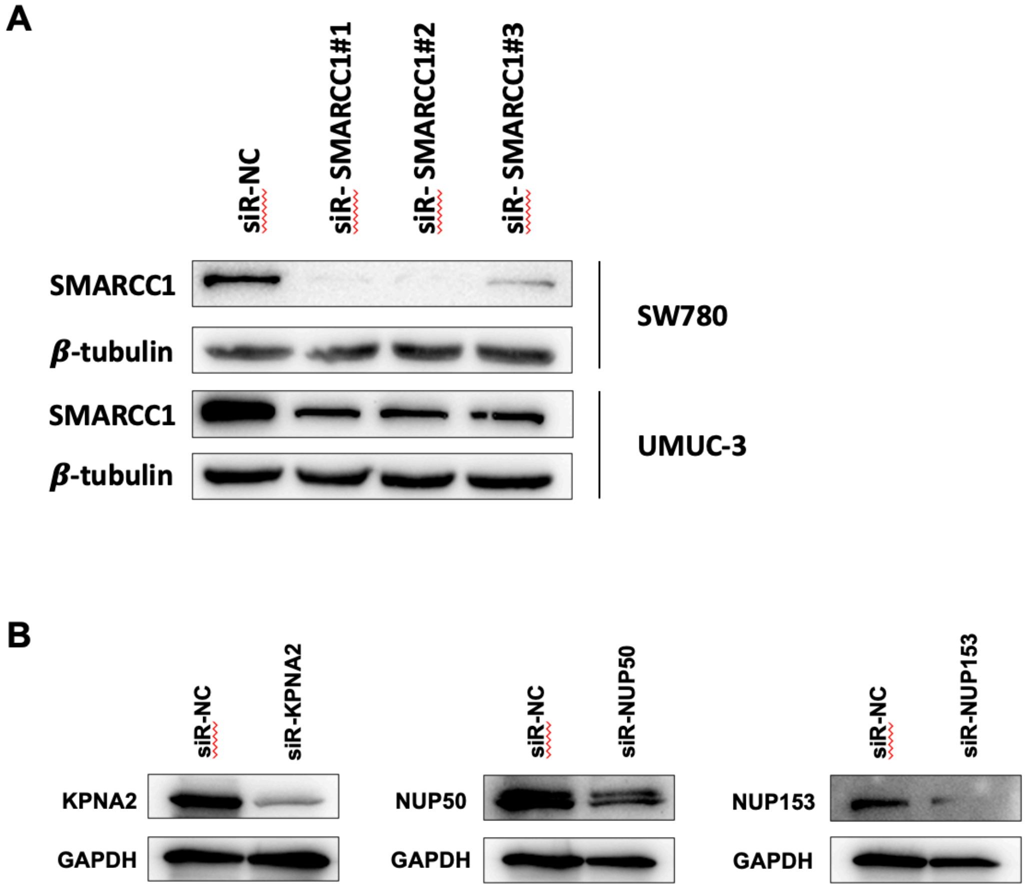

Supplementary Figure S1. Knockdown efficiency of siRNA in bladder cancer cells. (A) The protein level of SMARCC1 in BC cell lines SW780 and UMUC-3 with transfection of siR-SMARCC1 was significantly lower than that in the control group. (B) KPNA2, NUP50, and NUP153 were noteworthy down-regulated in UMUC-3 cell at the translation level after transfection of siR-KPNA2, siR-NUP50, and siR-NUP153, respectively.
